# The emergence of enhanced intelligence in a brain-inspired cognitive architecture

**DOI:** 10.3389/fncom.2024.1367712

**Published:** 2024-05-07

**Authors:** Howard Schneider

**Affiliations:** Sheppard Clinic North, Vaughan, ON, Canada

**Keywords:** brain-inspired cognitive architecture (BICA), planning, neocortex, human-level artificial intelligence (HLAI), artificial general intelligence (AGI), superintelligence

## Abstract

The Causal Cognitive Architecture is a brain-inspired cognitive architecture developed from the hypothesis that the navigation circuits in the ancestors of mammals duplicated to eventually form the neocortex. Thus, millions of neocortical minicolumns are functionally modeled in the architecture as millions of “navigation maps.” An investigation of a cognitive architecture based on these navigation maps has previously shown that modest changes in the architecture allow the ready emergence of human cognitive abilities such as grounded, full causal decision-making, full analogical reasoning, and near-full compositional language abilities. In this study, additional biologically plausible modest changes to the architecture are considered and show the emergence of super-human planning abilities. The architecture should be considered as a viable alternative pathway toward the development of more advanced artificial intelligence, as well as to give insight into the emergence of natural human intelligence.

## 1 Introduction

The Causal Cognitive Architecture (Schneider, 2023, 2024) is a brain-inspired cognitive architecture (BICA). It is hypothesized that the navigation circuits in the amniotic ancestors of mammals duplicated many times to eventually form the neocortex (Rakic, 2009; Butler et al., 2011; Chakraborty and Jarvis, 2015; Fournier et al., 2015; Kaas, 2019; Güntürkün et al., 2021; Burmeister, 2022). Thus, the millions of neocortical minicolumns are modeled in the Causal Cognitive Architecture as millions of spatial maps, which are termed “navigation maps.”

The architecture is not a rigid replication of the mammalian brain at the lower level of the spiking neurons, nor does it attempt to behaviorally replicate the higher-level psychological properties of the mammalian brain. Instead, it considers, given the postulations above, the properties and behaviors that emerge from a cognitive architecture based on navigation maps. The architecture represents a more functionalist system, as per Lieto (2021a,b), although a continuum exists between functionalist and structuralist models. Even in this study, where an enhancement to the architecture is considered, the constraints of biology and anatomy are taken into account.

**Figure 1:**
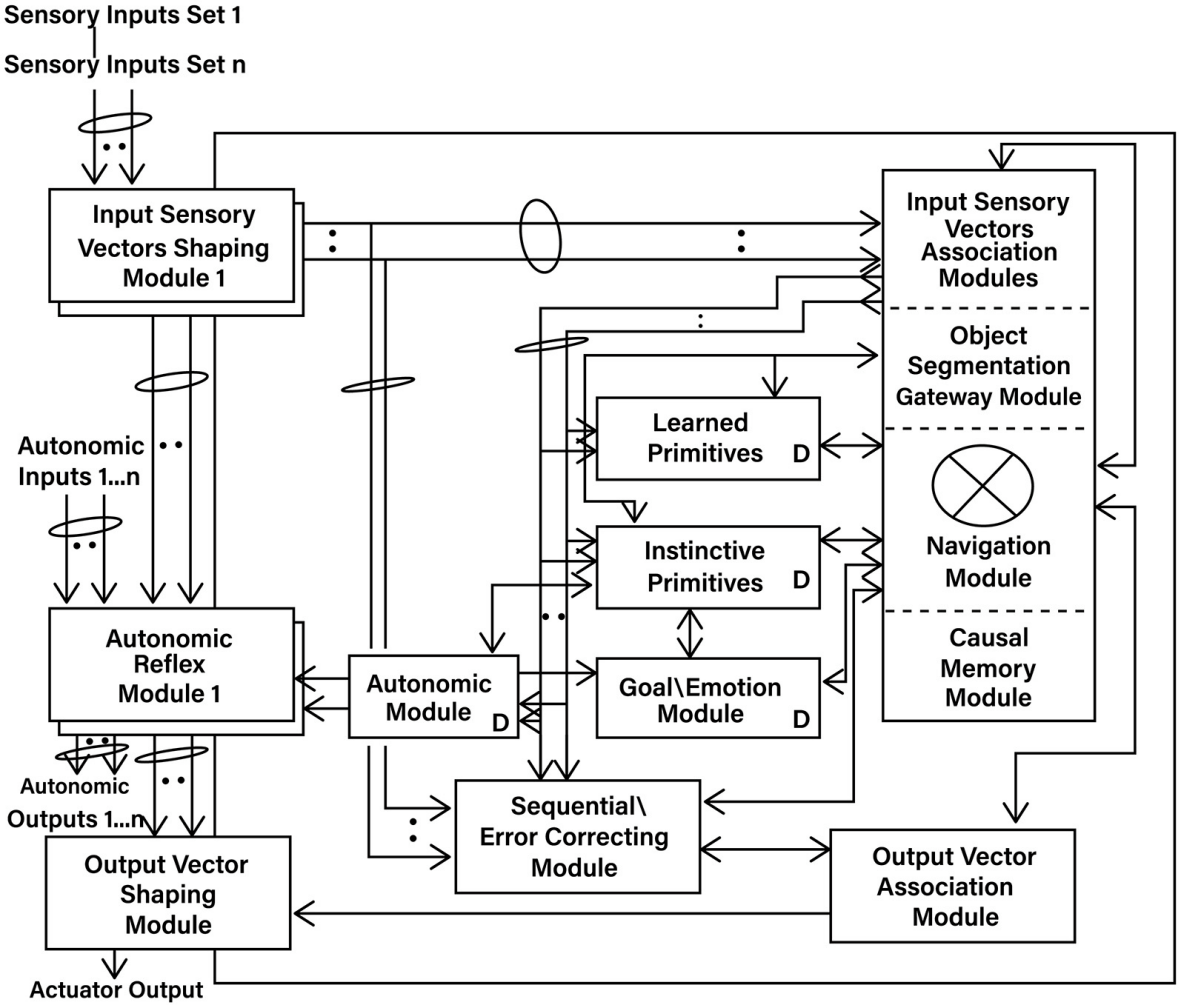
Overview of the Causal Cognitive Architecture 5 (CCA5). See text for a description of modules and their operation (“D” indicates the module has developmental function, i.e., changes algorithms with experience; ovals indicate pathways that are *n* = 0, 1, 2… where there are sets of modules providing signals).

**Figure 2:**
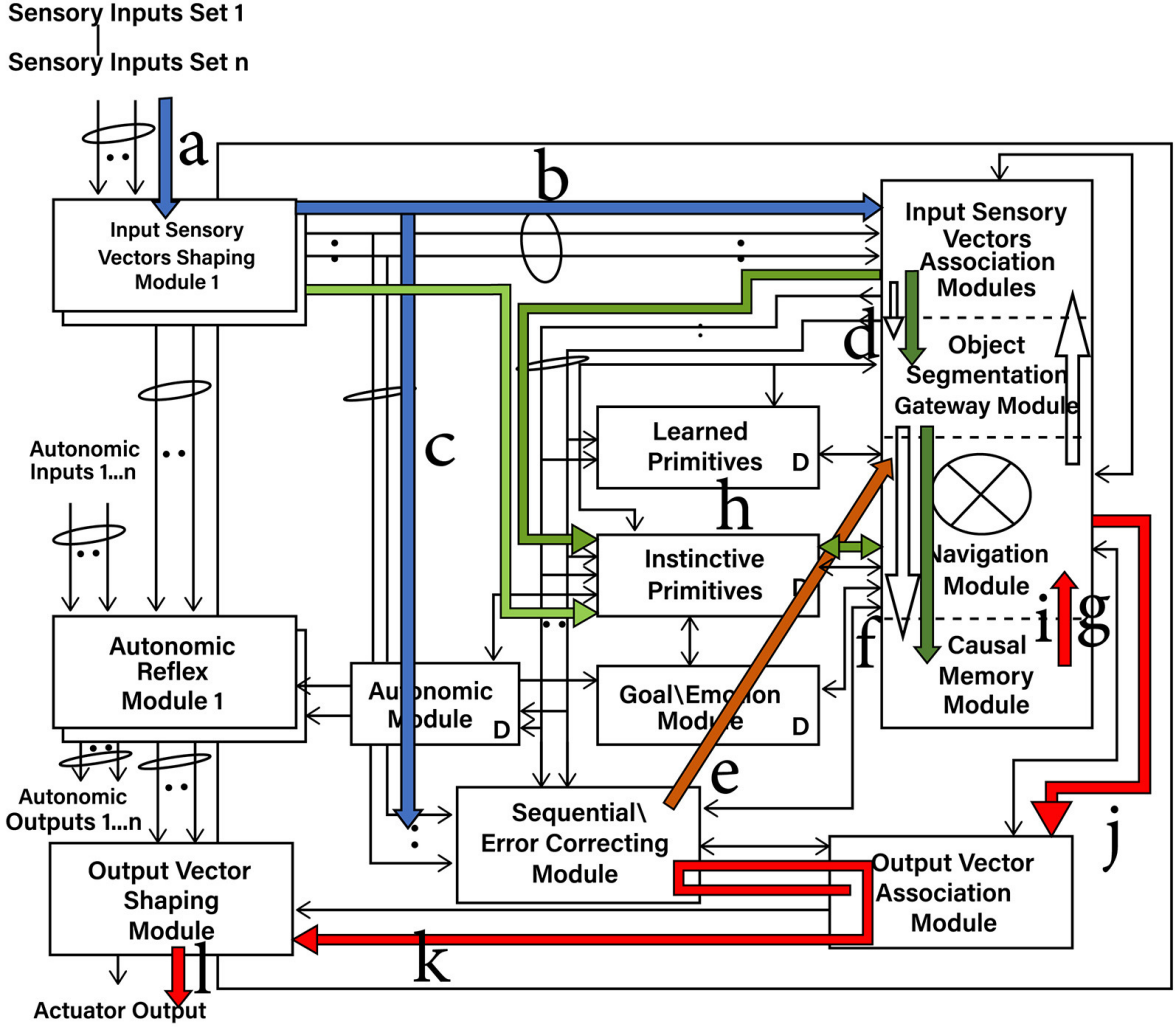
Cognitive cycle: sensory inputs are processed and an output cccurs. a—Sensory inputs stream into the Input Sensory Vectors Shaping Modules. b—Processed and normalized sensory inputs propagate to the Input Sensory Vectors Association Modules and best-matching Local Navigation Maps (LMNs) for each sensory system produced (spatial binding). c—Processed and normalized sensory inputs propagate to the Sequential/Error-Correcting Module (temporal binding). d—Segmentation of objects in the input sensory scenes. e—Spatial mapping of the temporal mapping of the sensory inputs from the Sequential/Error-Correcting Module. f—Match to the best-matching Multi-Sensory Navigation Map from the Causal Memory Module, producing the Working Navigation Map (WNM) for the Navigation Module. g—Working Navigation Map (WNM) in/accessible by Navigation Module. h—Selecting a best-matching primitive (an Instinctive Primitive in this case). i—Instinctive Primitive operating on the Working Navigation Map (WNM). j—*action* signal produced by operation of Instinctive Primitive on WNM. k—***output_vector*** signal, motion corrected by the Sequential/Error Correcting Module. l—Signal to actuators or for electronic transmission.

**Figure 3:**
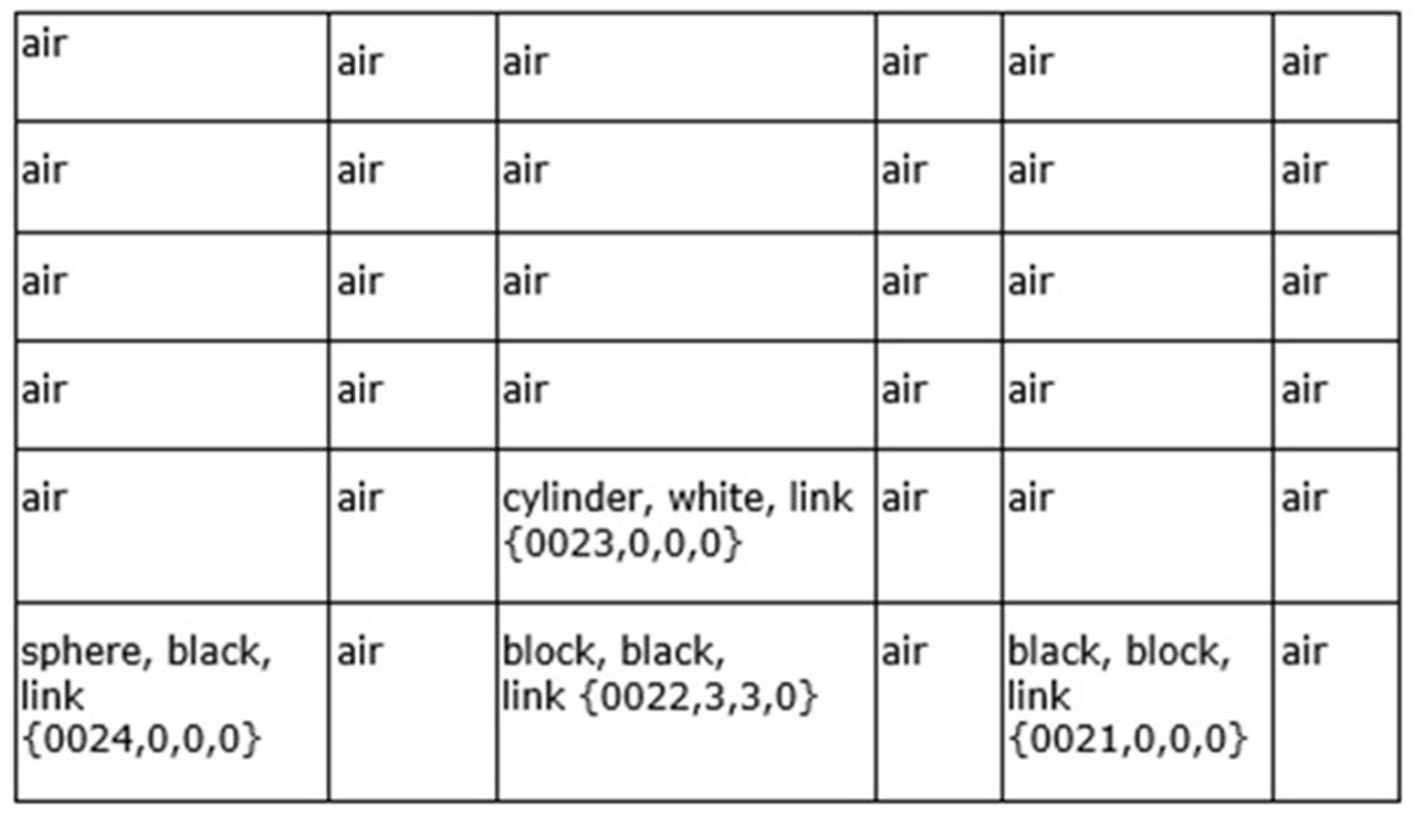
Example of a Navigation Map—the 6x6x0 spatial dimensions are shown, containing sensory features and links to other navigation maps. This represents the sensory scene of Figure 6A. Although this navigation map only contains visual sensory features, other navigation maps can contain combinations of visual, auditory, olfactory, and so on, sensory features.

Figure 1 shows an overview of the Causal Cognitive Architecture 5 (Schneider, 2023). Figure 2 shows the operation of the architecture through a cognitive cycle—a cycle of sensory inputs processed and then an output (or null output) resulting. As noted above, the millions of neocortical minicolumns are modeled in the Causal Cognitive Architecture as millions of spatial maps, termed navigation maps. An example of a simplified navigation map used in a simulation of the architecture is shown in Figure 3. The operation of the Causal Cognitive Architecture is given in more detail below; here, a simple overview of its function is provided.

In each cognitive cycle, sensory inputs stream into the Input Sensory Vectors Shaping Modules (Figure 2). One shaping module exists for each sensory system, e.g., vision, auditory, etc. The shaping module normalizes the sensory inputs into a form that can be used within the architecture. The normalized sensory inputs then move to the Input Sensory Vectors Association Modules. Here, sensory features are spatially mapped onto navigation maps dedicated to one sensory system. Again, there is one association module for each sensory system, e.g., vision, auditory, etc. The navigation maps holding sensory inputs of a given sensory system (e.g., vision) are matched against the best-matching stored navigation map in that (e.g., visual) Input Sensory Vectors Association Module. That best-matching stored navigation map is retrieved and then updated with the actual sensory input of that sensory system (or a new navigation map is created if there are too many changes). As such, there is a type of predictive coding occurring—the architecture anticipates what it is sensing and then considers the differences with the actual input signal. This works well for the perception of noisy, imperfect sensory inputs.

The navigation maps are then propagated to the Object Gateway Modules (Figure 2), where portions and the entire navigation maps are matched against stored multisensory navigation maps (i.e., contain features from multiple sensory systems) in the Causal Memory Module. The best-matched navigation map from the Causal Memory Module is then considered and moved to the Navigation Module. This best-matching navigation map is updated with the actual sensory information sensed from the environment. The updated navigation map is termed the Working Navigation Map. An example of a Working Navigation Map is shown in Figure 3.

Instinctive primitives (pre-programmed) and learned primitives (learned) are small procedures that can perform operations on the Working Navigation Map. They are selected by a similar matching process in terms of the sensory inputs as well as signals from the Goal/Emotion Module and from the previous values of the Navigation Modules. The arrow (i) in Figure 2 shows the actions of the best-matching instinctive primitive on the Working Navigation Map in the Navigation Module. These operations are essentially matrix operations, such as comparing arrays, adding a vector to an array, and other straightforward operations that could be expected of brain-inspired circuitry. The result is an output signal to the Output Vector Association Module and then to the Output Vector Shaping Module. This results in the activation of an actuator or the transmission of an electronics communication signal. Then, the cognitive cycle repeats—sensory inputs are processed again, the Navigation Module may produce an output action, and the cognitive cycle repeats again, and so on.

Feedback pathways, only partially shown in Figures 1, 2, exist throughout the architecture. As noted above, there is a type of predictive coding occurring—the architecture anticipates what it is sensing and then considers the differences with the actual input or intermediate signal. This is advantageous for the perception of noisy, imperfect sensory inputs. In the prior literature, it has been shown how, by enhancing some of these feedback pathways, causal reasoning, analogical inductive reasoning, and compositional language readily emerge from the architecture (Schneider, 2023, 2024). The mechanisms behind these emergent properties are discussed in more detail below.

This study asks what if the evolution of the human brain were to continue as it has in the past, and given an environment for such evolution, what advantageous changes could occur as reflected in a model such as the Causal Cognitive Architecture? More specifically, the study considers an evolution where there are increased intelligence abilities (e.g., Legg and Hutter, 2007; Adams et al., 2012; Wang, 2019; or the ability to better solve complex problems which humans encounter in their lives).

## 2 Previous work: the Causal Cognitive Architecture

A vast number of cognitive architectures exist (Samsonovich, 2010; Kotseruba and Tsotsos, 2020). However, given that this study considers the further biological evolution of the brain modeled via the Causal Cognitive Architecture, this section focuses on the Causal Cognitive Architecture. The purpose of this section is to review the architecture so that the reader is provided with an understanding of its properties and operation before the new work is discussed in this article in the following section. In the last sub-section of this section, the Causal Cognitive Architecture is then compared with other existing cognitive architectures as well as previous work on cognitive maps.

### 2.1 Sensory inputs

In this section, the Causal Cognitive Architecture is considered in more detail. Appendix A gives a more formal description of the Causal Cognitive Architecture. Modified equations are used to describe the architecture. They are “modified” in the sense that many of the equations contain pseudocode. A pseudocode is a common language (e.g., English) description of the logic of a software routine (Olsen, 2005; Kwon, 2014). Traditional pseudocode tends to reproduce program structure, although, in English, it includes constructs such as While, Repeat-Until, For, If-Then, Case, and so on. However, doing so to describe the Causal Cognitive Architecture would be quite lengthy. Using the more abstracted pseudocode in the equations describing the architecture provides a more understandable description of the architecture without sacrificing much accuracy.

The subject of this study is the CCA7 version of the architecture. However, the operation of the CCA5 (Causal Cognitive Architecture 5) version of the architecture will first be described. Then, in the sections further below, the evolution to the CCA6 (Casual Cognitive Architecture 6) version will be briefly considered. Then, the focus of the study will be on the evolution, operation, and properties of the CCA7 version of the architecture. The early sections of the formal description of the Causal Cognitive Architecture 7 (CCA7) in Appendix A apply to the CCA5, CCA6, and CCA7 versions of the architecture.

An overview of the CCA5 version of the architecture is shown in Figures 1, 2. It is seen that sensory inputs stream into the Input Sensory Vectors Shaping Modules, with one module for each sensory system, e.g., vision and auditory. As noted above, the Causal Cognitive Architecture works in terms of cognitive cycles. These cycles are biologically inspired. For example, Madl et al. (2011) hypothesize that the essence of human cognition is cascading cycles of operations. In the CCA5, each cognitive cycle, whatever information in the previous time period of the previous cognitive cycle has streamed into the Input Sensory Vectors Shaping Modules (arrow “a,” Figure 2) is further processed, normalized, and then propagated to the Input Sensory Vectors Association Modules (arrow “b,” Figure 2), as well as to the Sequential/Error Correcting Module (arrow “c,” Figure 2).

The details of sensory perception, i.e., sensory signal processing, from the quantum level to the output produced by a transducer after possibly multi-layered initial signal processing, are largely abstracted away in this formalization. This does not diminish the importance of better signal processing. However, the architecture is concerned with whatever processed sensory inputs stream in, and that is what is considered here.

Equations (9, 10) from Appendix A are shown below. **S**_1, t_ is an array of sensory inputs of sensory system 1 (visual in the current simulation), which has accumulated since the last cognitive cycle to the present cognitive cycle at time t. **S**_2, t_, **S**_3, t_, and so on are arrays of sensory inputs from other sensory systems. Input_Sens_Shaping_Mods.normalize is a method operating on arrays **S** in the Input Sensory Shaping Modules, processing and normalizing the raw input sensory data. **S'** is a normalized array, i.e., the raw array of the streams of sensory inputs for that sensory system has been normalized to a size and form that allows straightforward operations with the navigation maps within the architecture, which are also arrays. The vector ***s'***(*t*) holds the normalized arrays of sensory inputs at time t for the different sensory systems.


(9) $$\begin{array}{l} s\left(t\right)=\left[{\text{S}}_{1,\text{t},}\;{\text{S}}_{2,\text{t},}\;{\text{S}}_{3,\text{t},\ldots ,}\;{\text{S}}_{\text{θ}\_\sigma ,t}\right] \end{array}$$

(10) $$\begin{array}{l} \text{s'}\left(t\right)=\text{Input\_Sens\_Shaping\_Mods.normalize}\left(\text{s}\left(t\right)\right)\\ \;=\left[{\text{S'}}_{1,\text{t},}\;{\text{S'}}_{2,\text{t},}\;{\text{S'}}_{3,\text{t},\ldots ,}\;{\text{S'}}_{\text{θ\_}\sigma ,t}\right] \end{array}$$

In simulations of the CCA5, CCA6, and CCA7 versions of the architecture, visual, auditory, and olfactory simulated inputs have been considered. However, the architecture is very flexible in accepting any number of different sensory system inputs. For example, a radar sensory system (or most other artificial sensory systems) could easily be added to the architecture—its inputs, once processed and normalized, will be treated as any other sensory inputs. Similarly, a particular sensory system can be rendered inoperable, and if the other sensory systems are sufficient, often there will be limited impact on the architecture's final perception and behavior. As will be seen below, there is a very flexible approach toward the processing of sensory input data from a number of different sensory systems.

The Causal Cognitive Architecture is inspired by the mammalian brain. However, to simplify the system of equations used to model the architecture, the olfactory sensory system and any additional non-biological senses are treated the same as the other senses, which in the brain relay through the thalamus to the neocortex. Similarly, the architecture does not model the left-right sides and the interhemispheric movement of working memory in the biological brain.

As can be seen from Figure 1, outputs from the Input Sensory Vectors Shaping Module are also propagated to the Autonomic Reflex Modules. These Reflex Modules perform straightforward actions in response to certain input stimuli, analogous to reflex responses in mammals. In this study, there is more of a focus on the higher-level cognition occurring in the architecture. Similarly, this study does not fully consider or model the repertoire of lower-level learning and behavior routines that exist in humans.

Vector ***s'***(*t*) (Equation 10), holding the normalized arrays of sensory inputs, is propagated to the Input Vectors Association Modules (arrow “b,” Figure 2) and the Sequential/Error Correcting Module (arrow “c,” Figure 2). Spatial binding of the sensory inputs will occur in the Input Vectors Association Modules, i.e., each sensory system's inputs will be mapped onto a navigation map (essentially an array) where the spatial relationship of different sensory inputs are maintained to some extent (e.g., the navigation map in Figure 3 which represents the sensory scene of Figure 6A).

In the Sequential/Error Correcting Module, temporal binding of the sensory inputs occurs—temporal relationships of different sensory inputs, i.e., those of the current cognitive cycle time t = t and those of previous cycles t = t-1 (i.e., 1 cognitive cycle ago), t = t-2, and t = t-3 are bound spatially onto a navigation map and later combined with the spatial navigation maps (Schneider, 2022a,b). This is discussed in more detail in Section A-4 in Appendix A. The need to store snapshots of the input sensory navigation maps every 30th of a second, for example, to track and make memories of motion (as well as changes in higher level cognitive processes), is obviated by transforming the temporal changes into a spatial features that are then mapped on to the same navigation map with the other spatial features.

Temporal binding is an essential feature of the Causal Cognitive Architecture. As mentioned above, it is described in more detail in Appendix A and the literature (Schneider, 2022a). If there is a motion of an object, the temporal binding allows the creation of a vector representing this motion, which can then be mapped onto the existing spatial binding navigation map representing the input sensory data. In addition, the motion of ideas, i.e., changes in navigation maps, can also be similarly mapped onto other navigation maps. However, this study will not focus on temporal binding but rather consider in more detail the flow of sensory inputs ***s'***(*t*) into the Input Sensory Vectors Association Modules and then to the Navigation Module and related modules (Figure 1).

Equation (18) from Appendix A is shown below. In each Input Sensory Vectors Association Module (there is a separate one for each sensory system), the sensory input features (**S'**_σ, *t*_) for that sensory system σ are spatially mapped onto a navigation map. Each such navigation map is then matched (“match_best_local_navmap”) against the best navigation maps stored in that Input Sensory Vectors Association Module for that sensory system σ (***all_maps***_σ, *t*_,). That best-matching stored navigation map is retrieved and then updated with the actual sensory input of that sensory system (or a new navigation map is created if there are too many changes). **LNM**_(σ, γ, t)_ is the updated navigation map (where σ is the sensory system, _γ_ is the map address, and t is the time corresponding to the current cognitive cycle). **LNM** stands for “Local Navigation Map” referring to this being the updated input sensory navigation map for that local sensory system (e.g., vision, auditory, olfactory, and so on). The vector ***lnm***_*t*_ (Equation 23) contains the best-matching and updated navigation maps for each sensory system.

In predictive coding, the brain or artificial agent makes a prediction about the environment, and then this prediction is sent down to lower levels of sensory inputs. Actual sensory inputs are compared to the prediction, and the prediction errors are then used to update and refine future predictions. Essentially, the brain or the artificial agent effectively functions to minimize prediction errors (Rao and Ballard, 1999; Friston, 2010; Millidge et al., 2021; Georgeon et al., 2024).

In Equation (18), **WNM'**_t − 1_ refers to the Working Navigation Map, i.e., the navigation map the architecture was operating on in the Navigation Module (Figure 1) in the previous cognitive cycle t = t-1, which will influence the matching of sensory inputs to the best local sensory navigation map stored in the particular Input Sensory Vectors Association module in the current cognitive cycle t = t. The architecture anticipates to some extent what it will be sensing and then considers the differences with the actual input signal, i.e., which stored navigation map of that sensory system's stored navigation maps (***all_maps***_σ, *t*_) is the closest match based on the actual sensory input (**S'**_σ, *t*_) and based on what the Navigation Module expects to see (**WNM'**_t − 1_). The architecture essentially matches sensory inputs with navigation maps it already has experience with and then considers the differences (i.e., the error signal) with updated navigation maps (i.e., error signal resolved) saved in memory. This works well for the perception of noisy, imperfect sensory inputs, and this is easy to implement with the navigation map data structure (e.g., Figure 3) used by the architecture.

While this process has a number of similar aspects to predictive coding, it was not designed as a predictive coding architecture. For example, the architecture does not actively attempt to minimize free energy or minimize prediction errors, although this effect often results. Instead, this arrangement emerged in the attempt to model the evolution of the brain, albeit in a loosely functionalist approach (e.g., Lieto, 2021b).


(18) $$\begin{array}{c} \text{LN}{\text{M}}_{\left(\sigma ,\gamma ,\text{t}\right)}=\text{Input}\_\text{Assocn}\_{\text{Mod}}_{\sigma }.\text{match}\_\text{best}\_\text{local}\\ \_\text{navmap}\;({{\text{S}}^{\prime }}_{\sigma ,t},\text{all}\_\text{map}{\text{s}}_{\sigma ,t},\text{WN}{{\text{M}}^{\prime }}_{\text{t-1}}) \end{array}$$

(23) $$\begin{array}{l} {\text{lnm}}_{t}=\left[{\text{LNM}}_{\left(1,\gamma ,\text{t}\right)},{\text{LNM}}_{\left(2,\gamma ,\text{t}\right)},{\text{LNM}}_{\left(3,\gamma ,\text{t}\right)},\ldots ,{\text{LNM}}_{\left(\theta \text{\_}\sigma ,\gamma ,\text{t}\right)}\right] \end{array}$$

The Local Navigation Maps [i.e., the best-matching navigation maps for each “local” sensory system updated by the actual sensory inputs, represented by ***lnm***_*t*_ in Equation (23)] are then propagated to the Object Gateway Modules (arrow “d,” Figure 2), with one module for each sensory system. In the Object Gateway Modules, portions of each navigation map will be attempted to be segmented into different objects, as best it can do. If there is, for example, a black sphere in a sensory scene, the Object Gateway Module, when processing the visual LNM (local navigation map), will readily segment out a black sphere from the rest of the sensory scene [the navigation map shown in Figure 3 was constructed in this manner from various visual close-up sensory inputs. For example, in cell (0,0,0), the link shown, i.e., {0024, 0,0,0}, refers to another navigation map where the various lines and colors of a sphere were extracted from the navigation map as a black sphere, and where the descriptive labels “sphere” and “black” were linked to].

The temporal binding (i.e., motion) of the sensory inputs that have occurred in the Sequential/Error Correcting Module is spatially mapped to each of the Local Navigation Maps (arrow “e,” Figure 2). For example, Equation (62) taken from Appendix A shows the Local Navigation Map for the visual sensory inputs **LNM**_(1, γ, t)_ being updated with a “Vector Navigation Map” “**VNM**”_t_ (i.e., the motion prediction vector created in the Sequential/Error Correcting Module and applied to an array), with the updated Local Navigation Map **LNM'**_(1, γ, t)_ resulting as follows:


(62) $$\begin{array}{l} {\text{LNM'}}_{\left(1,\gamma_{,t}\right)}={\text{LNM}}_{\left(1,\gamma ,t\right)}\;\cup \;{\text{VNM”}}_{t} \end{array}$$

The different sensory system-updated Local Navigation Maps **LNM'**_(1…., γ*, t*)_ are then matched against the multisensory (i.e., have features from all sensory systems as well as perhaps other features created and stored on the maps) navigation maps stored in the Causal Memory Module (arrow “f,” Figure 2). **WNM**_*t*_ is the best-matching multisensory navigation map. Equation (67) from Appendix A shows that the Object Segmentation Gateway Module (“Object_Seg_Mod”) built-in method (i.e., part of the circuitry of the Object Segmentation Gateway Module) “differences” compares the number of differences between actual sensory information on the Local Navigation Maps represented by ***actual***_*t*_ to the features represented by **WNM**_*t*_.

As Equation (67) shows, if the number of differences is low enough, then the best-matching multisensory navigation map **WNM**_*t*_ is updated with actual sensory information from the Local Navigation Maps represented by ***actual***_*t*_ [if there are too many differences between the best-matching map and the actual input maps, i.e., >h' as shown in Equation (67), then in another equation in Appendix A, there will be the creation of a new multisensory navigation map and updating it, rather than the modification of the existing **WNM**_*t*_]. The resulting multisensory navigation map **WNM'**_*t*_ is called the “Working Navigation Map” and is the navigation map upon which instinctive primitives and learned primitives operate in the Navigation Module (arrow “g,” Figure 2). Figure 3 is an example of a Working Navigation Map.


(67) $$\begin{array}{l} |\text{Object\_Seg\_Mod.differences}\left({\text{actual}}_{t},{\text{WNM}}_{t}\right)|\;\leq h\text{'},\\ \Rightarrow {\text{WNM'}}_{t}={\text{WNM}}_{t}\;\cup \;{\text{actual}}_{t}. \end{array}$$

### 2.2 The Navigation Module(s)

In the CCA5 version of the architecture, there is a single Navigation Module (Figure 1). However, the Navigation Modules are increased in the CCA6 version (Figure 5) and the CCA7 version (Figure 9). Nonetheless, this section applies to all these versions of the architecture.

Instinctive primitives are small procedures that can perform operations on the Working Navigation Map (**WNM'**_*t*_) in the Navigation Module. Instinctive primitives are pre-existing—they come preprogrammed with the architecture. Learned primitives are similar to small procedures that can perform operations on the Working Navigation Map (**WNM'**_*t*_). However, learned primitives are learned by the architecture, rather than being preprogrammed.

The instinctive primitives are inspired by the instinctive behaviors present in human infants and children, as well as in some non-human primates (Spelke, 1994; Kinzler and Spelke, 2007). Spelke groups these instinctive behaviors in terms of the physics of objects, agents, numbers, geometry, and reasoning about social group members.

As can be seen from Figure 2 (arrow “h”), the processed Input Sensory Vectors Association Modules' navigation maps, as well as inputs from other parts of the architecture, propagate to the store of both learned primitives and instinctive primitives in the architecture. Either an instinctive primitive or a learned primitive will be selected. The primitive is selected by a similar matching process to the one discussed above, but here in terms of the sensory inputs as well as signals from the Goal/Emotion Module and the previous values of the Navigation Modules.

Arrow “i” in Figure 2 shows the best-matching instinctive primitive acting on the Working Navigation Map (**WNM'**_*t*_) in the Navigation Module. These operations are essentially matrix/tensor operations, such as comparing arrays, adding a vector to an array, and other straightforward operations that could be expected of brain-inspired circuitry. Equation (82) is taken from Appendix A. In the Navigation Module, the best-matching primitive (it can be either an instinctive primitive or a learned primitive), **WPR**_*t*_ is applied against the Working Navigation Map **WNM'** (in the CCA6 and CCA7 versions of the architecture where there is more than one Navigation Module, this occurs in Navigation Module A).

In Equation (82), “apply_primitive” is a built-in method (i.e., part of the circuitry of the Navigation Module) that does the actual operations of applying the primitive **WPR**_*t*_ on the Working Navigation Map **WNM'**. The result is an output signal *action*_*t*_ (Equation 82). As indicated by arrow “j” in Figure 2, output signal *action*_*t*_ is propagated to the Output Vector Association Module. As indicated by arrow “k” in Figure 2, there is a modification of the output signal *action*_*t*_ with respect to temporal motions introduced in the Output Vector Association Module, creating an intermediate ***output_vector***_*t*_ signal. This is further modified by the ***motion_correction***_*t*_ signal produced by the Sequential/Error Correcting Module. The result is the ***output_vector***_*t*_ signal, as shown in Equation (87). The ***output_vector***_*t*_ signal propagates to the Output Vector Shaping Module. The signal is further transformed for interface with the real world (arrow “l,” Figure 2), where it can result in the activation of an actuator or the transmission of an electronic communication signal.


(82) $$\begin{array}{l} action_{t}=\text{Nav\_ModA.apply\_primitive}\left({\text{WPR}}_{t},{\text{WNM'}}_{t}\right) \end{array}$$

(87) $$\begin{array}{l} {\text{output\_vector'}}_{t}=\text{Output\_Vector\_Mod.apply\_motion}\\ \;\text{\_correction}\left({\text{output\_vector}}_{t},{\text{motion\_correction}}_{t}\right) \end{array}$$

Then, the cognitive cycle repeats—sensory inputs are processed, the Navigation Module may produce an output action which results in an actuator output, and then the cycle repeats again, and so on.

Step-by-step examples of the architecture processing a particular sensory scene are given by Schneider (2022a, 2023). In addition, more details on the processes that occur are given in Appendix A.

### 2.3 Feedback operations

Feedback pathways, only partially shown in Figures 1, 2, exist throughout the Causal Cognitive Architecture. These feedback pathways are essential—the architecture considers the differences with the actual input or intermediate signal compared to its existing internal representations, as discussed above. This is advantageous for the perception of noisy, imperfect sensory inputs.

**Figure 4:**
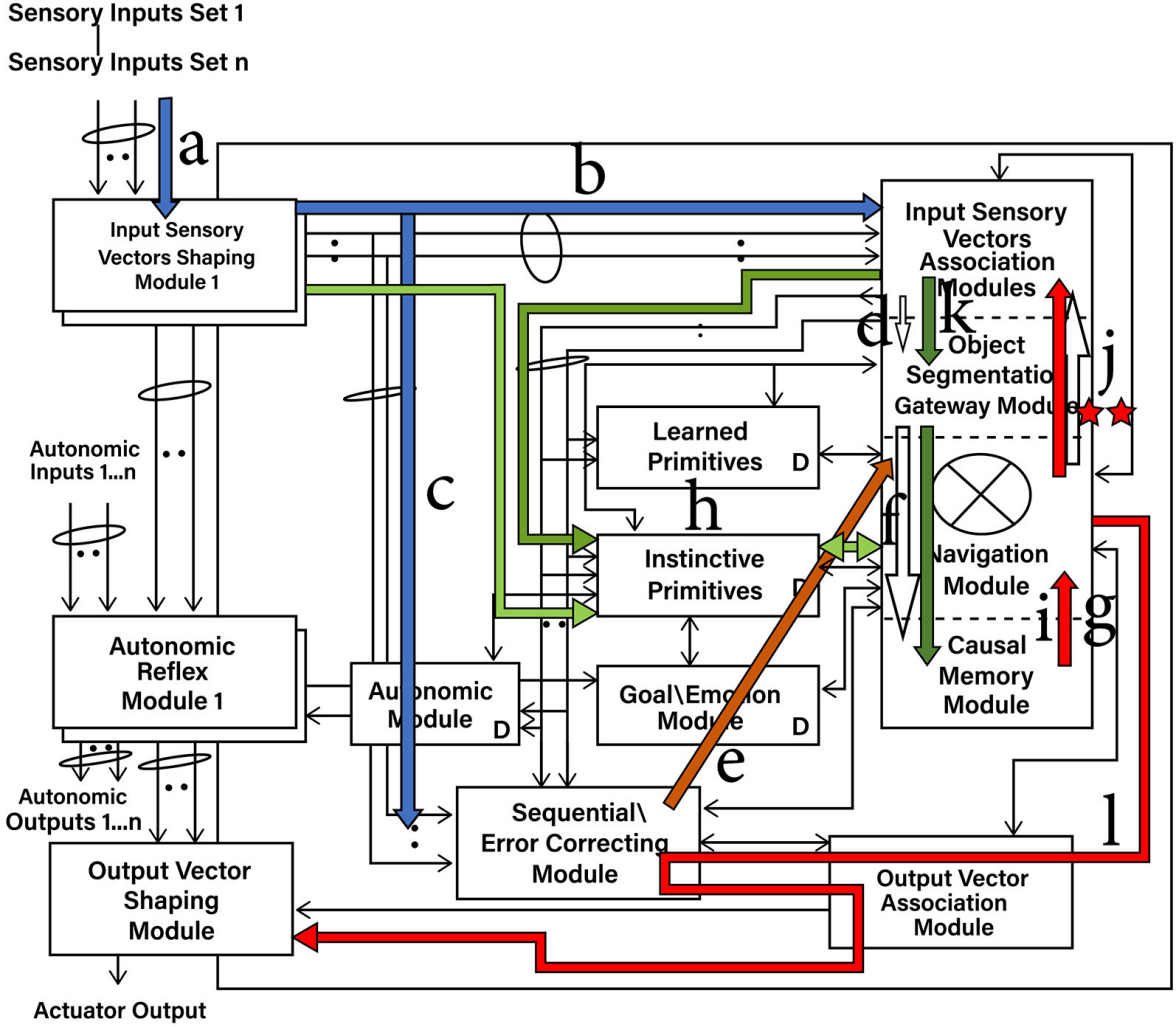
Consider the above figure under these situations: (A) *(arrows a*→*j)* When the operation of the selected instinctive or learned primitive on the Working Navigation Map in the Navigation Module does not produce an output action or a meaningful one, the results, i.e., the new contents of the Working Navigation Map, can instead be fed back to the Input Sensory Vectors Association Modules. (B) *(arrows k*→*l)* Instead of actual sensory inputs, the intermediate results from the Navigation Module (previous figure), which have been temporarily stored in the Input Sensory Vectors Association Modules, are now automatically propagated to the Navigation Module. In this new cognitive cycle, perhaps a new instinctive primitive will end up being selected (or the same ones used) and applied to previous intermediate results, possibly producing a valid output action. If so, then the output action goes to the Output Vector Association Module and then to the real world. However, if no valid output action occurs, the new intermediate results can be fed back again and, in the next cognitive cycle, processed again. (C) (j**) (Note: Although not shown, assume there is a TempMap memory storage area in the Navigation Module, which is shown more explicitly in Figure 5.) No meaningful output was produced by the interaction of the primitive on the Working Navigation Map (WNM) in the Navigation Module. As before, the Working Navigation Map (i.e., the intermediate results) are fed back and stored in the Sensory Association Modules. However, the operations at j** occur now (described below and in the text now occur). Effectively, induction by analogy automatically occurs in these steps, allowing the production of an actionable output in many situations. a—Sensory inputs stream into the Input Sensory Vectors Shaping Modules. b—Processed and normalized sensory inputs propagate to the Input Sensory Vectors Association Modules and best-matching Local Navigation Maps (LMNs) for each sensory system produced (spatial binding). c—Processed and normalized sensory inputs propagate to the Sequential/Error Correcting Module (temporal binding). d—Segmentation of objects in the input sensory scenes. e—Spatial mapping of the temporal mapping of the sensory inputs from the Sequential/Error Correcting Module. f—Match to the best-matching Multi-Sensory Navigation Map from the Causal Memory Module, producing the Working Navigation Map (WNM) for the Navigation Module. g—Working Navigation Map (WNM) in/accessible by Navigation Module. h—Selecting a best-matching primitive (an Instinctive Primitive in this case). i—Instinctive Primitive operating on the Working Navigation Map (WNM). j—No meaningful *action* signal is produced by the operation of Instinctive Primitive on WNM; thus, the contents of WNM are fed back to the Input Sensory Vectors Associations Modules. k—In the new cognitive cycle, the stored WNM, i.e., the previous cycle's intermediate results, are now reprocessed through the Navigation Module. l—Perhaps a meaningful action signal is now produced, and an output signal results. j**–No meaningful *action* signal is produced by the operation of Instinctive Primitive on WNM; thus, the following happens (represented above by ** since there is not enough space to show the various arrows required): 1. The contents of WNM are fed back to the Input Sensory Vectors Associations Modules, like before. 2. The contents of WNM are also fed to trigger a match with the best-matching nav map in the Causal Memory Module (**WNM'**_*t*_-*best_match*). 3. The nav map that **WNM'**_*t*_-*best_match* links to is placed in “**TempMap**” memory of the Navigation Module. 4. The difference (**WNM'**_*t*_-*difference*) between **WNM'**_*t*_-*best_match* and “**TempMap**” is kept in the Navigation Module. 5. The original **WNM** being stored in the Input Sensory Vectors Association Modules in the next cognitive cycle propagates forward and is added to **WNM'**_*t*_-*difference*, resulting in a new Working Navigation Map **WNM**, which will be processed again (i.e., action by primitive against it) in the new cognitive cycle, and this time may (or may not) result in an actionable output from the Navigation Map.

Schneider (2022a) describes how, by enhancing some of these feedback pathways, causal properties readily emerge from the architecture. In Figure 4A, the feedback pathways from the Navigation Module back to the Input Sensory Vectors Association Modules are enhanced. Biologically, such a change could have occurred in the evolution of the brain through a number of genetic mechanisms (e.g., Rakic, 2009; Chakraborty and Jarvis, 2015).

The result of this enhancement of this feedback pathway is that when the operation of the instinctive or learned primitive on the Working Navigation Map in the Navigation Module does not produce an output action or a meaningful output action, the results, i.e., the new contents of the Working Navigation Map, can instead be fed back to the Input Sensory Vectors Association Modules instead of being sent to the Output Vector Association Module.

As shown in Figure 4A, the Navigation Module in this cognitive cycle did not produce any output signal *action*_*t*_. However, as arrow “j” (Figure 4A) shows, the contents of the Navigation Module are fed back to the Input Vectors Association Module. These saved contents essentially now represent the intermediate results of the Navigation Module. In the next cognitive cycle, they can be fed forward to the Navigation Module and operated on again.

As Figure 4B shows, in the next cognitive cycle, instead of processing the actual sensory inputs, these stored intermediate results will automatically propagate forward to the Navigation Module and be processed again (arrow “k”). These saved, essentially intermediate results become again the current Working Navigation Map (**WNM'**) in the Navigation Module. The advantage of reprocessing intermediate results is that another operation of the instinctive (or learned) primitive on these results can often result in a better, actionable output signal *action*_*t*_ (Equation 82). If not, the new intermediate results (i.e., the new contents of the Working Navigation Map) can be fed back and again be re-processed in the next one or many repeated cognitive cycles (at present, determination of what is a meaningful result can be determined in part by a learned or instinctive primitive's procedures, as well as in part if the action signal sent to the Output Vector Association Module can be acted upon).

Equations (88, 89) taken from Appendix A show that if the action signal produced by the Navigation Module is not actionable (i.e., *action*_*t*_ ≠ “move^*^”), then the Working Navigation Map in the Navigation Module is fed back to the various sensory system Input Vector Association Modules (Equation 88). In the next cognitive cycle t = t+1, the best-matching Local Navigation Map in each module now becomes the sensory features extracted from the fed-back Working Navigation Map (Equation 89), and so, these Local Navigation Maps will be automatically propagated forward to the Navigation Module, where they will constitute the Working Navigation Map again. Thus, intermediate results of the previous cycle will be operated on a second time by whatever instinctive or learned primitives are selected in this cognitive cycle.

While this seems like an inelegant way to re-operate on intermediate results, evolving this mechanism in the brain takes modest changes, i.e., enhancement of particular feedback pathways. Indeed, in humans, when the brain switches from the automatic operations of System 1 to the more complex operations of System 2 (Kahneman, 2011), which is similar to re-operating on intermediate results, less attention can be paid to the normal stimuli around us, which is what happens in the Causal Cognitive Architecture during re-operating on intermediate results.


(88) $$\begin{array}{l} \;\left(action_{t}\neq “move^{*}\text{” }and\;{\text{WPR}}_{t}\neq \left[“discard^{*}\text{”}\right]\right)or\\ \;{\text{WPR}}_{\text{t}}=\left[“feedback^{*}\text{”}\right],\\ \{\Rightarrow \text{Nav\_ModA.feedback\_to\_assocn\_mod}\left({\text{WNM'}}_{t}\right) \end{array}$$

(89) $$\begin{array}{l} \Rightarrow \forall_{\sigma :}{\text{LNM}}_{\left(\sigma ,\gamma ,t+1\right)}=\text{Input\_Sens\_Vectors\_}\\ {\text{Assoc\_Module}}_{\sigma }.\text{extract}\text{\_}\sigma ({\text{WNM'}}_{t})\} \end{array}$$

Schneider (2022a) shows that by re-operating on the intermediate results, the architecture can generate casual behavior by exploring possible causes and effects of the actions. An example is where the CCA3 version of the Causal Cognitive Architecture controls a hospital patient assistant robot. A new robot has never seen a patient fall to the ground and has never been trained on a closely identical case. However, it has a learned primitive (i.e., procedure) from a rudimentary education before doing this work that it should not allow any patient it is with to fall down on the ground.

The robot one day happens to be assisting a patient who begins to fall toward the ground. The learned primitive concerning a person falling is triggered but does not produce an actual output signal. The intermediate result calls the physics instinctive primitive (i.e., a general knowledge procedure pre-programmed in the architecture), which pushes back against something falling or moving to stop the movement. Thus, the robot pushes back against the falling patient and stops the patient from falling, even though it has never actually done this before in training.

### 2.4 Analogical reasoning

Even with reprocessing of intermediate results, there are still many situations where the Navigation Module will not produce any actionable output. Schneider (2023) shows that with biologically feasible, modest modifications to the feedback operations, analogical reasoning readily emerges. Although not explicitly shown, note that a temporary memory area, “**TempMap**,” now also exists in the Navigation Module (Figure 4C; this memory area is treated equivalently to an array in the equations, hence the bolding of its name).

Given the existence of a temporary memory area, why, for causal behavior, as shown above, is it necessary to feed back the intermediate results of the Navigation Module to the Input Sensory Vectors Association Modules rather than just store them in the temporary memory area? As Schneider (2023) notes, the reason is that the Causal Cognitive Architecture is biologically inspired, and from an evolutionary perspective, it seems more reasonable that storage of intermediate results could occur by enhancing feedback pathways rather than by creating new memory areas. To efficiently carry out analogical operations, as described below, the evolutionary usurpation of some brain regions as a temporary storage region would have been advantageous at this point. Thus, the CCA5 and higher versions of the architecture possess a temporary memory area associated with the Navigation Module.

As before, the Working Navigation Map (**WNM'**_*t*_; i.e., the intermediate results) is fed back and stored in the Input Sensory Association Modules (arrow “j” in Figures 4B, C). However, the Working Navigation Map is also propagated to the Causal Memory Module, where it will automatically match the best stored multisensory navigation map, which becomes the new Working Navigation Map in the Navigation Module. The navigation map that this navigation map recently linked to is triggered and retrieved and then propagated to and subtracted from the new Working Navigation Map in the Navigation Module (“^**^” in Figure 4C). In the next cognitive cycle, as before, the original Working Navigation Map is automatically propagated, although now added to the differences in the Navigation Module. As a result of a few modifications to the feedback pathways and operations on the navigation maps here, effectively induction by analogy automatically occurs in these steps.

Equations (95–99) are taken from the Appendix A and show these operations. If there is no actionable output from the Navigation Module (i.e., *action*_*t*_ ≠” move^*^,” where “move^*^” is an output signal giving instructions about moving something or moving a message), then these operations are automatically triggered, i.e., these equations occur [note that there is only one Working Navigation Map **WNM'**_*t*_ in the Navigation Module (Figure 4C) at any time. However, since the contents of what is **WNM'**_*t*_ change several times in these operations, for better clarity to the reader, a small descriptor is appended to its name, e.g., “**WNM'**_*t*_-*original*,” etc.].

In Equation (95), the contents of the Navigation Module, i.e., **WNM'**_*t*_, which for better readability is called “**WNM'**_*t*_-*original*” here, are fed back to be stored in the Input Sensory Vectors Association Modules, the same as before. However, “**WNM'**_*t*_-*original*” is also propagated to the Causal Memory Module where automatically the “Causal_Mem_Mod.match_best_map” built-in method will occur, matching it with the best matching multisensory map, which becomes the new Working Navigation Map called here as “**WNM'**_*t*_-*best_match*” (Equation 96).

In Equation (97), the built-in method “Nav_ModA.use_linkaddress1_map” retrieves the navigation map that “**WNM'**_*t*_-*best_match*” most recently linked to and puts this map into the **TempMap** memory. Then, as Equation (98) shows, the difference between “**WNM'**_*t*_-*best_match*” and **TempMap** gets stored in the Navigation Module as “**WNM'**_*t*_-*difference*.” Then, a new cognitive cycle starts, and **WNM'**_*t*_-*original* is automatically fed forward but now added to “**WNM'**_*t*_-*difference*” (Equation 99). The new Working Navigation Map **WNM'**_*t*_ is termed “**WNM'**_*t*_-*analogical*” since it represents an analogic inductive result.

Consider a navigation map **x** which is represented by “**WNM'**_*t*_-*original*.” Given that there was no actionable output in the last cognitive cycle, it is advantageous to induce what this navigation map **x** will do next, i.e., which navigation map it will call. In Equation (100), it is shown that it has properties/features P_1_…P_n_. Consider navigation map **y** represented as “**WNM'**_*t*_-*best_match*” in Equation (96). It is the best-matching navigation map to navigation map **x** and thus assumed it will share many properties, as shown in Equation (101).

Navigation map **y** has previously linked to (i.e., it was pulled into the Navigation Module) the navigation map in **TempMap** (Equation 97), i.e., as given by the built-in method “use_linkaddress1_map” [Schneider (2023) discusses other links and groups of links to use as the basis for analogical induction]. The difference between navigation map **y** and **TempMap** is “**WNM'**_*t*_-*difference*” (Equation 98). Thus, consider this difference, i.e., “**WNM'**_*t*_-*difference*,” to be property N, as noted in Equation (102).

Since navigation map **y** has property N, by induction by analogy, it can be said that navigation map **x** also has property N (Equation 103). Thus, add property N, which is actually “**WNM'**_*t*_-*difference*,” to navigation map **x**, which is actually “**WNM'**_*t*_-*original*,” producing the result of navigation map **x** with property N as being “**WNM'**_*t*_-*analogical*” (Equation 99).


(95) $$\begin{array}{l} \left((action_{t}\neq “move^{*}\text{” or }{\text{WPR}}_{t}=\left[“analogical^{*}\text{”}\right]\right)\;\text{and }{\text{WPR}}_{t}\\ \neq [“discard^{*}\text{”}]\;\text{and }{\text{WPR}}_{t}\neq \;[“feedback^{*}\text{”}]),\\ \{\Rightarrow \text{Nav\_ModA.feedback\_to\_assocn\_mod}\\ \;({\text{WNM'}}_{t}-original)\\ \Rightarrow {\text{WNM'}}_{t}-best\text{\_}match \end{array}$$

(96) $$\begin{array}{c} =\text{Causal\_Mem\_Mod.match\_best\_map}\left({\text{WNM'}}_{t}-original\right) \end{array}$$

(97) $$\begin{array}{l} \Rightarrow {\text{TempMap}}_{t}=\text{Nav\_ModA.use\_linkaddress1\_map}\\ \;\left({\text{WNM'}}_{t}-best\text{\_}match\right) \end{array}$$

(98) $$\begin{array}{l} \Rightarrow {\text{WNM'}}_{t}-difference=\text{Nav\_ModA.subtract}\\ \;({\text{WNM'}}_{t}-best\text{\_}match_{,}{\text{TempMap}}_{t})\} \end{array}$$

(99) $$\begin{array}{l} \;((action_{t-1}\neq “move^{*}\text{” or }{\text{WPR}}_{\text{t}-1}\\ \;=[“analogical^{*}\text{”}])\;\text{and }{\text{WPR}}_{\text{t}-1}\\ \neq [“discard^{*}\text{”}]\;\text{and }{\text{WPR}}_{\text{t}-1}\neq \;[“feedback^{*}\text{”}]),\\ \Rightarrow {\text{WNM'}}_{t}-analogical\\ =\text{Nav\_ModA.retrieve\_and\_add\_vector\_assocn()} \end{array}$$

(100) $$\begin{array}{l} P_{1}\text{x \&}\;P_{2}\text{x \& …}\;P_{\text{n}}\text{x} \end{array}$$

(101) $$\begin{array}{l} P_{1}\text{y \&}\;P_{2}\text{y \& …}\;P_{\text{n}}\text{y} \end{array}$$

(102) $$\begin{array}{l} \;N\text{y} \end{array}$$

(103) $$\begin{array}{l} ∴N\text{x} \end{array}$$

As noted above, Equations (100–103) essentially define induction by analogy. In Equation (100), **x** has properties/features P_1_ to P_n_. **y** is similar and also has properties/features P_1_ to P_n_ (Equation 101). **y** also has the feature “N” (Equation 102). Thus, by induction by analogy, **x** has the feature “N” (Equation 103). As shown above, a ready mechanism now exists in the Causal Cognitive Architecture, which follows this definition. If an actionable resolution of a Working Navigation Map (**WNM'**_*t*_) does not immediately occur (i.e., a primitive applied to **WNM'**_*t*_ does not produce an actionable output from the Navigation Module), the architecture can follow the analogical mechanism above to produce an analogical result which can be operated on in the next cognitive cycle.

Of interest is that analogical intermediate results are useful in typical day-to-day functioning rather than being considered as something only used in exceptional high-level problem-solving tasks (e.g., writing an intelligence test). For example, in the study by Schneider (2023), there is an example of a robot controlled by a CCA5 version of the architecture. The robot needs to cross a river and has instinctive primitives that guide it to stay on solid ground to do so. However, there are piles of leaves floating on the river, which appear solid and for which the robot has no experience nor any instinctive primitives. By analogical reasoning, it is shown how the robot automatically uses a previous navigation map (i.e., experience) of stepping on pieces of newspaper floating in a puddle and its leg going into the puddle to not use the leaves to cross the river. The robot has no knowledge whatsoever about newspapers or leaves other than they appear to be solid, yet by automatically using its analogical reasoning mechanism, it successfully crosses the river via another path and not by stepping on the piles of floating leaves.

Hofstadter (2001) provides evidence that supports the use of analogies as the core of human cognition. Of interest, full analogical reasoning does not appear to be present in chimpanzees (Penn et al., 2008), although more recent reports show some animals capable of some aspects of analogical reasoning (Flemming et al., 2013; Hagmann and Cook, 2015). The mechanisms described above for the Causal Cognitive Architecture show theoretically that modest modifications can result in the emergence of analogical reasoning from a chimpanzee–human last common ancestor, albeit a loosely functionalist model thereof.

### 2.5 Compositionality

**Figure 5:**
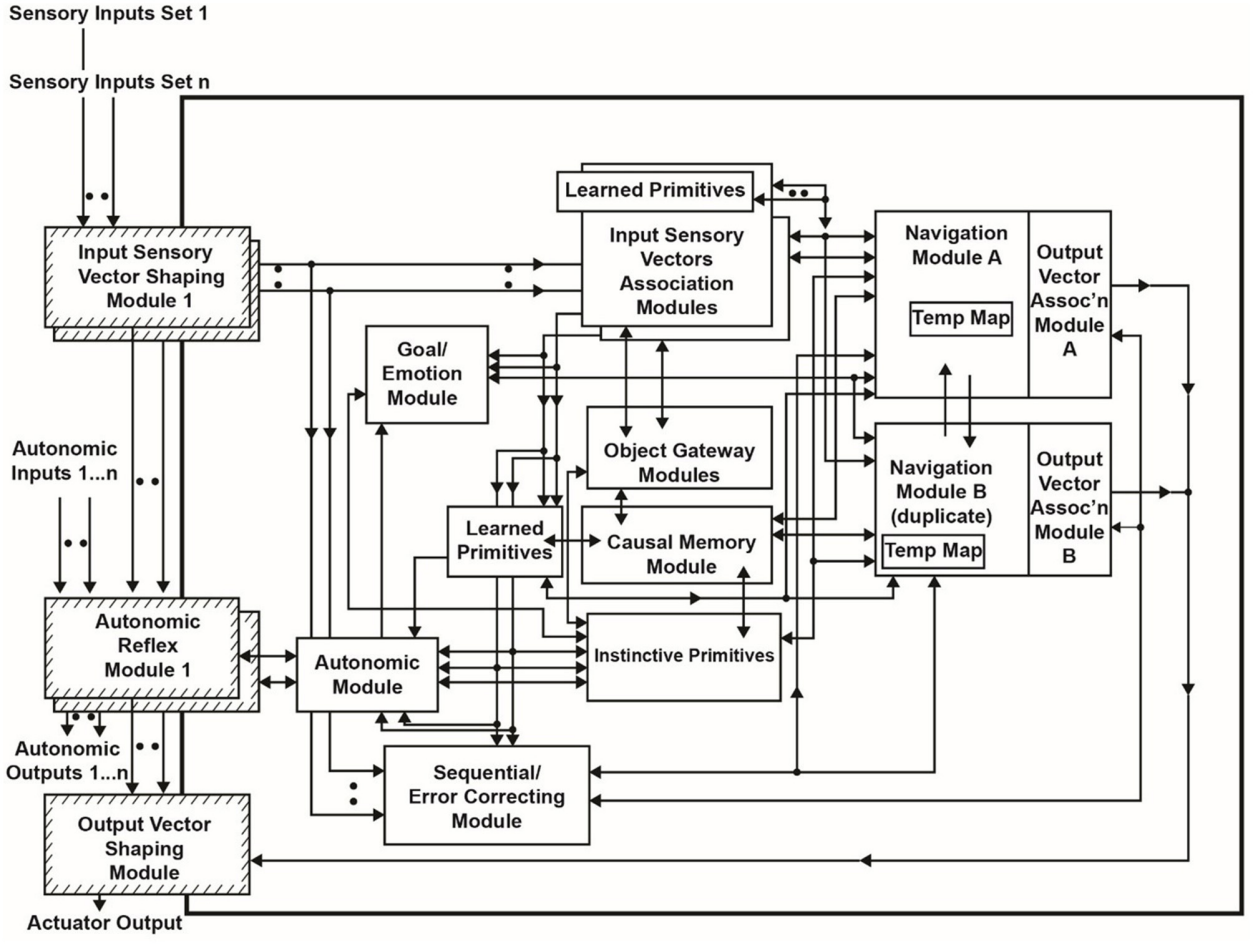
The Causal Cognitive Architecture 6 (CCA6). The Navigation Module of the CCA5 architecture has been duplicated into Navigation Module A and Navigation Module B, along with other changes, resulting in the CCA6 version of the architecture.

Given the usefulness of the Navigation Module, duplicating it into two Navigation Modules might be more advantageous. Again, biologically, such a change could have readily occurred in the evolution of the brain through a number of genetic mechanisms (e.g., Rakic, 2009; Chakraborty and Jarvis, 2015). Figure 5 shows the duplication of the Navigation Module into Navigation Module A and Navigation Module B. This new version of the architecture is called the Causal Cognitive Architecture 6 (CCA6).

**Figure 6:**
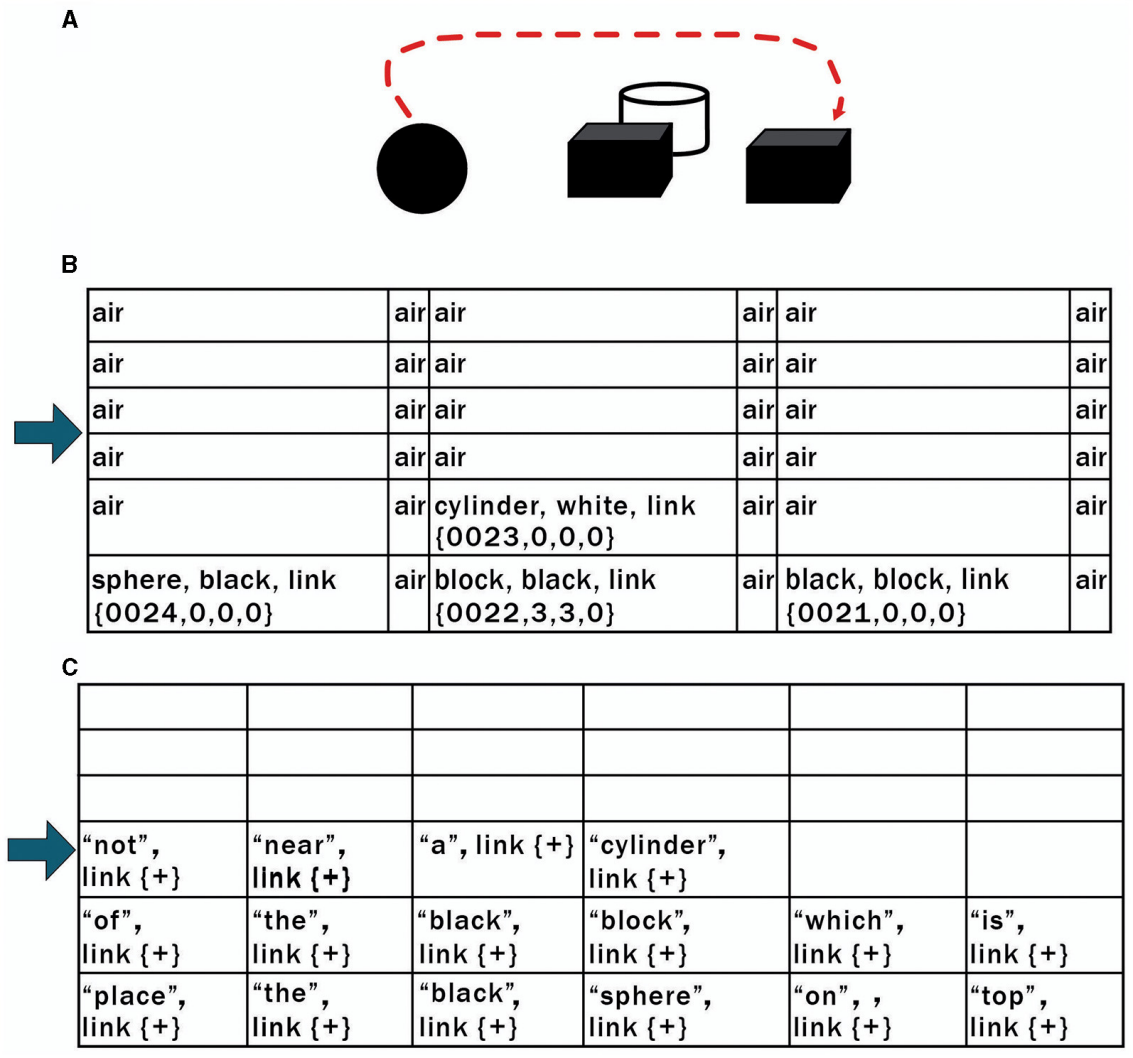
**(A)** Connectionist systems have difficulty solving problems such as “place the black sphere on top of the black block which is not near a cylinder” (*the arrow shows the correct solution to this problem, but of course, it is not shown to the system being asked to solve this problem)*. **(B, C)** The sensory scene of **(A)** is loaded in Navigation Module A **(B)**. The instruction associated with **(A)** *(“place the black sphere on top of the black block which is not near a cylinder”)* is loaded in Navigation Module B **(C)**. In subsequent cognitive cycles, the contents of Navigation Module B are processed against the contents of Navigation Module A via operations of various instinctive primitives discussed in the text.

Consider the compositional problem shown in Figure 6A, such as following the command to “place the black sphere on top of the black block which is not near a cylinder” (the arrow shows the correct solution to this problem. Of course, the arrow is not shown to the system being asked to solve this problem). Connectionist systems have trouble with such compositional problems. For example, Marcus et al. (2022) give a similar example to DALL-E2 and prompt it to place a red ball on top of a blue pyramid behind a car above a toaster. DALL-E2 tries 10 times and produces various output images in response to the command, but none of these actually depict the requested relationships correctly.

However, Schneider (2024) shows that if the Navigation Module is duplicated into Navigation Module A and Navigation Module B, as shown in Figure 5, then compositionality and compositional language readily emerge from this CCA6 version of the architecture.

In the CCA6 architecture shown in Figure 5, compositional operations tend to occur in Navigation Module B. Instinctive primitives (as well as learned primitives) involved in compositional operations and language operations will generally operate on the navigation map in Navigation Module B. Consider the example shown in Figure 6A of “placing the black sphere on top of the black block which is not near a cylinder.” The sensory scene of the spheres and blocks will propagate through the architecture (Figure 5) and be mapped to a navigation map in Navigation Module A, as shown in Figure 6B [it actually takes a few cognitive cycles and close-up views of the objects, as evidenced by the links in some of the cells (e.g., link to {map = 24, x = 0, y = 0, z = 0} for cell (0,0,0) with the labels “sphere” and “black”), to create this navigation map].

Equations (109–114) are taken from Appendix A. The instinctive primitive “parse_sentence” is triggered by the instruction (“*instruction sentence*”) associated with this sensory image. In Equation (109), “parse_sentence.copy()” maps the instruction (“place the black sphere on top of the black block which is not near a cylinder”) to a navigation map (**WNMB'**_*t*_) in Navigation Module B (“Nav_ModB”). This is shown in Figures 6B, C. The “link{+}” in the cells in the Navigation Map (**WNMB'**_*t*_) in Navigation Module B just means that the cell links to its neighbor to the right.

**Figure 7:**
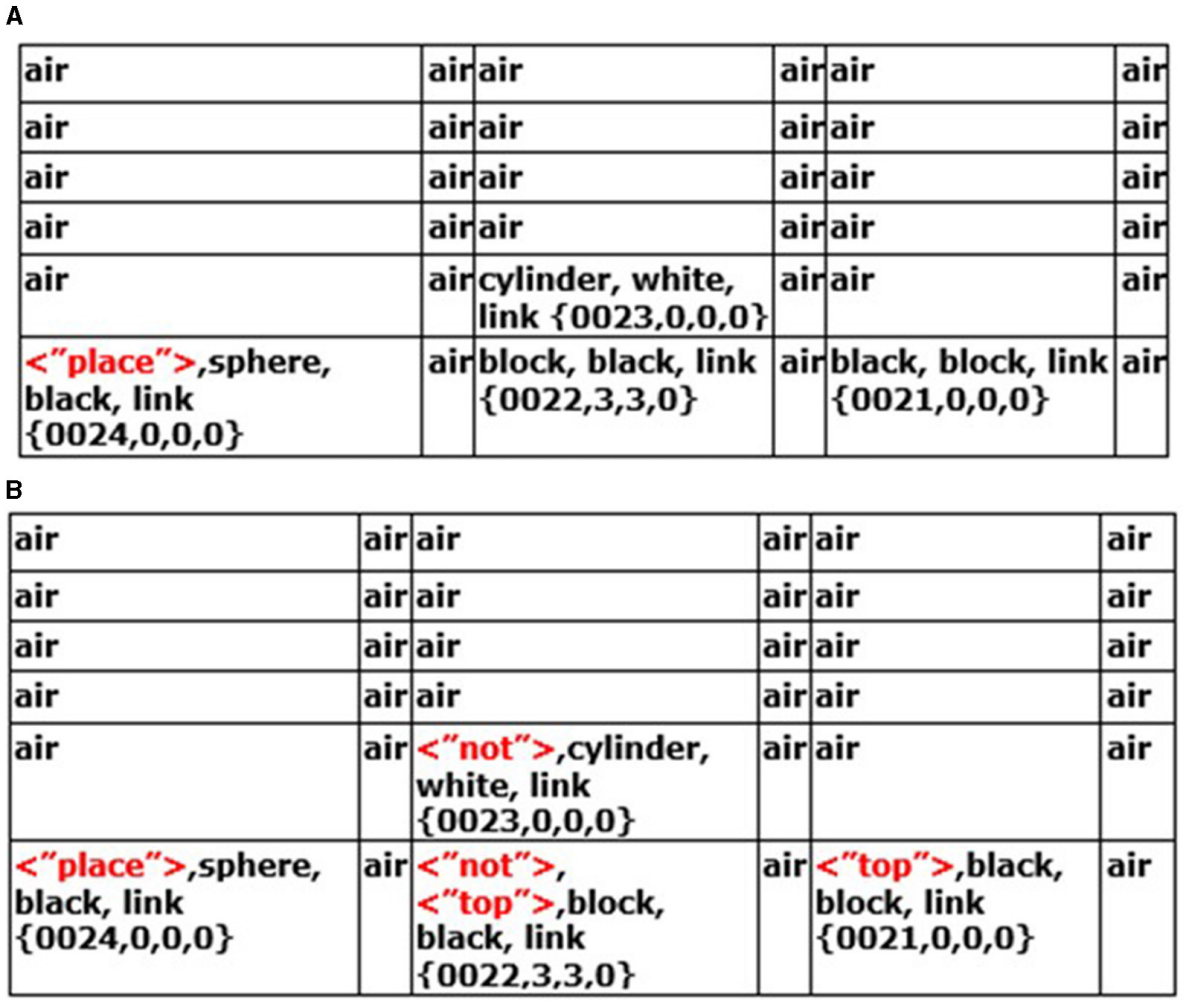
**(A)** The instinctive primitive “parse_sentence()” has entered the tag < “place”> in the cell containing the black sphere. **(B)** After a few more cognitive cycles, the instinctive primitive “parse_sentence()” and then the instinctive primitive “physics_near_object()” have now written these tags in the various cells of the navigation map in Navigation Module A.

The “parse_sentence.parse()” instinctive primitive parses through Navigation Map B **WNMB'**, i.e., the instruction sentence (Equation 110). Each word is matched against the Causal Memory Module “parse_sentence.parse.match()” (Equation 111). If what is called an “action word” is found (i.e., it matches some specific action to do to the other cells), then it is mapped to cells in Navigation Map A **WNM'** in Navigation Module A (Figure 7A) containing features associated with or mapping to the action word. In Figure 7A, it can be seen, for example, that “place” has been matched to cell (0,0,0) in Navigation Module A. This makes sense since this action word is associated with the black sphere in the instruction sentence (this is described in more detail in Appendix A).

A “*near_trigger*” is a feature that is spatially near something else or not near something else that can trigger various physics instinctive primitives. The instruction sentence word “near” triggers instinctive primitive “Nav_ModB.physics_near_object()” (Equation 112). The result of this instinctive primitive is to place the tag “not” in the cells “not near” the white cylinder, as seen in the Navigation Map of Navigation Module A in Figure 7B.

In each cognitive cycle, the CCA6 architecture will continue to parse through the instruction sentence. When it reaches the “*end_of_communication*” (i.e., the end of the sentence), it then parses through Navigation Module A, looking for a “place” tag. Suppose there is a “place” tag (e.g., cell (0,0,0)) in Navigation Module A in Figure 7B, then instinctive primitive “Nav_ModA.place_object()” is triggered (Equation 113). This instinctive primitive will go through the navigation map looking for other tagged notations such as “top” in cells (2,0,0) and (4,0,0) in Navigation Module A (Figure 7B). It will ignore (2,0,0) since there is a “not” tag there, but it will consider (4,0,0) valid. It will then trigger the instinctive primitive “Nav_ModA.move(),” which then sends the action signals to the Output Vector Association Module A, which in turn sends a motion-corrected signal to the Output Vector Shaping Module, which instructs the actuators to move the black sphere to the cell (4,0,0) with the black block on the right (Equation 114).


(109) $$\begin{array}{l} \;\left(instruction\text{\_}sentence\right),\\ \{\Rightarrow {\text{WNMB'}}_{t}=\text{Nav\_ModB.parse\_sentence.copy()}\; \end{array}$$

(110) $$\begin{array}{l} \Rightarrow \text{Nav\_ModB.parse\_sentence.parse}(\text{WNMB'}{\;}_{t}), \end{array}$$

(111) $$\begin{array}{l} {}[\Rightarrow \text{Nav\_ModB.parse\_sentence.parse.match()}\; \end{array}$$

(112) $$\begin{array}{l} \;\Rightarrow \text{near\_trigger},\\ \left(\Rightarrow \text{Nav\_ModB.physics\_near\_object()})\right] \end{array}$$

(113) $$\begin{array}{l} \;\Rightarrow \text{end\_of\_communication},\\ \;[<\text{place}>,\\ (\Rightarrow \text{Nav\_ModA.place\_object()} \end{array}$$

(114) $$\begin{array}{l} \Rightarrow \text{Nav\_ModA.move()})]\} \end{array}$$

Compositionality is a key property of an intelligent system. Without compositionality, such a system would need to experience every (or very many) permutations of a vast number of sensory scenes and actions to learn them. Above, it was shown how, with the duplication of the navigation modules, compositional abilities can readily emerge. This is discussed in more detail in Schneider (2024), including the emergence of language.

### 2.6 Comparison of the Causal Cognitive Architecture with other cognitive architectures

Samsonovich (2010) and Kotseruba and Tsotsos (2020) review the many cognitive architectures proposed in the literature. Kotseruba and Tsotsos note the large diversity of cognitive architectures proposed and the difficulty of defining the term. They consider cognitive architectures broadly as programs that “could reason about problems across different domains” and attempt to help determine what “particular mechanisms succeed in producing intelligent behavior” in terms of modeling the human mind.

Laird et al. (2017) attempt to unify the field of cognitive architectures with what they term a “standard model of the mind.” In their standard model, perception feeds into working memory, while motor outputs feed out of it. There is bidirectional movement of information between a declarative long-term memory and the working memory. Similarly, there is a bidirectional movement of information between procedural long-term memory and working memory. This is a very generic model of a cognitive architecture, and it would be expected to capture the inclusion of most of the models listed by Samsonovich (2010) or Kotseruba and Tsotsos (2020). However, the CCA7 version of the Causal Cognitive Architecture surprisingly does not fit within this “standard model.”

In this standard model of the mind, there are separate areas for declarative long-term memory and procedural long-term memory. However, in the CCA7, there can be both declarative long-term memory (i.e., navigation maps of experiences) and procedural long-term memory (i.e., instinctive and learned primitives) mixed together in the different Input Sensory Vectors Association Modules and within multisensory navigation maps which are operated on in the Navigation Modules A and B.

The CCA7 architecture is largely defined by its binding of sensory inputs into navigation maps and comparing these inputs with pre-stored information. The CCA7 architecture is also largely defined by its heavy usage of feedback of intermediate results of navigation maps. Again, these operations are not typical for most of the architectures defined by the standard model of the mind or included by Samsonovich (2010) or by Kotseruba and Tsotsos (2020).

### 2.7 Cognitive maps

As noted above, the Causal Cognitive Architecture hypothesizes that the navigation circuits in the amniotic ancestors of mammals duplicated many times to eventually form the neocortex. Thus, the millions of neocortical minicolumns are modeled in the Causal Cognitive Architecture as millions of navigation maps. As noted above, using this postulation, it has been possible to show the emergence of causal reasoning, analogical reasoning, and compositionality from a brain based on such navigation maps [Schneider, 2022a, 2023, 2024; Albeit, not rigidly replicating the mammalian brain, but at a more functionalist system as per Lieto (2021b)].

Similar to the concept of navigation maps, cognitive maps were proposed by Tolman (1948). A cognitive map is considered the way the brain represents the world and allows navigation and operations on paths and objects in the world. Thus, cognitive maps can hold geographical information as well as information from personal experiences. Before the work by O'Keefe and Nadel (1978), there was much debate about the existence of cognitive maps in a large spectrum of the animal world. This debate still continues, for example, whether cognitive maps exist in insects (Dhein, 2023).

In mammals, experimental work has largely found evidence for cognitive maps existing in terms of spatial navigation (e.g., O'Keefe and Nadel, 1978; Alme et al., 2014). However, Behrens et al. (2018) and Whittington et al. (2022) review extensions of cognitive maps into other domains of cognition. Hawkins et al. (2019) note evidence within the mammalian neocortex for the equivalent of grid cells. Schafer and Schiller (2018) have also hypothesized that the mammalian neocortex contains maps of spatial objects and maps of social interactions.

Buzsaki and Moser (2013) consider cognitive maps in planning, an area in which the new work on the Causal Cognitive Architecture below has developed. They propose that the memory and planning properties of the mammalian brain actually evolved from the same mechanisms used for navigation of the physical world. With regard to the neuroanatomical and neurophysiological basis for cognitive maps in the brain, the study by Schuck et al. (2016) suggests that the human orbitofrontal cortex holds a cognitive map of the current states of a task being performed.

## 3 New work: the Causal Cognitive Architecture 7 (CCA7)

### 3.1 Duplication of the TempMap memory areas

As noted in the Introduction section, Causal Cognitive Architecture is a brain-inspired cognitive architecture (BICA) that was developed from the hypothesis that the navigation circuits in the amniotic ancestors of mammals duplicated many times to eventually form the neocortex. The thousands or millions (depending on the organism) of neocortical minicolumns are modeled in the architecture as navigation maps. The modeling of the mammalian brain and its evolution is done in a loosely functionalist approach (e.g., Lieto, 2021b) with constraints imposed by structuralist concerns. Small modifications in the architecture, akin to what could have been reasonable genetic and developmental changes, have been postulated and explored in the development of the versions of the architecture from the Causal Cognitive Architecture CCA1 version to the CCA6 version.

This very functionalist and theoretical approach to mammalian brain functioning and evolution is quite different than approaches that have attempted to more faithfully replicate brain structure and function (e.g., Markram, 2012; Frégnac, 2023). However, the approach taken by the Causal Cognitive Architecture does allow the emergence of mechanisms that could hypothetically explain the functioning of the mammalian brain as well as how ordinary genetic and developmental mechanisms could have readily allowed the emergence, i.e., evolution, of the seemingly discontinuous features in humans (i.e., the sharp cognitive and behavioral differences between humans and our closest evolutionary relatives). In addition, the approach taken by the Causal Cognitive Architecture creates a mechanism (i.e., a particular cognitive architecture) that can be used as the basis of building intelligent artificial systems.

As noted above, in this study, the question is asked what if the evolution of the human brain were to continue as it has in the past, and given an environment selecting for the ability to better solve complex problems which humans encounter in their lives (very roughly indicated by measures of intelligence, for example, e.g., Legg and Hutter, 2007; Adams et al., 2012; Wang, 2019), then what advantageous changes could occur as reflected in a model such as the Causal Cognitive Architecture?

A computer engineer interested in enhancing the “intelligence” (as per the definitions above) capabilities of the CCA6 version of the architecture (Figure 5) could readily add a large language model (LLM) module to the architecture or even just add a simple calculator module to the architecture. If one assumes that the CCA6 could be developed to the point of human intelligence (i.e., with adequate instinctive and learned primitives and with adequate experiences stored throughout the architecture), then adding even a calculator module could create a super-human intelligence (albeit, given the assumptions above). For example, in computing various strategies or outcomes, numerical answers would be readily available for many problems in such an architecture, unlike in the CCA6 version shown in Figure 5 or unlike in the case of an actual human.

Adding a calculator module or, even more advantageously, adding complete or multiple LLM modules to the Causal Cognitive Architecture in Figure 5 could be considered in future work. Indeed, adding LLMs to cognitive architectures is an active area of research at the time of writing (e.g., Joshi and Ustun, 2023; Laird et al., 2023; Sun, 2024). However, in this study, the assumption is that there will be an environment selecting for the ability to better solve complex problems. Thus, although there is not in this study the construction, mutation, and testing of millions of copies of the CCA6, there is a consideration of what advantageous modifications could emerge next, rather than design in modules (e.g., calculator module, LLM, and so on), which would not emerge naturally on their own as such (a calculator module or a complex LLM module would not readily emerge on its own from the CCA6 version of the architecture shown in Figure 5).

**Figure 8:**
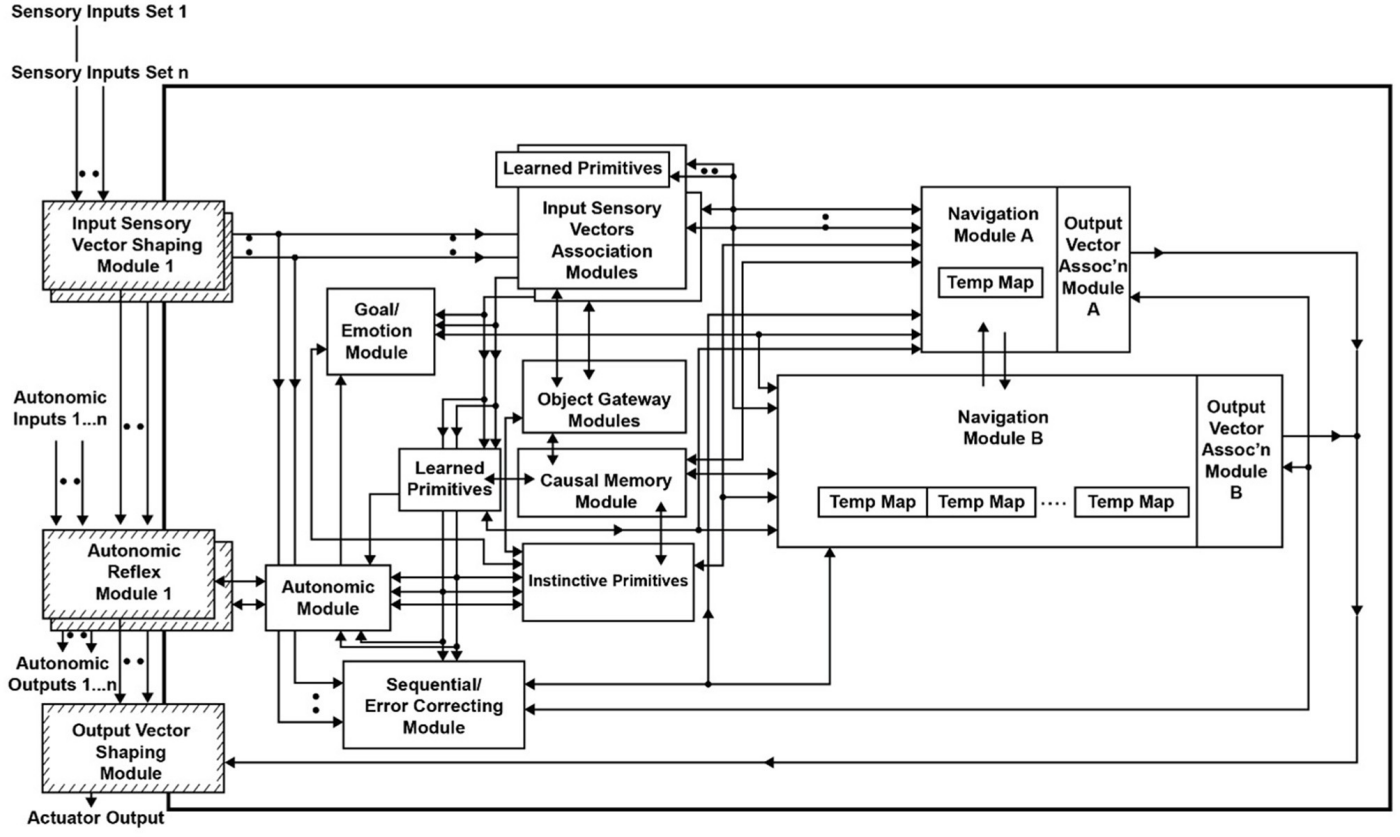
The CCA6 architecture with the duplication of the **TempMap** temporary memory areas in Navigation Module B.

It is hypothesized that a first step in such an evolution could be the duplication of the **TempMap** temporary memory area in Navigation Module B into many such **TempMap** temporary memory areas. As noted above, various mechanisms are feasible for the duplication of brain structures (e.g., Rakic, 2009; Chakraborty and Jarvis, 2015). Thus, as a first step in the continued evolution of the CCA6 version of the architecture, there are multiple duplications of the **TempMap** temporary memories in Navigation Module B. This is shown in Figure 8. Previously, there was a single **TempMap** temporary memory area in Navigation Module B; now, there are many.

The temporary memory area **TempMap** was discussed above in its use for allowing analogical reasoning (Equation 97). Mammalian brain working memory, particularly human working memory, is the inspiration for the architecture's Navigation Modules and the **TempMap** temporary memory. There is, in fact, variability in human working memory capacity in the population. The study by Friedman et al. (2008) claims that individual differences in executive function and, by implication, human working memory are almost completely genetic in origin. However, despite decades of research on working memory, its measurement still remains uncertain in many regards (Ando et al., 2001; Cowan, 2001; Carruthers, 2013; Ma et al., 2014; Oberauer et al., 2016; Friedman and Miyake, 2017; Chuderski and Jastrzȩbski, 2018). Although only a single **TempMap** memory was required by the CCA5 or CCA6 versions of the architecture for compositional language processing (Schneider, 2024), it is known that in humans, higher working memory capacity is associated with higher intellectual performance (e.g., Aubry et al., 2021). As noted above, Navigation Module B is associated with compositional operations. Thus, the additional temporary memories incorporated into Navigation Module B, as shown in Figure 8, could allow more complex instinctive primitives and more complex learned primitives to emerge that require additional temporary memory storage. This will be explored below.

### 3.2 Duplication of Navigation Module B's

While it is possible to navigate by simple rules/heuristics or similarly generate words of communication by simple rules/heuristics, planning a navigation route, planning words to generate in communication, or planning any other similar task can be advantageous. In any task where planning can improve the outcome, there are usually many possible paths that can be chosen, and it can be very advantageous to run possible plans in parallel.

**Figure 9:**
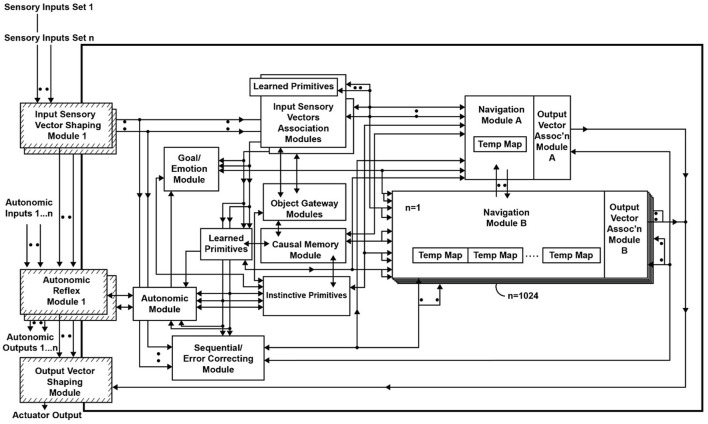
The Causal Cognitive Architecture 7 (CCA7). This is the architecture shown in Figure 8 with duplication (1,024 copies) of Navigation Module B.

Thus, it is hypothesized that another step in the evolution of the Causal Cognitive Architecture could be the duplication of Navigation Module B into many such Navigation Module B's. As noted above, various mechanisms are feasible for the duplication of brain structures (e.g., Rakic, 2009; Chakraborty and Jarvis, 2015). Thus, as the next step in the continued evolution of the CCA6 version of the architecture, there are multiple duplications of the Navigation Module Bs. This is shown in Figure 9. The evolved architecture (i.e., multiple Navigation Module Bs and multiple temporary memories within each of the Navigation Module Bs) is named the Causal Cognitive Architecture 7 (CCA7).

In the CCA7 version of the architecture shown in Figure 9, there are 1,024 copies of Navigation Module B. In every single Navigation Module B, there are 1,024 **TempMap** temporary memories (the number is not shown in the figure). Temporary memories are accessible for the operations of present and future instinctive primitives and learned primitives. Each **TempMap** temporary memory is capable of storing and representing a navigation map.

Consider the well-known traveling salesperson problem where a salesperson, or in this case an agent controlled by a CCA7 version of the architecture, must find the shortest route to visit only once each of a number of different cities and then return to the starting city. This is an NP-hard problem where the number of possible navigation routes to consider in finding the best solution increases exponentially with the number of cities. However, in the CCA7 version of the architecture, given that there are now over 1,000 Navigation Module Bs, then many of the possible routes (or promising routes given the exponential nature of the problem) can be evaluated in parallel, and a more optimal route planned ahead of time. This will be explored below in more detail, including a detailed examination of the CCA7 version of the architecture's internal operations and internal navigation maps.

### 3.3 The traveling salesperson problem

As noted above, in the traveling salesperson example, the salesperson (or, in this case, an agent controlled by a CCA7 version of the architecture) must find the shortest route to visit only once each of a number of different cities and then return to the starting city. The many Navigation Module Bs should allow the CCA7 to evaluate many of the possible (or promising) routes in parallel and plan a more optimal route ahead of time.

For example, if there are a half-dozen cities (or locations or other equivalent destinations) that need to be visited, then this represents (6-1)! or 120 navigation routes (actually, only 60 of these routes need to be considered—returning home to the original city creates a cyclic graph that can be navigated forwards or backwards). If each possible route can be represented in a separate navigation module and there are hundreds of navigation modules in the architecture available, with each running a different combination of routes, then this problem can be solved much faster than if only a single navigation module was available.

If there were, for example, a dozen cities (or locations) that need to be visited, then this represents (12-1)!/2 or nearly 20 million navigation routes to explore to find the best solution. Even with a thousand navigation modules, this would not be enough to run each possible navigation route in a separate navigation module. However, having the thousand navigation modules, in combination with other instinctive primitives and learned primitives of the architecture, can still greatly accelerate a reasonable solution in this case. For example, the nearest neighbor solution algorithm is a relatively simple algorithm where the agent chooses the nearest city (or location) as the next city to visit (Rosenkrantz et al., 1977). However, this algorithm can sometimes give very poor solutions, i.e., very lengthy navigation routes to the problem (Bang-Jensen et al., 2004). However, since there are over a 1,000 different navigation modules, it is possible to consider over a thousand different implementations of the nearest neighbor solution algorithm. Without any sophisticated algorithms (e.g., simply apply random choices for some cities rather than the nearest and, e.g., simply apply various local properties such as avoiding crossings or not avoiding crossings of paths, etc.) by using the over 1,000 navigation modules to run slightly different solutions to the problem, the architecture can better ensure that the solution produced is less likely to be one of the worst solutions.

There is a very large body of literature on solutions to the traveling salesperson problem. A myriad of algorithms have been proposed, including many parallel solutions (e.g., Tschoke et al., 1995). For example, Dorigo and Gambardella (1997) describe using an algorithm based on a colony of ants to find successively shorter routes by laying down pheromone trails. Of interest, for certain variants of the problems, humans can visually produce solutions that are close to the optimal solution (Dry et al., 2006). While the literature gives much more sophisticated possible solutions, the traveling salesperson is considered here simply as an example to illustrate that having multiple navigation modules can be greatly advantageous to various planning strategies the architecture is required to perform.

### 3.4 small_plan() instinctive primitive

As discussed above, instinctive primitives are effectively small procedures operating on the contents of the navigation map(s) in the navigation module(s). The instinctive primitives are inspired by the work of Spelke et al., who have described many innate behaviors in human infants (Spelke, 1994; Spelke and Kinzler, 2007). Human infants do have innate behaviors with regard to simple planning (e.g., Claxton et al., 2003; McCormack and Atance, 2011; Liu et al., 2022). Thus, given the brain-inspired origins of the architecture, it is reasonable that the CCA7 architecture contains an instinctive primitive capable of simple planning (as opposed to learning how to do simple planning via a learned primitive). The CCA7 architecture now includes an additional instinctive primitive “small_plan()” for simple planning.

The instinctive primitive “small_plan()” can use a single Navigation Module B as in the case of the CCA6 version of the architecture (Figure 5), or in the case of the CCA7, it will make use of all the Navigation Module Bs present (which in Figure 9 consists of 1,024 modules). The simultaneous usage of over a thousand navigation modules does not reflect, of course, a similar innate behavior described by Spelke et al. The details and operation of “small_plan()” are discussed in the section below (of course, with education, the CCA7 can acquire learned primitives that allow it better planning strategies, including better algorithms for the solution of the traveling salesperson problem. This is beyond the scope of this paper and is not discussed here).

### 3.5 Operation of the Causal Cognitive Architecture 7 (CCA7)

**Figure 10:**
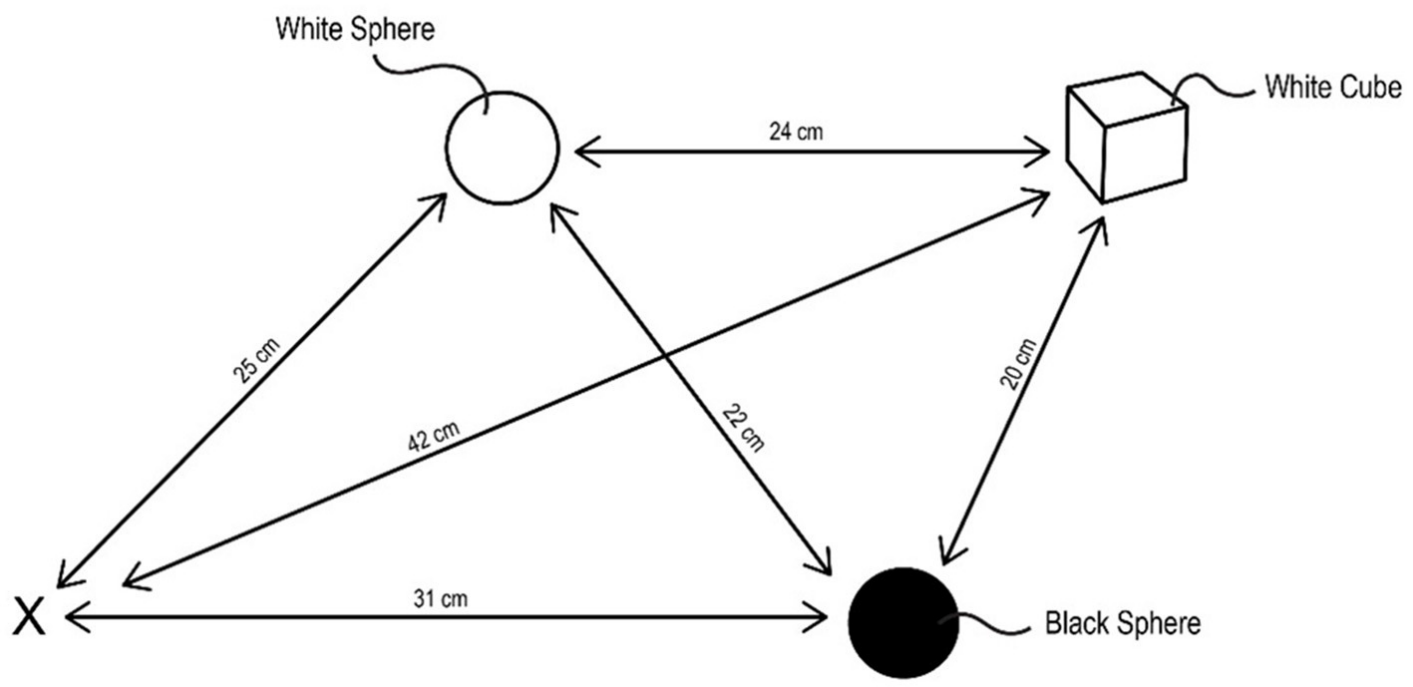
Starting at location “X,” the CCA7 robot must go to the white sphere, the black sphere, and the white cube in any order and then return back to the starting location. The instruction does not specify that the CCA7 should visit each object only once, but this will be implicit in the instinctive primitive triggered, as described in the text. Similarly, the instruction does not specify this, but implicit in the instinctive primitive triggered, it should attempt to do this using the navigation path with the shortest distance.

Consider an agent, i.e., a robot, controlled by the CCA7 architecture shown in Figure 9. For simplicity, the CCA7 architecture and the robot embodiment will be called the “CCA7” or “CCA7 robot.” The CCA7 robot comes to location “X” in Figure 10. It receives the instruction that starting at its existing position (i.e., “X”), it must visit each object and then return to the starting location.

**Figure 11:**
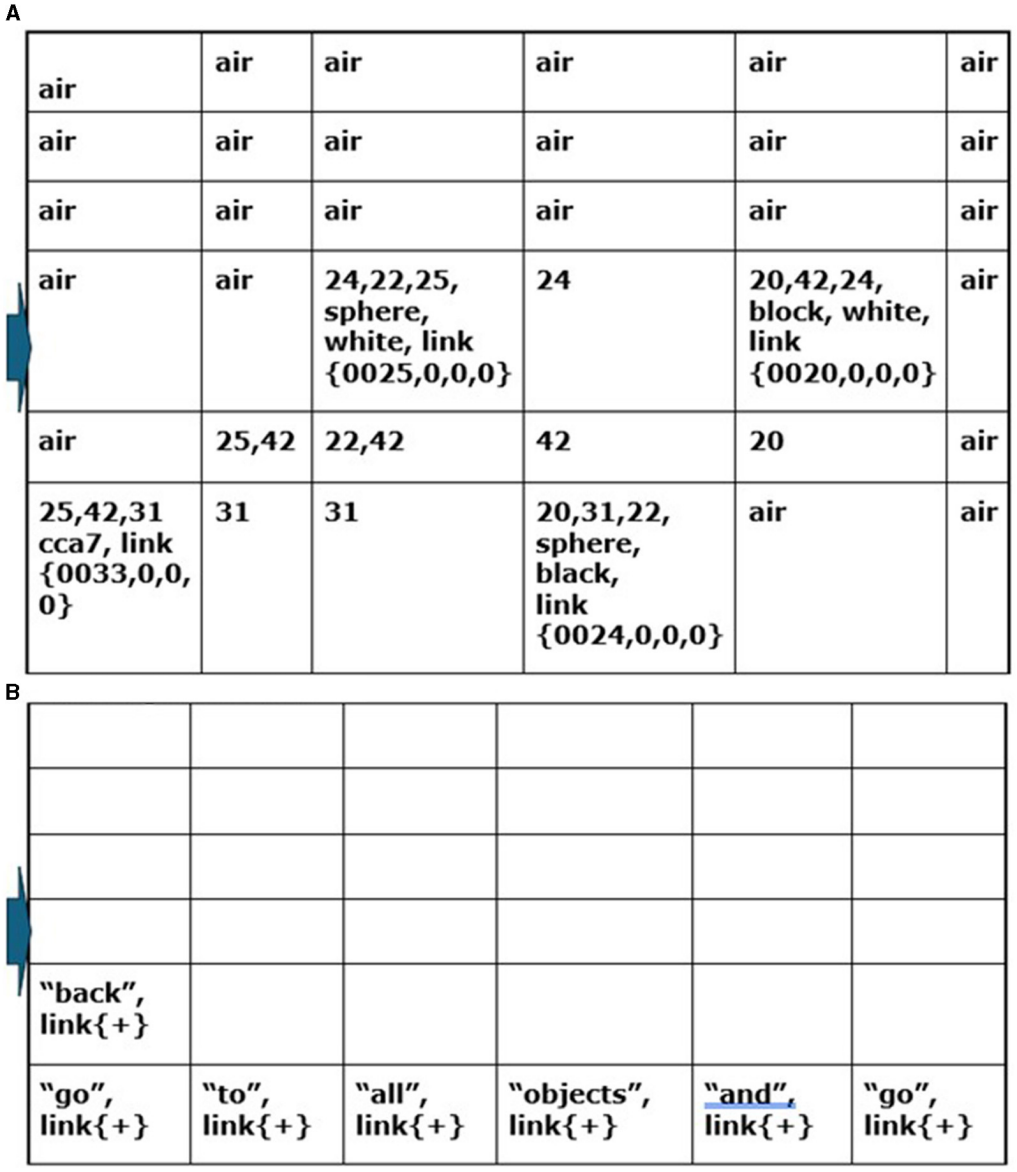
**(A)** While in location “X,” the CCA7 robot maps the sensory scene of Figure 10 into the navigation map in Navigation Module A. The numbers refer to the distance (in centimeters) between the objects in the different cells (the distance number can be determined by matching the same number in the path between two cells. In addition, note a clockwork recording of distances in each cell). **(B)** The instruction “go to all objects and go back” is placed in Navigation Module B *n* = 1, as shown.

While in location “X,” the CCA7 robot maps a sensory scene into the navigation map in Navigation Module A, which is what it automatically does in each available cognitive cycle when there are new sensory inputs to process. The resulting navigation map in Navigation Module A is shown in Figure 11A. The CCA7 robot receives distances (either with the visual sensory information or via a separate ultrasonic distance sensory system). The numbers refer to the distance (in centimeters) between the objects in the different cells (the distance number can be determined by matching the same number in the path between two cells. In addition, note a clockwork recording of distances in each cell). The instruction “go to all objects and go back” is placed in Navigation Module B, as shown in Figure 11B. These operations are similar in nature to ones already described above for the CCA6 version of the architecture in its initial processing of the example of the sensory scene and instruction concerning the “placing a black sphere on top of the black block which is not near a cylinder” (Figures 6, 7).

However, as described above and shown in Figure 9, there are now in the CCA7 multiple Navigation Modules—one Navigation Module A and over a thousand (1,024) duplicated Navigation Module Bs. Equation (115) (taken from Appendix A) indicates that the Working Navigation Map B' **WNMB'** (upon which primitives operate in Navigation Module B) is an array like before, but now can be one of 1,024 different navigation maps (corresponding to a different navigation map in each of the Navigation Module Bs.)

The Navigation Module Bs are numbered *n* = 1 to *n* = 1,023. The top (or first) Navigation Module B appears to be the *n* = 1 Navigation Module B, as shown in Figure 9. However, a *n* = 0 Navigation Module B exists and is used to store a copy of the compositional instructions so that if the other layers are overwritten, there is still a copy of the instructions. Layer *n* = 0 is considered “reserved” and will not be overwritten. If there is other information that an instinctive or learned primitive needs to ensure remains intact for the current operations, other Navigation Module Bs can be temporarily designated “reserved” as well.

Equation (116) indicates that the same instinctive primitive or the same learned primitive (i.e., procedure) is initially applied to all of the Navigation Module Bs. As discussed below, random fluctuations can be introduced in the different Navigation Module Bs to produce a variety of results to choose from. In the example below (i.e., a traveling salesperson problem), the same instinctive primitives are used by all the Navigation Module Bs, and this does not cause any particular issues. However, in other types of problems, in subsequent cognitive cycles, the initial primitive applied may trigger different primitives in different Navigation Modules. This issue is discussed below in the Section 6.

At present, there is no energy-saving operation or Autonomic Module (Figure 9) interaction to turn off the multiple Navigation Module Bs and use only a sole Navigation Module B *n* = 0 or *n* = 1, i.e., much like the previous CCA6 version of the architecture functioned. However, if an instinctive primitive or learned primitive does not require the thousand-plus Navigation Module Bs, it can simply ignore the results in the multiple modules and use the results of operations in Navigation Module B *n* = 1. This is also discussed below in the Section 6.

As shown in Figure 11A, Navigation Module A contains the Working Navigation Map (**WNMA**) of the sensory scene of the various places the agent has to navigate to. In Navigation Module B, *n* = 1 (Figure 11B) is a Working Navigation Map (**WNMB**_n = 1_) of the instruction sentence to “go to all the objects and go back.”

The word “go” in the first cell of the navigation map in Navigation Module B (Figure 11A) is matched against the Causal Memory Module as an action word and triggers the instinctive primitive “goto()” (Equation 117). “**WNMA'**_*t*_ = Nav_ModA.goto()” indicates that this instinctive primitive, i.e., “goto(),” is being applied to the Working Navigation Map A in Navigation Module A.

**Figure 12:**
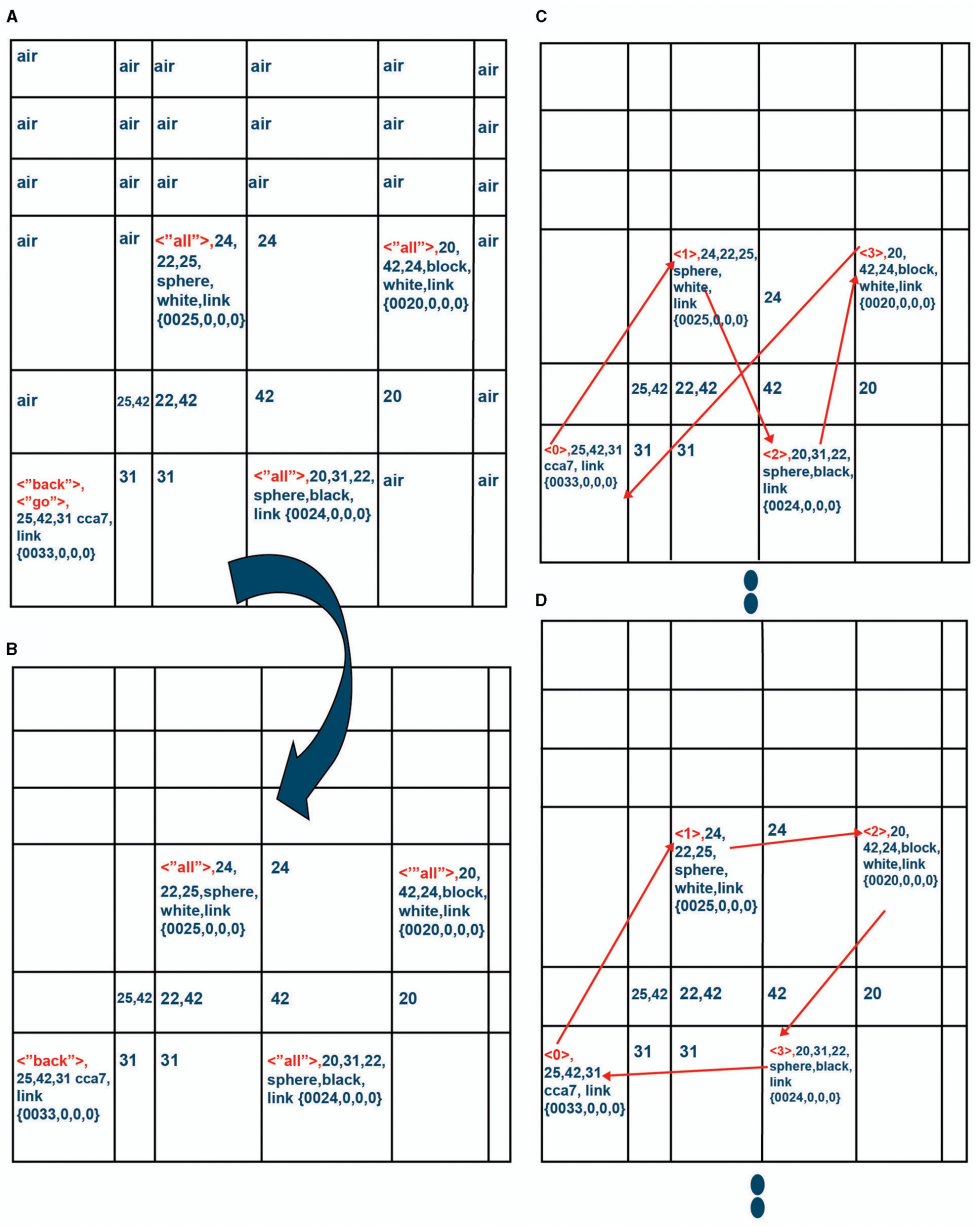
**(A)** The navigation map in Navigation Module A is tagged via the operation of the “goto()” instinctive primitive operating on the navigation maps in Navigation Module A and Navigation Module B. **(B)** The instinctive primitive “small_plan()” copies Navigation Module A into the 1,023 (*n* = 1..1023) navigation maps in Navigation Module Bs, removing any action words and just leaving the tagged cells to navigate to. One of the navigation maps of Navigation Module B is shown here. **(C) Navigation Module B** ***n*** **=** **1:** The instinctive primitive “small_plan()” is used as the starting point for the tagged cell and considers which distance to another tagged cell (i.e., representing an object) is the shortest. The cell that has the shortest distance is tagged with a < 1>. Then, it considers cell < 1> as the starting point and considers which distance to another tagged cell is the shortest. This continues until all the tagged cells in the navigation map are considered and re-tagged with a number indicating in which order they should be navigated. After all cells are navigated, there is a return to starting position which has been tagged with a < 0>. This simple nearest neighbor algorithm occurs in the navigation map in the *n* = 1 Navigation Module B. Note that the sum of the distances is 25+22+20+42 = 109 cm in this navigation map. **(D) Navigation Module B** ***n*** **=** **2:** The simple nearest neighbor algorithm occurs again in the navigation map in the *n* = 2 Navigation Module B; however, random fluctuations have been introduced (see text). The instinctive primitive “small_plan()” considers the starting tagged cell and considers which distance to another tagged cell (i.e., representing an object) is the shortest. Random fluctuations are now introduced. Note that when deciding which cell to navigate to after cell (2,2,0) containing the white sphere, the random fluctuation introduced here causes “small_plan()” to choose cell (4,2,0) even though the distance of 24 to that cell was not the nearest neighbor. Note that the sum of the distances is 25+24+20+31 = 100 cm in this navigation map.

The instinctive primitive “goto()” causes the CCA7 robot to tag a location(s) and then essentially move to whatever location is indicated by the tag(s). The word “all,” which is associated with the active word < “go”> (until another action word is encountered, as in an earlier example above), will cause the tag < “all”> to be placed in all the cells with objects in the navigation map in Navigation Module A (Figure 12A).

The words “go back” are also associated with the instruction word < “go”> and will cause the tag < “back”> to be placed in the starting cell [which is (0,0,0) in this example]. This can be seen in Figure 12A.

Once the instinctive primitive “goto()” tags the cell(s) where it has to move to, it then decides if it will move (i.e., “go to”) the cell with the tag. However, if there are multiple tags, i.e., multiple locations to navigate to [“locations > 1” in Equations (118–123)], then the “small_plan()” instinctive primitive is activated instead of moving to a single location. As discussed above, this instinctive primitive will plan a navigation route to whatever multiple tagged locations are indicated on the navigation map(s).

Once activated (Equation 118), the instinctive primitive “small_plan()” (regardless of its argument) will copy the Navigation Map A to all “non-reserved” Navigation Module Bs, i.e., *n* = 1… 1,023 in this example. It will remove any action words such as “go” in the example above. This copying is indicated by the arrow in Figure 12. Thus, the instruction sentence in Navigation Module B *n* = 1 (Figure 11B) is overwritten here (Navigation Module B *n* = 0 is “reserved” for a copy of the instruction sentence, although it actually will not be used again in this example). A number of existing operations in various instinctive primitives have properties whereby they compare the contents of Navigation Module A and Navigation Module B with each other. Thus, the emergence of this step is a feasible one in the continued evolution of architecture.

Equation (118) describes instinctive primitive “small_plan(random=False)” acting on the Working Navigation Map (**WNMB'**_t, *n* = 1_) in Navigation Module B *n* = 1 (Nav_ModB_n = 1_) at time t = t (i.e., during the current cognitive cycle). The instinctive primitive “small_plan(random=False)” follows the nearest neighbor algorithm discussed above. In making a plan where to navigate, this primitive will choose the tag (i.e., location) that is closest to the tag (i.e., location) from where it is navigating. The argument random = False indicates that this instinctive primitive does not introduce any random variations. As will be seen below, in the other Navigation Module Bs, *n* = 2…1,023 random variations will be introduced.

In the *n* = 1 Navigation Module B, “small_plan(random=False)” operates on the navigation map shown in Figure 12B and determines which tagged cell to navigate first. It uses a nearest-neighbor algorithm in its planning actions. For example, in Figure 12B, the cell (0,0,0) in which the CCA7 is starting has a distance of 25, 31, and 42 units (actually centimeters, but “small_plan()” will disregard the actual units) to the other objects [they are listed as “25, 42, 31” in cell (0,0,0) in Figure 12B due to a clockwise organization of distances]. According to the nearest neighbor algorithm, it chooses the shortest distance, which is 25, i.e., it plans to navigate first to cell (2,2,0) containing the white sphere. Thus, it changes the < “all”> tag to a < 1>. This can be seen in Figure 12C.

The instinctive primitive then considers navigating from cell (2,2,0)—which object to navigate to next? As can be seen in Figures 12B, C, 22 is the shortest distance; thus, it decides to navigate to cell (3,0,0), which contains the black sphere. It changes the < “all”> tag to a < 2>. It then considers navigating from cell (3,0,0)—which object should be navigated to next? Actually, the only untagged object remaining is the white block in cell (4,2,0), which is then tagged with a < 3>. This is shown in Figure 12C.

If this was the previous CCA6 version of the architecture (albeit retrofitted with these new equations) with only one Navigation Module B, then at this point, the instinctive primitive “small_plan(random=False)” would trigger the instinctive primitive “move()” to move a CCA6 robot to cell (2,2,0) containing the white sphere. Then, the instinctive primitive “move()” is triggered again to move to cell (3,0,0) containing the black sphere. Next, the instinctive primitive “move()” is triggered again to move to cell (4,2,0) containing the white block. Finally, the instinctive primitive “move()” is triggered again to move to cell (0,0,0), which was the starting point. From Figure 12C, note that the sum of the distances is 25+22+20+42 = 109 cm in this navigation route.

However, in the CCA7 version of the architecture being considered here, there are over a thousand Navigation Module Bs. As Equation (120) indicates, for Navigation Module B *n* = 2… 1,023, the instinctive primitive “small_plan(random=weight_distance)” will perform a similar nearest neighbor planning algorithm in the other modules for this same navigation map (Figure 12B). However, as indicated by the argument random = weight_distance random fluctuations are introduced now, so a slightly different navigation route may occur in different Navigation Module Bs *n* = 2… 1,023 (Figure 12D).

The instinctive primitive “small_plan(random=weight_distance)” follows a similar nearest neighbor algorithm to the one described above. However, now random fluctuations may (or may not) be introduced at each step a navigation decision is made. These fluctuations are weighted by distance position, as explained below. Normally, the destination with the shortest distance will be chosen, as seen above for Navigation Module B *n* = 1 (Figure 12C). Here, this is likely to occur also, but some randomness means another destination can be chosen (e.g., Figure 12D), although the destinations the farthest away are the least likely to be chosen as the next destination, as will be shown below.

Consider that at any given decision point, the list ***destination*** contains sorted destinations [a, b, c, d, e…], which still can be navigated to Equation (124). This list is sorted by distance such that navigation to destination “a” is the shortest, navigation to destination “b” is the next shortest, and so on (Equation 125). The value a in the list is the distance to destination “a,” the value b in the list is the distance to destination “b,” and so on.

Consider an example where there are five possible destinations which the CCA7 can now navigate to from some starting point, i.e., to object “a,” to object “b,” to object “c,” to object “d,” or to object “e.” As per Equation (124), ***destination*** = [a, b, c, d, e], where the distance from the starting point to “a” is less than or equal to the distance from the starting point to “b,” and so on (Equation 125). Object “a” represented by element a in ***destination*** is considered to have position = 1 in the list, while object “b” has position = 2, and so on. Similarly, object “a” is considered to have inverse_position = 5 in this list, while object “b” has inverse_position = 4, and so on (Equation 124).

Equation (126) shows that when the instinctive primitive “small_plan(random= weight_distance)” is triggered, a parameter “*weight*” is given a value of 4. Equation (127) shows that when the instinctive primitive “small_plan(random= weight_distance)” is triggered, the probability of selecting destination “x” to navigate to is given by “probability_destination_x_” which can be computed as “inverse_position${\;}_{\text{x}}^{\wedge }$^*weight*^/∑inverse_position^∧^^*weight*^.”

Continuing with the example above of choosing to navigate to locations “a,” “b,” “c,” “d,” or “e,” consider Equations (126, 127). Consider navigating to the first destination “a” (which is the shortest navigation path from the starting point since it is the first element in ***destination***). Thus, as per (Equation 127), x = “a” and the value of the term “inverse_position${\;}_{\text{a}}^{\wedge }$^*weight*^” is thus 5^∧^^*weight*^. The parameter *weight* is 4 (Equation 126); thus, the value of the term “inverse_position${\;}_{\text{a}}^{\wedge }$^*weight*^” is 5^∧^4, or 625. Similarly, the value of all the inverse positions raised to the fourth power (*weight* = 4) added up, i.e., “∑inverse_position^∧*weight*^” is 625+256+81+16+1 = 979 (Equation 127).

In the actual CCA7 version of the architecture, other than as needed internally (and encapsulated) for artificial neural networks being used, only very simple arithmetic is explicitly available. Thus, in Equation (127), the “probability_destination_x_” is shown as being approximately equal to a term that must be calculated via high exponential powers and involves the manipulation of many decimal places. While Equation (127) is fine for some simulations of the architecture, the relationship shown in Equation (127) can be achieved more realistically by the architecture by making use of stored probability distributions (see below). A limited number of such probability distributions can approximate (Equation 127) when deciding which object or city to navigate next to in a planning task.

Continuing with the example above of choosing to navigate to locations “a,” “b,” “c,” “d,” or “e,” the instinctive primitive “small_plan(random= weight_distance)” has just been triggered. Thus, *weight* is given a value of 4 (Equation 126). The probability of the algorithm in this instinctive primitive choosing, for example, destination “a” to navigate next to, is probability_destination_a_. By Equation (127), this is equal to “= inverse_position${\;}_{\text{a}}^{\wedge }$^*weight*^/∑ inverse_position^∧^^*weight*^.” Above, the term “inverse_position${\;}_{\text{a}}^{\wedge }$^*weight*^” was calculated to be 625, and the term “∑inverse_position^∧*weight*^” to be 979. Thus, the probability of the algorithm in this instinctive primitive to choose destination “a” to navigate next to is 625/979, or 64%.

From similar calculations, the probability of choosing any of these sorted destinations (i.e., “a” is closer and “e” is the farthest away from the starting point) in this example of [a, b, c, d, e] is [64, 26, 8, 2,…0.1%]. Thus, when small_plan(random=weight_distance) is used in this example, of the five potential destinations to choose from in [a, b, c, d, e], there is a 64% chance of navigating to the nearest neighbor “a” and a 26% chance of navigating to the next nearest neighbor “b,” but only a 0.1% chance of navigating to the farthest neighbor “e.”

In Equation (128), it can be seen that when the instinctive primitive “small_plan(random= False)” is triggered, a parameter “*weight*” is given a value of 30. The result of this high *weight* is that the nearest neighbor destination is always used, i.e., there is no randomness (Equation 129). Thus, the probability distribution for navigation to potential objects/cities [a, b, c, d, e] is [100%, 0, 0, 0, 0], i.e., there is a 100% chance of choosing an object/location “a” to navigate, and 0% chance of choosing object/location “b,” “c,” “d,” or “e” to navigate.

As noted above, if there is only one Navigation Module B in the system, or if this is a CCA7 version of the architecture and this is Navigation Module B *n* = 1, then as Equation (118) indicates, the instinctive primitive “small_plan(random=False)” is triggered. The nearest neighbor (i.e., shortest distance) from the starting point of the cell (0,0,0) (Figure 12C; there is a < 0> put in that cell) is cell (2,2,0)—there is a < 1> put tag in that cell. The next nearest neighbor is cell (3,0,0)—there is a < 2> put tag in that cell. The next nearest neighbor is the only one left, which is cell (4,2,0)—there is a < 3> tag put in that cell. Then, with no more active cells to navigate to the left, there is navigation back to the starting point of (0,0,0)—there is a < 0> tag there.

Once all cells are tagged, the instinctive primitive “small_plan(random=False)” would trigger the instinctive primitive “move()” to move a CCA7 robot to the tagged cells (Equations 122, 123). The instinctive primitive “move()” first moves the CCA7 to cell (2,2,0) with the tag < 1> containing the white sphere. Then, the instinctive primitive “move()” is triggered again to move to cell (3,0,0) with the tag < 2> containing the black sphere. Next, the instinctive primitive “move()” is triggered again to move to cell (4,2,0) with the tag < 3> containing the white block. Finally, the instinctive primitive “move()” is triggered again to move to cell (0,0,0) containing the tag < 0>, which was the starting point. From Figures 10, 12C, note that the sum of the distances is 25+22+20+42 = 109 cm in this navigation route.

Now consider the Navigation Module Bs *n* = 2…1,023 in the CCA7 version of the architecture. In Figure 12D, Navigation Module B *n* = 2 is shown. As Equation (120) indicates, the instinctive primitive “small_plan(random=weight_distance)” is triggered. As before, the instinctive primitive “small_plan()” considers which possible destination it can navigate to will be the shortest, albeit now with a random fluctuation introduced. From the starting point of the cell (0,0,0), the CCA7 can navigate next to cells (2,2,0), (3,0,0), or (4,2,0). From Figure 10, it can be seen these correspond to possible distances of 25, 31, and 42 cm.

As discussed above, “small_plan(random=weight_ distance)” will introduce a random fluctuation in deciding which object/location to navigate to via Equations (124–127). The sorted list is [25, 31, 42] (Equations 124, 125). The sum of the inverse positions is 3^∧^4+2^∧^4+1^∧^4 or 98, and thus the probability distribution is [81/98,16/98,1/98] or [83%, 16%, 1%]. The likelihood of navigating to the first position destination of 25 cm corresponding to cell (2,2,0) is 83%, while the probability of navigating to (3,0,0) is 16% and the probability of navigating to (4,2,0) is 1%. A cumulative probability distribution results essentially from considering these probabilities: [ = < 83%, = < 99%, = < 100%]. A random number between 0 and 1 is obtained, which happens to be, for example, 0.55 or 55%. It is within the 83% cumulative probability of the first position destination. Thus, the CCA7 architecture tags cell (2,2,0) with a < 1>.

The instinctive primitive “small_plan(random= weight_distance)” must consider navigating to the next object/location. From the starting point of cell (2,2,0), the CCA7 can navigate next to cells (3,0,0) or (4,2,0). From Figure 10, it can be seen that these correspond to possible distances of 22 and 24 cm. The sum of the inverse positions is 2^∧^4+1^∧^4 or 17, and thus the probability distribution is [16/17,1/17] or [94%, 6%]. The likelihood of navigating to the first position destination of 22 cm corresponding to cell (3,0,0) is 94%, while the probability of navigating to (4,2,0) is 6%. A cumulative probability distribution results essentially from considering these probabilities: [ = < 94%, = < 100%]. A random number between 0 and 1 is obtained which happens to be, in this case, 0.95 or 95%. Thus, instead of navigating to the nearest neighbor (3,0,0), the CCA7 will tag the second nearest neighbor, i.e., (4,2,0), as the next destination to navigate to with a < 2> (Figure 12D). The next nearest neighbor is the only one left, which is cell (3,0,0)—there is a < 3> tag put in that cell. Then, with no more active cells to navigate to the left, there is navigation back to the starting point of (0,0,0)—there is a < 0> tag there. The tagged cells can be seen in Figure 12D.

From Figures 10, 12D note that the sum of the distances is 25+24+20+31 = 100 cm in this navigation route. Thus, even though this route ended up taking a path between two locations which was not the shortest distance [i.e., going from cell (2,2,0) to cell (4,2,0), which was 24 cm rather than going to cell (3,0,0), which was 22 cm], it turned out that the total distance in navigating to all object/locations turned out to be shorter than the path obtained in Navigation Module B *n* = 1 where the nearest neighbor algorithm was followed at each decision point.

Similar algorithms are also running in the other Navigation Module Bs at the same time. The total distance sum obtained in each Navigation Module B is transferred to the **TempMap** memory areas of Navigation Module B *n* = 0 (Equations 119, 121). Although there are many more **TempMap** memory areas now available in the CCA7 version of the architecture, this instinctive primitive actually just keeps track of the navigation map number (i.e., which “*n*” from *n* = 1… 1,023), which has yielded the smallest total distance of the best (i.e., shortest) navigation plan found (Equation 122). Thus, **Nav_ModB**_n = best_, where “best” is the Navigation Module B “*n*,” showed the shortest total navigation distance.

The instinctive primitive “small_plan()” then activates the instinctive primitive move (**WNMB'**_t, *n* = *best*_) (Equation 123). To continue the above example, “best” is Navigation Module B *n* = 2, i.e., shown in Figure 12D (the total distance here was 100 vs. 109 cm in Navigation Module B *n* = 1, and vs. 119 cm obtained in other Navigation Module Bs). It will then repeatedly trigger the instinctive primitive “move()” to navigate to < 1> (the white sphere), then < 2> (the white cube), then < 3> (the black sphere), and then return to the starting cell < 0>.

This navigation planning example involves navigating to three locations and then returning back to the starting position. Thus, there are only a handful of possible variations in navigation to consider, and thus, despite the random fluctuations, many variations will repeat among the over thousand Navigation Module Bs. However, many real-world problems may involve more locations (or social situations or other analogous “locations”) than this simple problem. In the next section, the CCA7 architecture will be applied to a larger dataset of navigation locations.

The instinctive primitive “small_plan()” effectively helps to decide what sequence to perform operations in. While such problems can be physically moving to different locations, they can also range from navigating in the social hierarchy space of society to navigating through an idea space of more abstract concepts. With regard to the traveling salesperson problem or other particular planning problems, note that with education (i.e., assumes a larger set of instinctive primitives than exist at present and acquisition of more basic concepts), the CCA7 can acquire learned primitives that are more specific and more sophisticated for particular planning purposes.


(115) $$\begin{array}{l} {\text{WNMB'}}_{\text{n}=0\ldots 1023}=\epsilon R^{\text{m}xnxoxp} \end{array}$$

(116) $$\begin{array}{l} \left({\text{WNMB'}}_{\text{n}=\text{x}}\neq {\text{reserved AND WNMB'}}_{\text{n}=\text{y}}\neq \text{reserved}\right)\\ \Rightarrow {initial\text{\_}primitive}_{\text{WNMB'}n=x}={initial\text{\_}primitive}_{\text{WNMB'}n=y} \end{array}$$

(117) $$\begin{array}{l} \;<“go\text{”}>\\ \{\Rightarrow {\text{WNMA'}}_{t}=\text{Nav\_ModA.goto()} \end{array}$$

(118) $$\begin{array}{c} \text{locations}>\;1,\\ {}[\Rightarrow \text{WNM}{{\text{B}}^{\prime }}_{\text{t},\text{n=1}}\\ =\text{Nav}\_\text{Mod}{\text{B}}_{\text{n=1}}.\text{small}\_\text{plan}(\mathrm{random}=\text{False}) \end{array}$$

(119) $$\begin{array}{l} \left(\Rightarrow {\text{TempMap}}_{{\text{WNMB'}}_{\text{n}=0}}=\text{minimum}\left({\text{total\_distance}}_{\text{n}=1}\right)\right) \end{array}$$

(120) $$\begin{array}{c} \Rightarrow {\text{WNMB'}}_{\text{t},n=2\ldots 1023}\\ ={\text{Nav\_ModB}}_{\text{t},n=2\ldots 1023}.\\ \text{small\_plan(random=weight\_distance)} \end{array}$$

(121) $$\begin{array}{l} \left(\Rightarrow {\text{TempMap}}_{{\text{WNMB'}}_{\text{n}=0}}=\text{minimum}\left(\text{total\_}{\text{distance}}_{\text{n}=2\ldots 1023}\right)\right) \end{array}$$

(122) $$\begin{array}{l} \Rightarrow best={\text{TempMap}}_{{\text{WNMB'}}_{\text{n}=0,\text{minimum}\left(\text{total\_distance}\right)}} \end{array}$$

(123) $$\Rightarrow \text{Nav}\_{\text{ModB}}_{\text{n=best}}.\text{move}(WNM{B^{\prime }}_{\text{t},\text{n=best}})]\}$$

(124) $$\begin{array}{l} \text{destination}=\left[a,b,c,d,e\ldots \right] \end{array}$$

(125) $$\begin{array}{l} {\text{distance}}_{\text{a}}<={\text{distance}}_{\text{b}},{\text{distance}}_{\text{b}}<={\text{distance}}_{\text{c}},\ldots .\; \end{array}$$

(126) $$\begin{array}{r} \text{small}\_\text{plan}(\text{random}=\text{weight}\_\text{distance}),\\ \{\Rightarrow weight=4 \end{array}$$

(127) $$\begin{array}{c} \Rightarrow \text{probability}\_{\text{destination}}_{\text{x}}\approx \text{inverse}\_{\text{position}}_{\text{x}}^{\wedge weight}\\ /\sum \text{inverse}\_{\text{position}}^{\wedge \text{weight}}\} \end{array}$$

(128) $$\begin{array}{r} \text{small}\_\text{plan}(\text{random}=\text{False}),\\ \{\Rightarrow weight=30 \end{array}$$

(129) $$\Rightarrow \text{probability}\_{\text{destination}}_{\text{a},\text{weight}>\text{9}}=1\}$$

## 4 Methods

### 4.1 Computer simulation of the Causal Cognitive Architecture 7 (CCA7)

The Equations (1–129; Appendix A) are computer-simulated via the Python language to represent the CCA7. The computer simulation does not interface with real-time actual video camera or microphone inputs or with real robotic actuators. Sensory inputs are simulated in all simulations, and actuator outputs are similarly simulated.

The navigation maps in the Python simulation have 6x6x0 dimensions (although internally, a larger number of dimensions are actually used to represent the segmentation of objects and binding with motion and action). As noted above, navigation maps are essentially arrays. Thus, the more efficient Numpy library (Harris et al., 2020) is called by the Python program for most operations on the navigation maps. For future larger simulations of the architecture, more classical deep learning software and hardware can be used. However, in the current simulation, the FuzzyWuzzy string matching library (via pypi.org) is used for pattern matching.

The Python simulation of the architecture at this time contains a very limited set of instinctive primitives. It mainly contains the ones specified in Equations (1–129; Appendix A), which relate to very basic operations and the ability for causal reasoning, analogical induction, compositionality, and, as discussed above in the section on new work, simple planning. At this time, instinctive primitives must be hand-crafted. Automated methods for instinctive primitive creation are being explored.

Here, Python version 3.11 is used. The parallel elements of Equations (1–129; Appendix A) are simulated sequentially—a new cognitive cycle starts when all the operations of the previous cognitive cycle have been completed.

The main purpose of this computer simulation is to show that the operation of the CCA7 version of the architecture is feasible, particularly its ability to perform planning operations. The simulation, i.e., based on the representation of the CCA7 version of the architecture via Equations (1–129; Appendix A), can be tested below on a traveling salesperson dataset. The distances between a starting city and a dozen other cities are given in Supplementary Table B1 (Google-OR-Tools, 2023). The results are discussed below.

### 4.2 Alternative weightings for the probability distribution of the next destination

The literature on the traveling salesperson problem is vast, and there are many strategies for choosing the next location to navigate (e.g., as mentioned above—Tschoke et al., 1995; Dorigo and Gambardella, 1997; Dry et al., 2006). Both strategic decisions and random fluctuations can be introduced into the solution algorithm in many ways. As noted above, the traveling salesperson problem is considered here simply as an example to illustrate that having multiple navigation modules can be greatly advantageous to various planning strategies the architecture is required to perform. Nonetheless, it is useful to consider how random fluctuations are inserted into the planning decisions. Of interest is that the positional weighting used in Equations (124–127) does not take into account the relative values of the different distances, e.g., [12, 13, 44] will be weighted the same as [12, 42, 44], i.e., the probability of the choosing the location that is 42 units away will be the same as choosing the location that is 13 units away.

The reason for using the positional weighting is that the actual CCA7 version of the architecture only explicitly has access to very simple arithmetic. Thus, in weighting the probability distributions, pre-stored distributions were used, which could readily be accessed rather than involve complex calculations. Although Equations (124–127) are used in the Python simulation of the architecture (albeit necessitating the Python “Decimal” class due to the many digits created by the high-power exponents), the architecture can simply access a limited number of probability distributions based on the positions of the nearest city/location/object in a list, with no complex arithmetical calculations required.

It is possible, of course, to weight by the relative values: calculate the reciprocals of the difference of each number (nominal value of 1) in the list from the smallest value (thus, a smaller difference will give a larger reciprocal) and normalize as probabilities. For example, if there are three possible locations to navigate to with distances (arbitrary units not specified) of [12, 13, 44], then the weight probabilities would be normalize ([1/1, 1/1, 1/32]) or [49, 49, 2%]. Thus, there would be a 49% chance of navigating to the location 12 units away vs. a 2% chance of navigating to the location 44 units away. The probability distribution for the example above of three possible locations [12, 42, 44] is normalize ([1/1, 1/30, 1/32]) or [94, 3.1, 2.9%]. In contrast, the positional probability distribution [i.e., via Equations (124–127)] of either example yields [83, 16, 1%], i.e., 83% chance of navigating to the first-closest location, 16% chance of navigating to the second-closest location, and a 1% chance of navigating to the third-closest location, regardless of the actual distances.

In a modified computer simulation, random fluctuations are introduced by comparing a normalized random number with the probability distributions calculated via relative weights as discussed above:

a. Calculate the reciprocals of the difference of each number (nominal value of 1) in the list from the smallest value;
b. Normalize the probabilities.

This alternative “value weighted” version can also be tested below on a traveling salesperson dataset. The distances between a starting city and a dozen other cities are given in Supplementary Table B1 (Google-OR-Tools, 2023). The results are discussed below.

### 4.3 Comparative experiments

As noted above, the CCA7 architecture, functionally based on a possible further evolution of the brain as modeled by previous versions of the Causal Cognitive Architecture, is expected to be able to perform simple planning in terms of navigating to multiple locations with a certain degree of higher efficiency than if the previous versions of the architecture were used.

Computer simulations of the CCA7 architecture can be tested on a traveling salesperson dataset. The distances between a starting city and a dozen other cities are given in Supplementary Table B1 (Google-OR-Tools, 2023).

The optimal (i.e., shortest) solution obtained via brute force (non-CCA7) computation is 7,293 miles (the data in Supplementary Table B1 of distances between the cities was given in miles). The route giving this shortest path is City #0,7,2,3,4,12,6,8,1,11,10,5,9,0.

The traveling salesperson problem city data from Supplementary Table B1 was simulated as sensory input data to the CCA7 architectures. The same CCA7 version of the architecture shown in Figure 9 was used. However, Equations (126–129) were modified in different runs of the architecture as described below. As described above, the CCA7 architecture, via the instinctive primitives associated with Equations (126–129), attempts to produce the shortest path in a planning problem. In the case of the city data from Supplementary Table B1, the architecture attempts to produce the shortest path to navigate once to the dozen cities listed in Supplementary Table B1 and return back to the starting city (i.e., 13 cities in total).

The following questions were asked, and the accompanying comparative experiments were then performed:

a. The position-weighted algorithm used to inject random fluctuations into the nearest neighbor algorithm (Equations 126–129) uses a *weight* parameter to create a probability distribution to select the next destination to navigate to. At present, a *weight* value of 4 is used.

What is the effect of varying the *weight* parameter on the shortest path yielded in the traveling salesperson problem, i.e., is the value of *weight* used in Equation (126) a reasonable one based on a typical planning problem represented by the data in Supplementary Table B1?

b. Do the multiple Navigation Module Bs allow better planning in terms of this traveling salesperson problem represented by the data in Supplementary Table B1?

Multiple runs to ensure statistical significance (or insignificance) of the following are to be examined: the shortest distance obtained by a CCA7 architecture modified to use only 1 Navigation Module B vs. a CCA7 architecture using 1,023 Navigation Module Bs.

Note: When multiple Navigation Module Bs are used, Navigation Module B *n* = 0 is restricted to holding a copy of any instructions; hence, 1K-1 results in 1,023 Navigation Module Bs available.

Note: As per Equation (118), Navigation Module B *n* = 1 uses the instinctive primitive small_plan(random=False); thus, *weight* is set to 30 for this case, i.e., nearest-neighbor algorithm without any random fluctuations. However, the Navigation Module Bs *n* = 2… 1,023 per Equation (120) use the instinctive primitive small_plan(random=weight_distance); thus, *weight* is set to 4 for these Navigation Modules, and there will thus be the possibility of random fluctuations injected in choosing the next destination city at every decision point.

Note: Due to the generation of high exponents and large decimal arithmetic seen in Equation (127), when the *weight* parameter exceeds 9, as per Equation (129), the probability of choosing the shortest distance becomes 100%, i.e., nearest-neighbor algorithm without any random fluctuations is used.

c. Do higher quantities of Navigation Module Bs result in significantly better results?

Multiple runs to ensure statistical significance (or insignificance) of the following is to be examined: the shortest distance obtained by a CCA7 architecture using 1,023 (i.e., “1K”) Navigation Module Bs vs. versions of the architecture using 4,095 (i.e., “4K”) and 16,383 (i.e., “16K”) Navigation Module Bs.

Note: Navigation Module B *n* = 0 is restricted for holding a copy of any instructions, and thus the availability of 1K-1, 4K-1, and 16K-1 Navigation Module Bs, which are rounded and simply referred to as “1K,” “4K,” or “16K,” respectively.

d. Does a “value-weighted” algorithm to introduce random fluctuations (i.e., Section 4.2) give better results (i.e., a shorter distance) than the “position weighted” algorithm [i.e., Equations (124–127)]?

Multiple runs to ensure statistical significance (or insignificance) of the following is to be examined: the shortest distance obtained by the usual “position weighted” version of the CCA7 architecture using 1,023 Navigation Module Bs vs. the shortest distance obtained by “value weighted” version of the architecture.

Note: “Position weighted” refers to Equations (124–127), which create a probability distribution of which location to choose next in deciding where to navigate to, simply in terms of which location is the nearest, the next-nearest, the third-next-nearest, and so on, without considering the actual values (i.e., there is a relative ranking but without consideration of any scale) of the distances. In contrast, “valued weighted” refers to the modifications of these equations given by Section 4.2 such that the reciprocals of the difference of each distance value from the smallest value (i.e., its actual value and the actual value of the smallest distance, rather than just its position of where it is relative to the other possible destinations) are calculated and normalized to create a probability distribution of which location to choose next in deciding where to navigate to.

## 5 Results

### 5.1 Effect of varying the *weight* parameter

As noted above, the position-weighted algorithm creates random fluctuations in the nearest neighbor algorithm (Equations 126–129). Lower *weight* values (Equation 126) make it less likely that the closest next destination will be navigated to, i.e., more likely another destination will be selected. At present, a *weight* value of 4 is used. To see if this value is reasonable in terms of a typical planning problem, as represented by the data in Supplementary Table B1, the shortest distance obtained by the CCA7 for different values of the *weight* parameter was examined.

Supplementary Table B2 shows the shortest distance obtained by single runs of the CCA7 architecture for *weight* values varying from 1 to 30. The results shown in the column on the right side are for a version of the architecture with the full set of Navigation Module Bs *n* = 1… 1,023. For comparison, in the column on the left, only a single Navigation Module B is used, so rather than restrict it to Equations (118, 119) which will yield each time a value of 8,131 miles per the nearest-neighbor algorithm, it is also running Equations (120, 121), i.e., small_plan (random=weight_distance).

**Figure 13:**
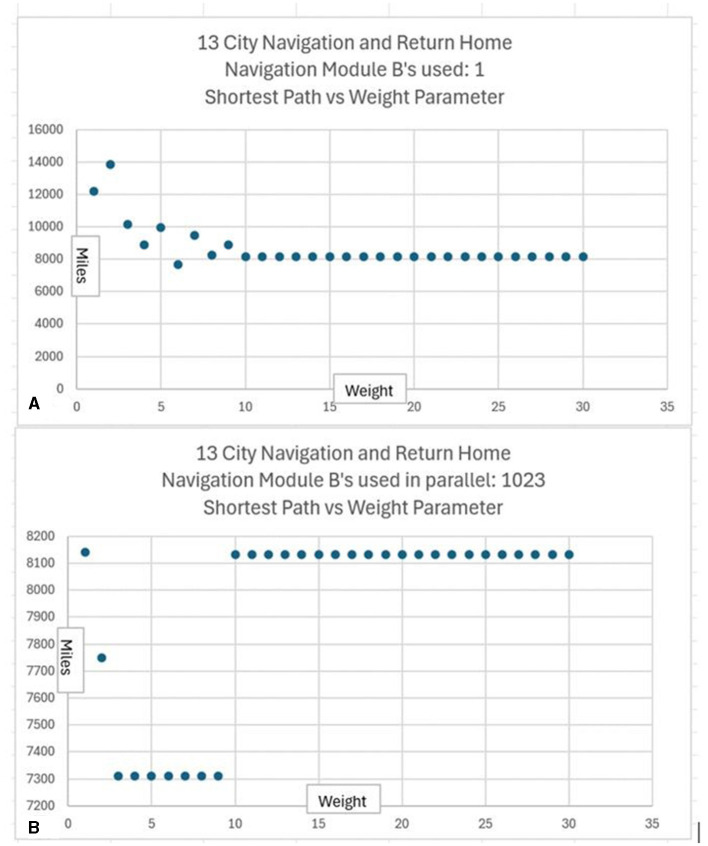
Simulation of the CCA7 robot navigating from a home city to 12 other cities (13 cities in total) and then returning home (Supplementary Table B1). Lower values of *weight* introduce more randomness in choosing the next destination city (Equation 127). **(A)** Only a single Navigation Module B is used, so rather than restrict it to Equation (118) (which will yield each time a value of 8,131 miles per the nearest-neighbor algorithm), it is running Equation (120), i.e., small_plan(random=weight_distance). **(B)** The results are shown when the full set of Navigation Module Bs *n* = 1…1,023 are used. Here too, all modules use small_plan(random=weight_distance), but note that at higher values of the *weight* parameter, the CCA7 follows the nearest neighbor algorithm.

The data from Supplementary Table B2's left and right columns are plotted in Figures 13A, B, respectively. As can be seen from these figures, the lower values of the *weight* parameter in the single Navigation Module B variant of the architecture give significantly poorer results than in the multiple Navigation Module B's version (i.e., what is shown in Figure 9) of the architecture.

In the multiple Navigation Module B variant of the architecture (Figure 13B), it can be seen that *weights* of 1 and 2 give poorer results than the *weights* between 3 and 9 [*weights* over 9 automatically cause the nearest neighbor algorithm to be used (Equations 128, 129)].

### 5.2 Multiple Navigation Module Bs vs. original architecture (single Nav Module B)

Multiple runs to ensure statistical significance (or insignificance) of the data were done. As shown in Supplementary Table B3, 100 runs were observed for each variant of the architecture considered. The traveling salesperson problem data of Supplementary Table B1 was used.

Supplementary Table B3 in the middle column contains the shortest distances obtained with a version of the architecture using a single Navigation Module B utilizing the nearest neighbor algorithm. There is no randomness involved here, and as can be seen, the shortest distance of 8,131 miles was obtained in each of the 100 runs.

In Supplementary Table B3, in the right column, are the shortest distances obtained with a version of the architecture as shown in Figure 9 using 1,024 Navigation Module Bs. Navigation Module B *n* = 0 is reserved. Navigation Module *n* = 1 is used but set to use the nearest neighbor algorithm without randomness. Navigation Module Bs *n* = 2… 1,023 uses the nearest neighbor algorithm with the injection of random fluctuations with *weight* = 4 as per Equations (120–127). The mean shortest distance obtained was 7,432.2 miles, with a standard deviation of 141.8 miles; 1% of the runs yielded the shortest distance possible of 7,293 miles.

In Supplementary Table B3, in the left column, are the shortest distances obtained with a version of the architecture using a single Navigation Module B but with the injection of random fluctuations with a *weight* parameter value of 4 as per Equations (120–127). It does not reflect the CCA6 or the CCA7 architectures but was obtained to see that if running the single Navigation Module B variant (i.e., more similar to the CCA6 architecture) with random fluctuations would give better results. Note that the results show that the mean shortest distance was 9,965.5 miles with a standard deviation of 1,532.5 miles, i.e., worse results than obtained with the multiple Navigation Module Bs variant shown in the right column. No runs yielded the optimal path of 7,293 miles, with the shortest distance of the 100 runs being 7,647 miles.

**Figure 14:**
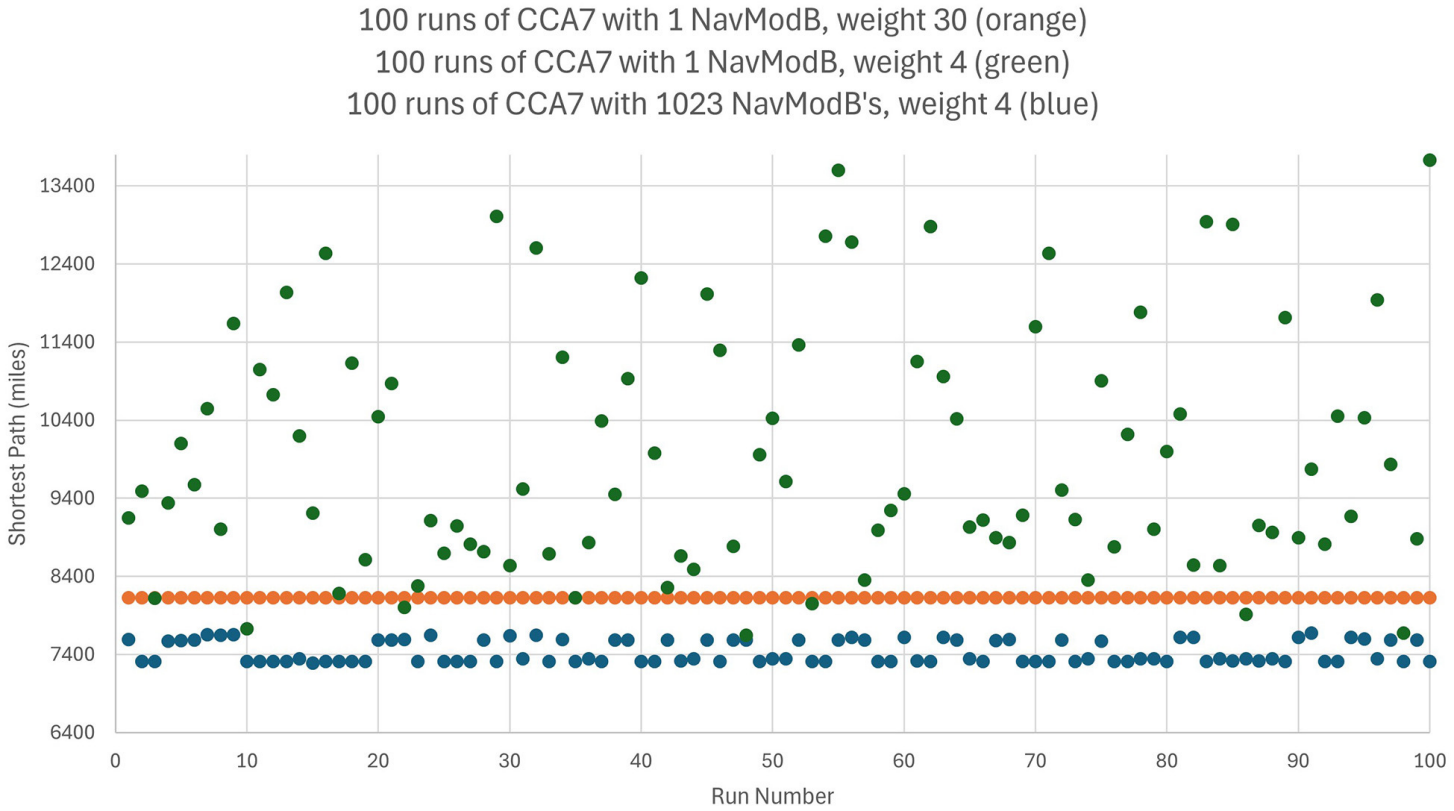
Simulation of the CCA7 robot navigating from a home city to 12 other cities (13 cities in total) and then returning home. One hundred simulated runs with the same dataset (Supplementary Table B1). Comparison of architecture with one Navigation Module B (with and without random fluctuations) versus with 1,023 Navigation Module Bs.

The three columns of Supplementary Table B3 are plotted in Figure 14. The green dots represent a version of the architecture using a single Navigation Module B but with the injection of random fluctuations with a *weight* parameter value of 4 as per Equations (120–127). As can be seen from the figure, this variant of the architecture gives the largest distances, i.e., the worst results.

In Figure 14, the constant line of orange dots represents a version of the architecture using a single Navigation Module B following the nearest-neighbor algorithm without any randomness injected. The blue dots represent the CCA7 version of the architecture shown in Figure 9, i.e., multiple Navigation Module Bs. As can be seen from this figure, these blue dots tend to give shorter distances, i.e., better results, than the orange dots.

To test for statistical significance between the results produced by the multiple Navigation Module Bs version of the architecture (right-most column of Supplementary Table B3) vs. the single Navigation Module B running the nearest neighbor algorithm (middle column of Supplementary Table B3), the probability of test results occurring due to random chance was calculated. A Welch's one-tailed *t*-test was performed, yielding a *p*-value of < 0.001.

Statistical significance of the results produced by the multiple Navigation Module Bs version of the architecture (right-most column of Supplementary Table B3) vs. the single Navigation Module B running the similar position-weighted algorithm (left-most column of Supplementary Table B3) was calculated via Welch's one-tailed *t*-test. Again, a small *p*-value was obtained and is recorded as *p* < 0.001 in Supplementary Table B3.

### 5.3 1K vs. 4K vs. 16K Navigation Module Bs

The CCA7 version of the architecture (Figure 9) arbitrarily allowed 1,024 Navigation Module Bs in the emergence of the duplication of navigation modules. However, any other number is possible, albeit with neurophysiological considerations in the case of a biological brain or engineering considerations in the case of an artificial implementation of the architecture.

Supplementary Table B4 compares 100 runs of the CCA7 version of the architecture operating on traveling salesperson problem data of Supplementary Table B1. All versions use a position-weighted method to inject randomness in the nearest neighbor algorithm, as per Equations (118–129). While in the left-most column of Supplementary Table B4, the architecture shown in Figure 9 and represented by Equations (118–129) is run, in the middle column is a similar architecture except for the utilization of 4,095 Navigation Module Bs, and in the right-most column is a similar architecture except for the utilization of 16,383 Navigation Module Bs.

Navigation Module B *n* = 0 is restricted for holding a copy of any instructions, and thus the availability of 1K-1, 4K-1, and 16K-1 Navigation Module Bs, which are rounded and simply referred to as “1K,” “4K,” or “16K,” respectively.

The CCA7 version with 4K Navigation Module Bs has a mean shortest distance of 7,309.1 miles (standard deviation 29.8 miles) vs. 7,432.2 miles (standard deviation 141.8 miles) for the 1K version; 27% of the runs of the 4K version yielded an optimal shortest distance path of 7293 miles vs. only 1% of the runs of the 1K version. As shown in Supplementary Table B4, Welch's 1-tail *t*-test was applied to this data and shows it is statistically significant at *p* < 0.001.

The CCA7 version with 16K Navigation Module Bs has a mean shortest distance of 7,296.8 miles (standard deviation 6.9 miles) vs. 7,309.1 miles (standard deviation 29.8 miles) for the 4K version; 67% of the runs of the 16K version yielded an optimal shortest distance path of 7,293 miles vs. 27% of the runs of the 4K version. As shown in Supplementary Table B4, Welch's 1-tail *t*-test was applied to this data and shows it is statistically significant at *p* < 0.001.

**Figure 15:**
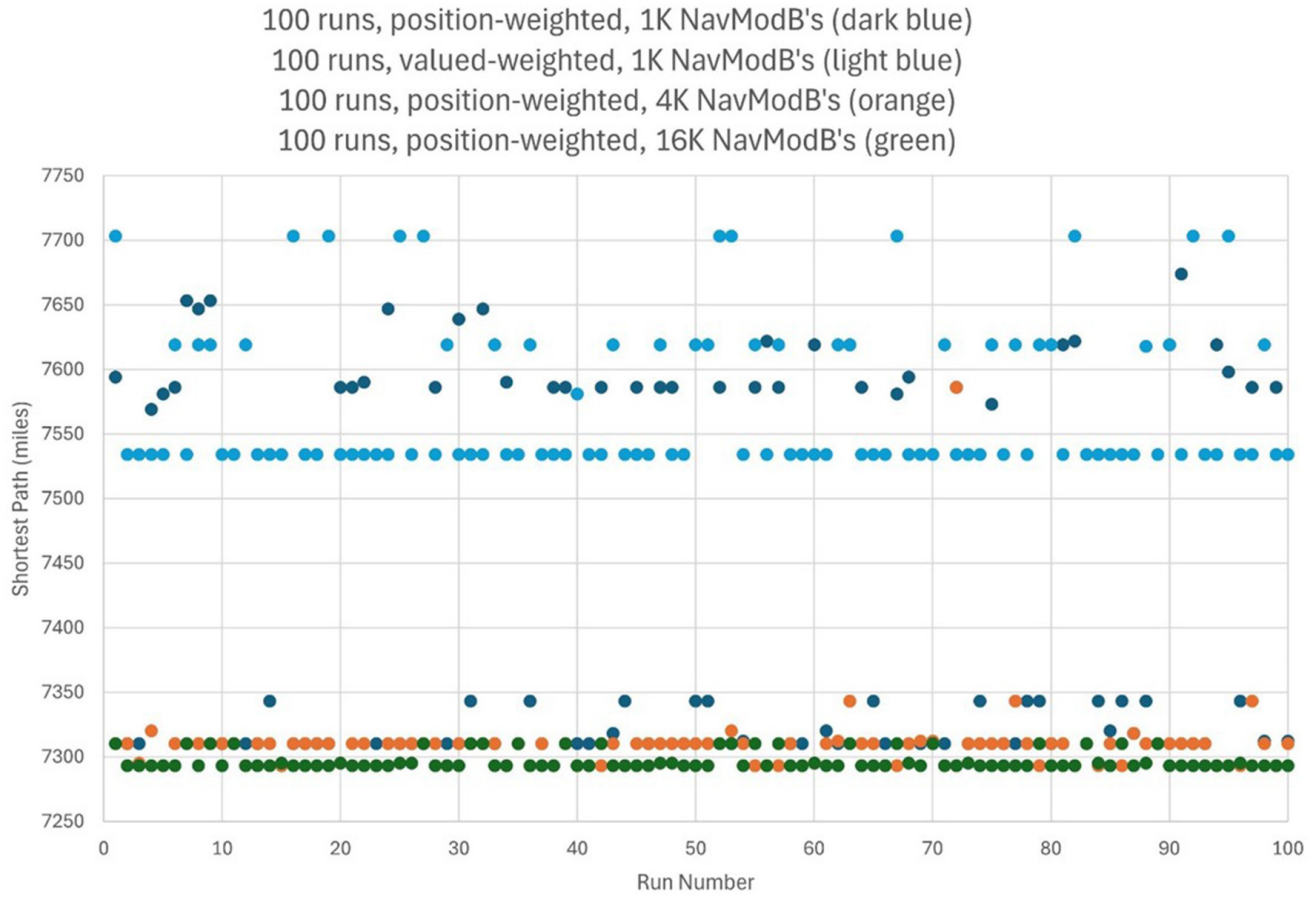
Simulation of the CCA7 robot navigating from a home city to 12 other cities (13 cities in total) and then returning home. One hundred simulated runs with the same dataset (Supplementary Table B1). Comparison of architecture with 1K, 4K, and 16K Navigation Module Bs, and comparison with 1K version running value-weighted algorithm (light blue dots).

The three columns of Supplementary Table B4 are plotted in Figure 15. The dark blue dots represent the 1K version of the architecture, the orange dots represent the 4K version of the architecture, and the green dots represent the 16K version of the architecture. As can be seen from this figure, while the 4K and 16K distances are close together, albeit with the 16K distances slightly smaller (although statistically significant, as noted above), they are significantly smaller than the results produced by the 1K version of the architecture.

### 5.4 Position weighted algorithm vs. value-weighted algorithm

As noted above, “position weighted” refers to Equations (124–127), which create a probability distribution of which location to choose next in deciding where to navigate to, simply in terms of which location is the nearest, the next-nearest, the third-next-nearest, and so on, without considering the absolute or relative values of the distances. In addition, as noted above, “valued weighted” refers to the modifications of these equations given by Section 4.2 such that the reciprocals of the difference of each distance value from the smallest value are calculated and normalized to create a probability distribution of which location to choose next in deciding where to navigate to.

Supplementary Table B5 compares 100 runs of the CCA7 version of the architecture operating on traveling salesperson problem data of Supplementary Table B1. The column on the left represents the usual CCA7 version of the architecture position-weighted method to inject randomness in the nearest neighbor algorithm, as per Equations (118–129). However, the column on the right represents a version of the CCA7 architecture using a valued weighted method to decide the probability of which location to navigate to at each decision point. Both architectures are using 1,023 Navigation Module Bs.

As shown in Supplementary Table B5, the usual position-weighted CCA7 version yields a mean shortest distance of 7,432.2 miles (standard deviation 141.8 miles) vs. 7,572.6 miles (standard deviation 58.0 miles) for the version using the valued-weighted method. The shortest distance (i.e., among the 100 runs performed) was 7,293 miles for the position-weighted CCA7 version vs. 7,534 miles for the value-weighted version of the architecture; 1% of the runs of the position-weighted architecture yielded the optimal shortest distance of 7,293, miles while 0% of the value-weighted architecture runs yielded an optimal shortest distance. A Welch's one-tailed t-test was performed, yielding a *p*-value of < 0.001.

The two columns of Supplementary Table B5 are also plotted in Figure 15. The dark blue dots represent the 1K version of the position-weighted version of the architecture, while the light blue dots represent the 1K version of the value-weighted version of the architecture.

## 6 Discussion

### 6.1 Interpretation of the results

In the CCA7 architecture using 1,023 Navigation Module Bs, Figure 13B shows that a weight value between 3 and 9 gives the shortest paths, i.e., the best results. Thus, in Equation (126), setting the *weight* value to 4 appears reasonable for use with the further experiments done in this study. Figure 13A was included for comparison, and it shows that in an architecture using only one Navigation Module B, the lowest weight values also give even poorer results.

Supplementary Table B3 and Figure 14 compare a CCA7 version of the architecture using a single Navigation Module B with a CCA7 version of the architecture using 1,023 Navigation Module Bs. The CCA7 version using 1,023 Navigation Module B's produced statistically significant (*p* < 0.001) shorter paths with a mean of 7,432.2 miles (standard deviation 141.8 miles) than the CCA7 version using only one Navigation Module B producing paths with a mean of 8,131.0 miles (standard deviation 0 miles since the nearest neighbor algorithm is followed the same each run). Suppose a CCA7 version using one Navigation Module B includes random fluctuations at each decision point, then the results are even worse with a shortest path mean of 9,965.5 miles (standard deviation 1,535.5 miles). Thus, the data support the premise of the CCA7 version of the architecture with multiple Navigation Module Bs, reflecting a hypothetical evolution of the previous architecture (intended to model the human brain, albeit very loosely in a functionalist fashion as per Lieto, 2021b) and performing better in this traveling salesperson problem, and potentially, all planning problems.

Many humans who look at the traveling salesperson problem distance values in Supplementary Table B1 can be shown how to perform the nearest neighbor algorithm, although without paper and a writing instrument, once the number of cities exceeds the limits of working memory, they often have trouble recalling which cities have already been navigated to, in addition to requiring good attention to find the smallest next number. In addition, even with good attention and good recall of which cities have been navigated to, humans cannot readily produce much better results from the numbers than such a nearest-neighbor solution. However, as MacGregor et al. (2004) note, humans can use a variety of cognitive processes to do better than expected on visual, Euclidean versions of the traveling salesperson problem. Nonetheless, as planning problems get larger or involve more dimensions, it becomes even harder for humans to obtain better results than is given by the CCA7 architecture.

Given that 1,023 Navigation Module Bs produce better results than a single Navigation Module B, it is interesting to consider the results of Supplementary Table B4 and Figure 15. The mean shortest distance for the traveling salesperson problem in Supplementary Table B1 is 7,432.2 miles (standard deviation 141.8 miles) for 1K Navigation Module Bs vs. 7,309.1 miles (standard deviation 29.8 miles) for 4K Navigation Module Bs vs. 7,296.8 miles (standard deviation 6.9 miles) for 16K Navigation Module Bs. Note that the theoretical shortest path of 7,293 miles is quite close to the mean results of the 4K, particularly the 16K architectures. Indeed, two-thirds of the runs done on the 16K version of the architecture yielded the theoretical shortest path. Thus, larger numbers of Navigation Module Bs allow better planning solutions as represented by this particular traveling salesperson problem. In larger planning problems, the 16K architecture will not as easily be able to find the optimal solution, but nonetheless, having additional Navigation Module Bs provides a larger solution space.

To avoid artificially creating arithmetic capabilities that the architecture did not possess, the utilization of the position weighting of the possible locations to navigate to next was discussed above. The results above of Supplementary Table B5 and Figure 15 compare a CCA7 version of the architecture using the position weighting method (given in Equations 124–127) vs. the value weighting method given in Section 4.2. In the value weighting method, the actual values of the distance to the next possible locations to navigate to are considered with each other. Supplementary Table B5 shows that the mean of the position weighting method gave a somewhat mean shortest distance of 7,432.2 miles (standard deviation 141.8 miles) vs. 7,534 miles (standard deviation 58.0 miles) for the value weighting method. This difference was statistically significant at *p* < 0.001. Thus, the position weighting method used by the CCA7 is reasonable compared to a simple value weighting method.

### 6.2 Parallel operation of different instinctive and learned primitives and their simulation

As noted above, Equation (116) indicates that the same instinctive primitive or the same learned primitive is initially applied to all of the Navigation Module Bs (Figure 9).

As discussed above, random fluctuations can be introduced in the different Navigation Module Bs to produce a variety of results to choose from. In the example shown above and simulated, i.e., the traveling salesperson problem, despite these random fluctuations, the same instinctive primitives were used by all the Navigation Module Bs, so it was not an issue [the instinctive primitive small_plan() did use a different argument in the *n* = 1 Navigation Module B, but it is automatically selected by the Navigation Module per Equation (118)].

However, what if a different type of problem is being operated on by the Navigation Module Bs such that there are also random fluctuations in the different modules, and as a result, at certain logical decision points, different instinctive or learned primitives are triggered, and must be obtained by the module? What if there are a million Navigation Module Bs, for example, all attempting to trigger and retrieve navigation maps at the same time from the instinctive primitives and learned primitives from the Causal Memory Module, Instinctive Primitives Module, and the Learned Primitives Module?

At present, the Python simulation of the CCA7 version of the architecture simply runs everything sequentially. Training of neural networks used by various modules is performed ahead of time, although, in the present version of the simulation, a pattern recognition library is used rather than the previous PyTorch machine learning Python library. The speed of modern computers is fast enough to handle simple simulations of the architecture, even with a slow language such as Python (albeit the data structure of the code is based on the Python library Numpy, which is written in efficient C code), so there has not been a need to pay attention to executing the simulation on parallel processors. In the present simulation, when all operations of a cognitive cycle have been completed, the next cognitive cycle starts without considering of how long the cognitive cycle actually took. Thus, even if a different type of problem, where the Navigation Module Bs must retrieve and write different navigation maps and primitives from different modules of the architecture, is simulated, the simulation will sequentially perform the operations and then consider the cognitive cycle completed. At present, there generally are no multiple comparisons between different modules within one cognitive cycle, which could cause problems with a sequential simulation.

There is a need for future work on the architecture to better specify the real-time parallel operation of the different Navigation Module Bs as well as to better simulate the architecture on parallel central processing units (CPUs)/graphics processing units (GPUs). A full consideration of parallel computing is beyond the scope of this article. However, the initial instinctive or learned primitive applied to all the Navigation Module B modules (i.e., *n* = 1… 1,023 of Figure 9) is effectively a single instruction stream, multiple data streams (SIMD) arrangement per Flynn's classification (Flynn, 1972). However, as processing occurs, this can essentially become a multiple instruction streams, multiple data streams (MIMD) arrangement. In future work, the CCA7 version of the architecture (i.e., Figure 9) and the associated equations/pseudocode describing it (i.e., Appendix A) need to be enhanced to better meet MIMD requirements. In particular, the multiple **TempMap** areas in each of the Navigation Module Bs are designed to hold not a “byte” of information but rather a “navigation map” of information, i.e., they can hold copies of instinctive primitives, learned primitives as well as portions of the Causal Memory Module. This resource can obviate many of the difficulties in implementing MIMD requirements.

### 6.3 Improved autonomic module control of the Navigation Module Bs and its simulation

The current Python simulation of the CCA7 version of the architecture does not simulate the Autonomic Module (Figure 9) in much detail. Given its brain-inspired origins, the Causal Cognitive Architecture has long had an Autonomic Module, as does the CCA7 (Figure 9; Schneider, 2021). Just as the autonomic nervous system in mammals plays a key role in maintaining homeostasis, so does the Autonomic Module in the Causal Cognitive Architecture, which involves itself with maintaining energy usage, heat production, sleep cycles, and the reliability of the architecture's physical embodiment.

Although sleep cycles do exist in the Python simulation of the Autonomic Module of the architecture, they are not very sophisticated. Either the architecture is on, or it is in a low-energy sleep-like state. Indeed, even though biological, mental activity is associated with region-specific increased energy consumption, the overall energy expenditure of the mammalian brain tends to be more constant than would be expected, whether the brain is problem-solving or idle but awake (Raichle and Gusnard, 2002). Indeed, when the mind is idle, a significant default mode network (DMN) of mammalian brain areas becomes active (Raichle, 2015). However, during sleep, the overall energy expenditure of the brain does indeed decrease (Dworak et al., 2010).

Thus, at present, the Autonomic Module in the Causal Cognitive Architecture, including the CCA7 version (Figure 9), simply has an awake/sleep functionality with regard to the energy expenditure of the architecture. However, in the CCA7 version of the architecture, there are now over a thousand Navigation Module Bs, and there could be, for example, in another implementation, millions of such Navigation Module Bs all working in parallel. The large numbers of Navigation Module Bs are not based on current mammalian including human brains, but instead on the hypothetical possibility that might arise in response to the question where in the Introduction section the question is asked: what if the evolution of the human brain were to continue as it has in the past, and given an environment for such evolution, as reflected in a model such as the Causal Cognitive Architecture.

A thousand, or especially a million, Navigation Module Bs operating all the time would seem to be wasteful with regard to the consumption of energy, as well as perhaps cause problems with regard to the dissipation of heat produced. In future work, there is a need for the Autonomic Module of Figure 9 to directly connect with the Navigation Module Bs so that it could regulate the activity of this ever-increasing portion of the architecture. Similarly, there is a need for principles of such regulation to be included in the equations and pseudocode of Appendix A. It is hypothesized that many planning situations and problems could be resolved with a limited number of Navigation Module Bs so that perhaps the entire repertoire of modules could be selectively activated by the Autonomic Module in response to repeated feedback loops of inability to yield a solution to a problem [e.g., Equation (95) above], or directly by triggering by an instinctive or learned primitive.

### 6.4 Enhanced intelligence from a brain-inspired cognitive architecture

As discussed above, the Causal Cognitive Architecture developed from the hypothesis that hundreds of millions of years ago, the navigation circuits in the amniotic ancestors of mammals duplicated many times to eventually form the neocortex. Thus, millions of neocortical minicolumns are functionally modeled in the architecture as millions of spatial maps, i.e., the “navigation maps” of the architecture. From this starting point, the properties of a cognitive architecture based on these navigation maps and inspired by the mammalian brain were investigated.

Without special feedback operations, a cognitive architecture based on navigation maps readily showed the reflexive and pre-causal behavior seen in most mammals (Schneider, 2021). Then modest changes, inspired by modest genetic changes from the last chimpanzee human common ancestor in the emergence of human cognitive abilities, were considered. Relatively modest changes were made, which simply allowed for enhanced feedback operations and the addition of extra instinctive primitives. With these modest changes, full causal decision-making emerged from the architecture (Schneider, 2022a). Further exploration revealed very small changes that allowed the emergence of full inductive analogical reasoning abilities (Schneider, 2023) and the very ready emergence of compositional comprehension and language in the CCA6 version of the architecture (Schneider, 2024).

While the CCA6 version of the architecture is very conceptual other than for a Python simulation, it seems to hold many of the features unique to human cognition—full causal reasoning, full analogical reasoning, near-full compositional (as opposed to combinatorial) language, and unfortunately the development of a vulnerability to psychosis (Schneider, 2020). In addition, note that navigation maps and consequential reasoning are fully grounded in the architecture (Schneider, 2023). In addition, note that the CCA6 is based on a cognitive architecture, which in its own right, forms the basis for an autonomous agent, i.e., tries to achieve goals by interactions with the environment (Paisner et al., 2014; Lieto et al., 2018).

In this study, it was considered whether further biologically plausible changes, as modest as possible, would allow significant improvements to the CCA6 architecture, such that super-human aspects of cognition would emerge. This study is particularly interested in enhancing core aspects of cognition in a human brain-inspired cognitive architecture. Integrating a calculator, for example, or perhaps a state-of-the-art large language model (LLM), would certainly boost the cognitive abilities of the architecture in a certain sense. Indeed, hybridization of the CCA6 architecture with other tools would seem to yield promising results. Liu et al. (2023) note that cognitive architectures and generative models have complementary strengths and weaknesses and discuss their fusion. These are valid topics for future consideration in the enhancement of Causal Cognitive Architecture. However, this study asks how modest, biologically plausible changes could allow core aspects of cognition to surpass normal human abilities.

Genetic and developmental mechanisms have been put forward for the duplication of mammalian brain circuits and their divergence to new functions (e.g., Rakic, 2009; Chakraborty and Jarvis, 2015). Thus, an increase in temporary memories and the increase in Navigation Module Bs as a biologically plausible theoretical modification of the architecture has been hypothesized above, resulting in the Causal Cognitive Architecture 7 (CCA7; Figure 9). The evolution of new instinctive primitives such as “small_plan()” may be more involved from a mechanistic point of view. While learned primitives can more readily acquire millions or billions of bytes of information, the genetic acquisition of an instinctive primitive has obvious resource and chance limitations [e.g., Weber et al. (2013) regarding the instinct for the burrowing habits of *Peromyscus* mice]. However, chimpanzees plan tool utilization (e.g., for use at termite nests), so some instinctual planning abilities may have been present for a long time, i.e., if present in the last chimpanzee human common ancestor, there is a longer period for such instincts to have evolved over time (Musgrave et al., 2023). Thus, the instinctive primitive “small_plan()” really should have been included in earlier versions of the Causal Cognitive Architecture and would have had enough time for any small changes to the version used in the CCA7.

As shown above, a large-scale duplication of the Navigation Module B circuits allows the architecture to have better planning abilities. This is readily apparent in Figure 14. The cognitive ability to imagine and plan for future events has long been considered a very advantageous aspect of cognition (e.g., Suddendorf and Corballis, 2007). Having super-human planning abilities, for example, such as the ability to simultaneously consider 1,023 (or 16,383) navigation routes (or analogous routes for other actions) as shown above, can allow the architecture to analyze and plan for its environment at a higher level than normally possible for the CCA6 with its single Navigation Module B, or a human.

Future work on the Causal Cognitive Architecture 7′s simulation includes enlarging its set of instinctive primitives and better educational experiences via (and better functioning of) the learned primitives system. In addition, with regard to planning, there is a myriad of other algorithms that are possible to consider rather than just introducing random fluctuations via the *weight* parameter. For example, the nearest-neighbor algorithm and even its modification with random fluctuations are susceptible to falling into a local optimum trap. For some planning problems, at the decision points, in addition to local information concerning which location is the closest, second closest, and so on, global information can be very advantageous to a more efficient shortest path. For example, in the ant colony optimization solution mentioned briefly above, accumulated knowledge from previous attempts to solve a route problem is used in addition to the local information at decision points. Further work is needed to apply these strategies to the CCA7′s instinctive and/or learned primitives. In addition, the effect of random fluctuations and planning in other areas besides the traveling salesperson problem needs to be considered. Once more of this work is done, it becomes more relevant to benchmark the architecture against other intelligent agents.

At present, the CCA7 is largely conceptual, its Python simulation notwithstanding, and cannot do useful work compared to a modern LLM-based chatbot, for example. However, as noted above, it seems to hold many features unique to human cognition—full causal reasoning, full analogical reasoning, near-full compositional language, and now planning. In addition, it is fully grounded and autonomous. Thus, in a conceptual sense, the CCA7 represents human-level artificial intelligence (HLAI) abilities. Given that the simultaneous multi-planning abilities of the CCA7 can be used for many cognitive processes at a level exceeding what humans are capable of, the CCA7 shows, in a conceptual sense, some sparks of superintelligence [the emphasis should be on “sparks” rather than “superintelligence,” with homage to Bostrom (2014) and Bubeck et al. (2023)]. As brain-inspired cognitive architectures such as the CCA7 become developed enough to realize their theoretical potential (or possibly fail at it), they should be considered as a viable alternative pathway toward the development of HLAI and then superintelligence, as well as giving insight into the emergence of natural human-level intelligence.

## Data availability statement

The original contributions presented in the study are included in the article/Supplementary material, further inquiries can be directed to the corresponding author.

## Author contributions

HS: Writing – original draft, Writing – review & editing.
