# Supplementary material for: The emergence of enhanced intelligence in a brain-inspired cognitive architecture
Source: Front Comput Neurosci. 2024 May 7;18:1367712. doi: 10.3389/fncom.2024.1367712 (PMC11231642; doi:10.3389/fncom.2024.1367712)
Supplement: Supplementary file 1 [file Data_Sheet_1.docx]

Supplementary Material

Paper: The Emergence of Enhanced Intelligence in a Brain Inspired Cognitive Architecture

Author: Howard Schneider

Corresponding Author: Howard Schneider

Date: April 26, 2024

# Appendix A: Formal Description of the Causal Cognitive Architecture 7 (CCA7)

To provide a more formal yet understandable and relevant description of the Causal Cognitive Architecture 7 (CCA7), the quasi-formalization is provided below. This formalization provides the basis for the Python simulations of the Causal Cognitive Architecture 7.

For simplicity, much of the same nomenclature adopted by the previous architecture (CCA6 – Schneider, 2024) is kept in the description below.

The CCA7 architecture (Figure 9) is described by the modified equations below. They are “modified” in the sense that many of the equations contain pseudocode. A pseudocode is a common language (e.g., English) description of the logic of a software routine (Kwon, 2014; Olsen, 2005). Note that all symbols and all pseudocode of all equations are explained in the text and in the associated tables. The use of a more abstracted pseudocode in the equations below largely provides a more understandable description of the architecture without sacrificing much accuracy.

Many of the properties of the CCA7 architecture, and the corresponding equations below, are similar to the prior CCA6 architecture. However, the planning and parallel operations are new, and previous equations have been modified where required, and new equations were added.

The equations below represent one cognitive cycle through the CCA7 architecture, although some of the backward flow of the feedback information is also described. Not every path or module of the architecture is described. For example, the equations do not describe flow of sensory data through the Autonomous Reflex Modules (Figure 1). In actual operation many cognitive cycles of the architecture occur over and over again each second.

Bold capitalized letters represent arrays, e.g., in equation (18) below **LNM** represents an array which acts as a navigation map. Vectors are represented in bolded italics (e.g., vector ***s*** in equation (9)). Many of the values change with time and are shown with the subscript *t*. For example, **S’***_σ,t_* (18) represents an array **S’***_σ_* with a value changing with time *t*. The subscript *t-1* represents the previous cognitive cycle and the subscript *t+1* represents the next cognitive cycle.

A.1 Input Sensory Vectors Shaping Modules

This section of Appendix A applies to the CCA5 (shown in Figure 1), the CCA6 (Figure 5) and the CCA7 (Figure 9) versions of the Causal Cognitive Architecture.

Figure 1 shows sensory inputs streaming into the Sensory Vectors Shaping Modules of the architecture. Sensory inputs for any sense are propagated into the architecture as a two-dimensional or three-dimensional spatial array of inputs. Spatial information means some sort of sensory information about a small volume of space which can be addressed and modeled as cell x,y,z. For example, for the visual sensory system, this spatial information would be visual sensory inputs (e.g., lines or no lines as a very simple example) observed at particular locations in the environment.

The details of signal processing, from the quantum level to the output produced by a transducer after possibly multi-layered signal processing, are largely abstracted away in this formalization. This does not diminish the importance of better signal processing. However, the architecture is concerned with whatever processed sensory inputs stream in, and that is what is considered here.

An array **S***_σ_* receives the sensory inputs of a sensory system *σ* every cognitive cycle, i.e., essentially changing with respect to time *t* (1 – 9). Vector ***s***(*t*) is processed by the Input Sensory Vectors Shaping Modules (via pseudocode Input_Sens_Shaping_Mods.normalize(), described below in Table AA1) into vector ***s’***(*t*) (10). This transformation ensures that each element of ***s’*** will be compatible with the dimensions used by the navigation maps in many of the modules of the CCA6 (11).

As Table A2 shows, processed and normalized sensory system arrays **S’***_σ,t_* leave this module compatible with the other data structures, i.e., the navigation maps, of the architecture. **S’***_σ,t_* are in arrays of dimensions m x n x o x p corresponding to the three spatial dimensions x,y,z and a fourth non-spatial dimension p (used in implementations of the architecture for the storage of segmentation data, i.e., defining objects in a scene, and metadata).

These equations remain largely unchanged in the CCA7 architecture from the prior CCA5 and CCA6 architectures.

S_1_ ∈ R*^m1^* ^x^ *^n1^* ^x^ *^o1^* (1)

S_1_*_,t_* = visual inputs*(t)* (2)

S_2_  ∈ R*^m2^* ^x^ *^n2^* ^x^ *^o2^* (3)

S_2_*_,t_* = auditory inputs*(t)* (4)

S_3_  ∈ R*^m3^* ^x^ *^n3^* ^x^ *^o3^* (5)

S_3_*_,t_* = olfactory inputs*(t)* (6)

*σ* = sensory system identification code ∈ N (7)

ϴ_σ = total number of sensory systems ∈ N (8)

*s*(*t*) = [ S_1_*_,t_*_,_ S_2_*_,t_*_,_ S_3_*_,t_*_, …,_ S*_ϴ_*___*_σ,t_*] (9)

***s’***(*t*) = Input_Sens_Shaping_Mods**.**normalize(***s***(*t*)) = [**S’**_1_*_,t_*_,_ **S’**_2_*_,t_*_,_ **S’**_3_*_,t_*_, …,_ **S’***_ϴ_*___*_σ,t_*] (10)

**S’***_σ,t_* ∈ R*^m^* ^x^ *^n^* ^x^ *^o x p^* (11)

| **S**_1_ ∈ R*^m1^* ^x^ *^n1^* ^x^ *^o1^* | -defining **S**_1_ to be an array of dimensions m1 x n1 x o1  -the form of our data structures is important; as will be seen below, most of the architecture utilizes a ‘navigation map’ data structure, i.e., arrays, of size *m* x *n* x *o x p*  -as noted in the text above, an array **S***_σ_* receives the sensory inputs of a sensory system *σ* every cognitive cycle; in this case array **S**_1_ receives the sensory inputs of sensory system 1, which are the visual sensory inputs  -as noted in the text above, the details of sensory perception, i.e., sensory signal processing, from the quantum level to the output produced by a transducer after possibly multi-layered signal processing, are largely abstracted away in this formalization. |
| --- | --- |
| **S**_1_*_,t_* | an array holding visual sensory inputs, which change with time |
| **S**_2_*_,t_* | an array holding auditory sensory inputs, which change with time |
| **S**_3_*_,t_* | -an array holding olfactory sensory inputs, which change with time  -for simplification implementing the olfactory sensory inputs similar to other sensory system, although this does not reflect actual mammalian neurophysiology |
| *σ* | =1 for visual, =2 for auditory, =3 for olfactory, other senses not used at present |
| ϴ_σ | total sensory systems which above would be 3 for the current simulation |
| ***s****(t)* | a vector holding all the sensory input arrays |
| Input_Sens_Shaping_Mods.normalize() | normalizes the sensory input arrays to the same dimensions used by the other navigation maps in the architecture |
| ***s’****(t)* | a vector holding all the normalized sensory input arrays, each now with dimensions m x n x o x p (which in the simulation in the paper are implemented as 6 x 6 x 6, corresponding to x,y,z axes of 6 x 6 x 6, and another dimension used for object segmentation data x 16) |
| **S’***_σ,t_* | normalized array (compatible with navigation maps in the other modules of the architecture) of sensory inputs of sensory system *σ* (which change with time) |

Table A1. Explanation of Symbols and Pseudocode in Equations (1) – (11)

| **Input:** | sensory inputs from sensory systems 1…ϴ_σ |
| --- | --- |
| **Output:** | ***s’****(t)* -- a vector holding all the normalized sensory input arrays **S’**_1_*_,t_* _…_ **S’**_ϴ_σ_*_,t_* |
| **Description:** | **-**Raw sensory inputs from the environment are normalized into a format compatible with navigation map data structure used by the other modules of the architecture.  -The current simulation of the architecture includes an experimental environment simulation module (i.e., simulates the sensory inputs).  -Applies to CCA5, CCA6 and CCA7 versions of the architecture |

Table A2. Summary of the Operations of the Input Sensory Vectors Shaping Modules per Equations (1) – (11)

A.2 The Input Sensory Vectors Association Modules

This section of Appendix A applies to the CCA5 (shown in Figure 1), the CCA6 (Figure 5) and the CCA7 (Figure 9) versions of the Causal Cognitive Architecture.

Figure 1 shows normalized sensory inputs for each sensory system from the Input Sensory Vectors Shaping Modules to the Input Sensory Vectors Association Modules. The normalized sensory input arrays **S’**_1_*_,t_* _…_ **S’**_ϴ_σ_*_,t_* are fed into corresponding modules (one module for each sensory system) of the Input Sensory Vectors Association Modules. Each module will map the sensory inputs it is receiving into a navigation map. This navigation map is called a “local navigation map” **LNM_(_***_σ_*_,_*_mapno_*_)_ (i.e., local navigation map **LNM** with address *mapno* in sensory system *σ*) (14). Each cell in three spatial dimensions in **LNM**_(_*_σ_*_,_ *_mapno_*_)_ can represent the full contents of each cell, i.e., all the features, procedures, and link addresses associated with a cell. As (14) shows there is also a non-spatial dimension p which is used in implementations to store various non-spatial information.

***all_maps****_σ,t_* is a vector holding all the local navigation maps in the *σ* sensory system Input Sensory Vectors Association Module (15). For example, ***all_maps***_1_*_,t_* represents all the stored local navigation maps in the visual Input Sensory Vectors Association Module (which as noted above is *σ* **=** 1).

As noted above **S’_1_*_,t_*** is an array of the visual processed inputs (i.e., *σ* **=** 1). **S’**_2_*_,t_* are the auditory processed inputs since *σ* = 2, and so on. The visual processed inputs **S’_1_*_,t_*** should be matched against ***all_maps***_1_*_,t_* , i.e., against all the other visual local navigation maps stored in the visual Input Sensory Vectors Association Module. The auditory, olfactory and any other sensory inputs should be matched against the respective local area maps stored in a particular Input Sensory Vectors Association Module.

In (18) Input_Assocn_Mod***_σ_*.**match_best_local_navmap is pseudocode that matches an incoming sensory array *σ* (e.g., if *σ*=1 then **S’**_1_*_,t_* is an array of the visual processed inputs) against the respective stored local navigation maps ***all_maps****_σ,t_* (e.g., if *σ*=1 then ***all_maps****_1,t_* holds all the stored local navigation maps in the visual Input Sensory Vectors Association Module). **LNM_(_*_σ_*_,_ *_ϓ ,t_*_)_** represents the local navigation map **LNM** in sensory system *σ* with a *mapno* of *ϓ* which is the best match of **S’***_σ,t_*. For example, if sensory inputs array **S’**_1_*_,t_* best matches to *mapno* 3456 (as an example) in the local navigation maps stored in the visual Input Sensory Vectors Association Module, then **LNM**_(1,_ *_ϓ ,t_*_)_ would be local navigation map 3456 in the visual Input Sensory Vectors Association Module.

Above it was noted that the Causal Cognitive Architecture makes heavy use of feedback pathways—states of a downstream module can influence the recognition and processing of more upstream sensory inputs. Thus, the previous cognitive cycle’s Working Navigation Map **WNM’**_t-1_ (the navigation map in the Navigation Module A in Figure 9) and on which the Navigation Module A can perform operations on) is used in the pseudocode method (18) in deciding which is a best match.

These equations remain largely unchanged in the CCA6 and CCA7 architectures from the prior CCA5 architecture, although there is the allowance of multiple navigation modules in performing operations.

*mapno*  = map identification code ∈ N (12)

ϴ = total number of used local navigation maps in a sensory system *σ* ∈ N (13)

**LNM**_(_*_σ_*_,_*_mapno_*_)_ ∈ R*^m^*^x^*^n^*^x^*^oxp^* (14)

*all_maps_σ,t_* = [LNM_(_*_σ_*_,1_*_,t_*_)_, LNM_(_*_σ_*_,2_*_,t_*_)_, LNM_(_*_σ_*_,3_*_,t_*_)_, …, LNM_(_*_σ_*_, ϴ, t)_] (15)

*ϓ*  = map number of best matching map in a given set of navigation maps ∈ *mapno* (16)

**WNM’** = ∈ R*^m^*^x^*^n^*^x^*^oxp^* (17 and defined again below)

**LNM**_(_*_σ_*_,_ *_ϓ ,t_*_)_ **=** Input_Assocn_Mod***_σ_*.**match_best_local_navmap( **S’***_σ,t_*, ***all_maps****_σ,t_*, **WNM’**_t-1_) (18)

| **LNM**_(_*_σ_*_,_*_mapno_*_)_ | a LNM or “local navigation map” which is a navigation map (i.e., an array of x,y,z dimensions m x n x o, which in the simulation in the paper are 6 x 6 x 6, corresponding to x,y,z axes of 6 x 6 x 6) that is held in the Input Sensory Vectors Association Module *σ* (e.g., if *σ* = 1, then that would be the module receiving the visual sensory inputs) and is map number *mapno* (there may be millions of other local navigation maps stored in that Input Sensory Vectors Association Module *σ*) |
| --- | --- |
| ***all_maps****_σ,t_* | a vector holding all the LNMs in module *σ* (i.e., starting with the first LNM and going to the last utilized LNM_ϴ_)  e.g., ***all_maps****_1,t_* would be all the LNMs in the Input Sensory Vectors Association Module 1 which are all the local navigation maps created and stored from the visual sensory inputs  -there are new sensory inputs each cognitive cycle, and thus new local navigation maps will be produced, and thus there is a t subscript representing a change with time |
| **WNM’** | a WNM is a “Working Navigation Map”  it is a navigation map which for the moment is in the Navigation Module (Figure 1) and on which the Navigation Module can perform operations on  As will defined below, operations can be performed by Navigation Module A or Navigation Module B, where are referred to as ‘Navigation Module’ in this section |
| Input_Assocn_Mod*_σ_***.**match_best_local_navmap(**S’***_σ,t_*, ***all_maps****_σ,t_*, **WNM’**_t-1_) | pseudocode for an algorithm which will match the *σ* sensory inputs (e.g., if *σ* = 1 then it would be visual sensory inputs) against all the local navigation maps stored in the *σ* module of the Input Sensory Vectors Association Modules (Figure 1) and returns the best matched local navigation map **LNM** for that sensory module  -note that the results of the Navigation Module in the previous cycle as represented by previous cognitive cycle’s Working Navigation Map **WNM’**_t-1_ are considered in the determining the best match |
| *ϓ* | represents a map number of a navigation map in an Input Sensory Vectors Association Module |
| **LNM**_(_*_σ_*_,_ *_ϓ ,t_*_)_ | the local navigation map **LNM** stored in the Input Sensory Vectors Association Module *σ* with map number *ϓ* which best matches in the incoming *σ* sensory inputs (e.g., if *σ* = 1 then it would be visual sensory inputs)  e.g. if the visual sensory inputs best match the local navigation map #3456 in the visual module of the Input Sensory Vectors Association Modules, then at that moment **LNM**_(1,_ *_ϓ ,t_*_)_ would be navigation map #3456 in the visual module |

Table A3. Explanation of Symbols and Pseudocode in Equations (12) – (18)

At this point, in every sensory module in the Input Sensory Vectors Association Modules (Figure 1), there is a best matching **LNM_(_***_σ_*_,_ *_ϓ ,t_*_)_ (“local navigation map” since these navigation maps are stored locally in the sensory module rather than being stored in the Causal Memory Module attached to the Navigation Module, as seen in Figure 1). The next operation is to update the best matching local navigation map **LNM**_(_*_σ_*_,_ *_ϓ ,t_*_)_ with the actual sensory inputs **S’***_σ,t_* (21), creating an updated best matching navigation map **LNM’**_(_*_σ_*_,_ *_ϓ ,t_*_)_ which is renamed (for simulation compatibility issues) to **LNM**_(_*_σ_*_,_ *_ϓ ,t_*_)_ again.

If too many differences exist between the actual sensory inputs **S’***_σ,t_* and the best matching navigation map **LNM**_(_*_σ_*_,ϓ_*_,t_*_)_, then instead of updating the matched navigation map **LNM**_(_*_σ_*_,_ *_ϓ ,t_*_)_, a new local navigation map **LNM**_(_*_σ_*_,_*_new_map,t_*_)_ is created and updated with the actual sensory inputs **S’***_σ,t_* forming an updated best matching navigation map **LNM’**_(_*_σ_*_,_ *_ϓ ,t_*_)_ (22), which is renamed (for simulation compatibility issues) to **LNM**_(_*_σ_*_,_ *_ϓ ,t_*_)_ again.

In each sensory system Input Sensory Vectors Association Module the updated local navigation map (or newly created and updated one) is stored in the Input Sensory Vectors Association Module *σ*, and in future cognitive cycles, sensory inputs will be matched against it and the other local navigation maps stored there.

Vector ***lnm****_t_* represents the best-matching and updated local navigation maps **LNM**_(_*_σ_*_, ϓ, t )_ of all the different sensory modules of the Input Sensory Vectors Association Modules (23).

These equations remain largely unchanged in the CCA6 architecture from the prior CCA5 architecture, although there is the allowance of multiple navigation modules in performing operations.

h **=** number of differences allowed to be copied onto existing map ∈ R (19)

*new_map* *=* map number of new local navigation map added to current sensory system *σ* ∈ *mapno* (20)

| Input_Assocn_Mod*_σ_*.differences (**S’***_σ,t_* , **LNM_(_***_σ_*_,_ *_ϓ ,t_*_)_) **| ≤**h **,**

⇒ **LNM**_(_*_σ_*_,_ *_ϓ ,t_*_)_ **= LNM’**_(_*_σ_*_,_ *_ϓ ,t_*_)_ **= LNM**_(_*_σ_*_,_ *_ϓ ,t_*_)_ ∪ **S’***_σ,t_* (21)

| Input_Assocn_Mod*_σ_*.differences (**S’*_σ,t_*** , **LNM**_(_*_σ_*_,_ *_ϓ ,t_*_)_) **| >** h **,**

⇒ **LNM**_(_*_σ_*_,_ *_ϓ ,t_*_)_ **= LNM’**_(_*_σ_*_,_ *_ϓ ,t_*_)_ **= LNM**_(_*_σ_*_,_ *_new_map,t_*_)_ ∪ **S’***_σ,t_* (22)

*lnm_t_* = [LNM_(1,_ *_ϓ ,t_*_)_, LNM_(2,_ *_ϓ ,t_*_)_, LNM_(3,_ *_ϓ ,t_*_)_, …, LNM_(_*_ϴ_σ_*_,_ *_ϓ ,t_*_)_] (23)

| h | the number of differences allowed to be copied onto existing map – if there are too many differences between the best matching local navigation map retrieved from the sensory system’s Input Sensory Vectors Association Module (Figure 1) and the actual input sensory signal (i.e., **S’***_σ,t_* ) then rather than copying the information from input sensory array onto the best matching local navigation map, and simply makes **S’***_σ,t_* into a new local navigation map |
| --- | --- |
| *new_map* | the *mapno* of an empty local navigation map in a sensory system’s Input Sensory Vectors Association Module used when making **S’***_σ,t_* into a new local navigation map |
| Input_Assocn_Mod*_σ_*.differences | -pseudocode for an algorithm that calculates the differences between two navigation maps  -used in (21) and (22) to calculate the differences between the input sensory signal **S’***_σ,t_* and the retrieved best matching local navigation map **LNM_(_***_σ_*_,_ *_ϓ ,t_*_)_ |
| **LNM**_(_*_σ_*_,_ *_ϓ ,t_*_)_ ∪ **S’***_σ,t_*  **🡪 LNM’ 🡪 LNM** | update **LNM**_(_*_σ_*_,_ *_ϓ ,t_*_)_ with any new information in input sensory signal **S’***_σ,t_* (i.e., copy **S’***_σ,t_* onto **LNM_(_***_σ_*_,_ *_ϓ ,t_*_)_ ) thereby creating an updated **LNM’**_(_*_σ_*_,_ *_ϓ ,t_*_)_  (for simulation compatibility issues renaming **LNM’**_(_*_σ_*_,_ *_ϓ ,t_*_)_ to **LNM**_(_*_σ_*_,_ *_ϓ ,t_*_)_ again) |
| **LNM**_(_*_σ_*_,_*_new_map,t_*_)_∪ **S’***_σ,t_*  **🡪 LNM’** **🡪 LNM** | copy **S’***_σ,t_* onto an empty **LNM**_(_*_σ_*_,_ *_new_map_* *_,t_*_)_ thereby creating an updated **LNM’**_(_*_σ_*_,_ *_ϓ ,t_*_)_  (for simulation compatibility issues renaming **LNM’**_(_*_σ_*_,_ *_ϓ ,t_*_)_ to **LNM**_(_*_σ_*_,_ *_ϓ ,t_*_)_ again) |
| ***lnm****_t_* | i.e., a vector holding [**LNM**_(1,_ *_ϓ ,t_*_)_, **LNM**_(2,_ *_ϓ ,t_*_)_, **LNM**_(3,_ *_ϓ ,t_*_)_, …, **LNM**_(_*_ϴ_σ_*_,_ *_ϓ ,t_*_)_] -- the local navigation maps **LNM** of each sensory system which best matches the corresponding sensory input array **S’***_σ,t_* and then are updated to **LNM’** with the actual sensory information conveyed by **S’***_σ,t_* (for simulation compatibility issues renaming **LNM’**_(_*_σ_*_,_ *_ϓ ,t_*_)_ to **LNM**_(_*_σ_*_,_ *_ϓ ,t_*_)_ again) |

Table A4. Explanation of Symbols and Pseudocode in Equations (19) – (23)

| **Input:** | ***s’****(t)* -- a vector holding all the normalized sensory input arrays **S’**_1_*_,t_* _…_ **S’**_ϴ__*_σ,t_* |
| --- | --- |
| **Output:** | ***lnm_t_*** = [**LNM**_(1,_ *_ϓ ,t_*_)_, **LNM**_(2,_ *_ϓ ,t_*_)_, **LNM**_(3,_ *_ϓ ,t_*_)_, …, **LNM**_(_*_ϴ_σ_*_,_ *_ϓ ,t_*_)_]  The local navigation maps **LNM** of each sensory system which best match the corresponding sensory input array **S’***_σ,t_* , and then are updated to **LNM’** with the actual sensory information conveyed by **S’***_σ,t_* (for simulation compatibility issues renaming **LNM’**_(_*_σ_*_,_ *_ϓ ,t_*_)_ to **LNM**_(_*_σ_*_,_ *_ϓ ,t_*_)_ again) |
| **Description:** | **-**The normalized sensory input arrays are best matched with stored navigation maps in each sensory system (called a local navigation map **LNM**).  -The best matched **LNM**s in each sensory system are then updated with the actual sensory information from the sensory input for that sensory system.  -The updated best matched **LNM** is stored locally in that Input Sensory Vectors Shaping Module for matching against future sensory inputs.  -The updated best matched **LNM** is output from this module; the best matched **LNM**s from each Input Sensory Vectors Shaping Module together make up vector ***lnm****_t_* –the effective output.  -Applies to CCA5, CCA6 and CCA7 versions of the architecture |

Table A5. Summary of the Operation of the Input Sensory Vectors Associations Modules per Equations (12) – (23)

A.3 Data Structures in the CCA6

This section of Appendix A applies to the CCA5 (shown in Figure 1), the CCA6 (Figure 5) and the CCA7 (Figure 9) versions of the Causal Cognitive Architecture.

This section reviews a number of data structures used in the CCA6 architecture, most of them being compatible with the navigation map data structure. Equation (14) defines the local navigation map **LNM** as an array of four (or more) dimensions. There are three spatial dimensions (m,n,o in the definition representing x,y,z) as well as an extra dimension p for non-spatial information such storing which features belong to which objects, for storing meta-data, and so on.

Local navigation maps (i.e., stored locally in each sensory Input Sensory Vectors Association Module) were defined above in (14). Multisensory navigation maps **NM**, instinctive primitive navigation maps **IPM**, and learned primitive navigation maps **LPM** are similarly defined in (24). The multisensory navigation maps **NM** are stored in the Causal Memory Module (Figure 1) and contain visual, auditory, olfactory, etc. sensory features unlike the local navigation maps which have features from only one sensory system. The instinctive primitive navigation maps **IPM** and the learned primitive navigation maps **LPM** have the same dimensional structure as other navigation maps, but they contain largely only procedures to perform on other navigation maps.

Equation (26) defines ***all_LNMs_t_*** as holding all the local navigation maps **LNM** in all the different Input Sensory Vectors Association Modules. Recall from above that ***all_maps****_σ,t_* holds all the local navigation maps **LNM** within a *given* Input Sensory Vectors Association Module. In (27) ***all_NMs****_t_* are defined as holding all the multisensory navigation maps **NM** in the Causal Memory Module (Figure 1). In (28) ***all_IPMs****_t_* are defined as holding all the instinctive primitive navigation maps **IPM** (or “instinctive primitives”—the procedures that are included with the architecture) in the Instinctive Primitives Module (Figure 1). In (29) ***all_LPMs****_t_* are defined as holding all the learned primitive navigation maps **LPM** (or “learned primitives”—the procedures that are learned by the architecture) in the Learned Primitives Module (Figure 1). And in (30) ***all_navmaps****_t_* are defined as holding all of these preceding navigation maps, i.e., [***all_LNMs****_t_,* ***all_NMs****_t_,* ***all_IPMs****_t_,* ***all_LPMs****_t_*] . (The vector ***all_navmaps****_t_* does not hold the entirety of navigation maps in the CCA6 architecture as there are a number of other specialized navigation maps, particularly in the Sequential/Error Correcting Module.)

In (31–33) an addressing protocol is defined to address any particular cell within any particular navigation map within ***all_navmaps****_t_* . For example, ***χ***_modcode=*Causal_Memory_Mod*,_ *_mapno_*_=3456,x=2,y=3,z=4_ is cell x=2,y=3,z=4 in map number 3456 in the Causal Memory Module.

In (34) a “*feature*” is defined as some arbitrary real number representing a feature modality and value. For example, *feature*_3,(_***_χ_***_modcode=*Causal_Memory_Mod*,_ *_mapno_*_=3456, x=2,y=3,z=4)_ would be *feature* number 3 in the cell x=2,y=3,z=4 in map number 3456 in the Causal Memory Module. Its value, for example, could represent a visual line. In (35) similarly a “*procedure*” is defined as some arbitrary real number representing a procedure modality and value. For example, *procedure*_3,(_***_χ_***_modcode=*Causal_Memory_Mod*,_ *_mapno_*_=3456, x=2,y=3,z=4)_ would be *procedure* number 3 in the cell x=2,y=3,z=4 in map number 3456 in the Causal Memory Module. Its value, for example, could represent a *procedure* (and required sub-procedures too; the value can have an unlimited number of digits) to move forward. In (36) a ***linkaddress*** is defined as ***χ’***, i.e., pointing to ***χ’*** which is some cell location in some navigation map in the architecture. The linkaddress provides a link between one cell in one map to another cell, possibly in the same map but often in a different navigation map. (Note: For stylistic reasons, in some places in the text “*features”* may be written which should be taken as the same variable as “*feature*”.)

In (38) the equation defines ***cellfeatures_χ_****_,t_* as all the features within a given cell ***χ*** *.* In (39) the equation defines ***cellprocedures_χ_****_,t_* as all the procedures within a given cell ***χ*** *.* In (40) the equation defines ***linkaddresses_χ_****_,t_* as all the linkaddresses within a given cell ***χ***. In (41) the equation defines ***cellvalues_χ_*** as all the values—the features, the procedures, the link addresses—held by a cell of a navigation map at location ***χ****.* The numbers in each cell can represent any collection of low-level sensory features, higher-level sensory features, procedures, and links.

As shown above, ***all_navmaps*** represents all the navigation maps in the architecture. In (41) it is seen that the value of a cell in one of the navigation maps in ***all_navmaps*** are its ***cellvalues_χ_****_,t_* _,_ i.e., the features, the procedures and the linkaddresses that the cell contains. Equation (42) shows that the pseudocode link(***χ****,t*) will return all the links that a cell at address ***χ*** contains.

These equations remain largely unchanged in the CCA6 and CCA7 architectures from the prior CCA5 architecture.

**NM***_mapno_* ∈ R*^m^*^x^*^n^*^x^*^oxp^*, **IPM***_mapno_* ∈ R*^m^*^x^*^n^*^x^*^oxp^*, **LPM***_mapno_* ∈ R*^m^*^x^*^n^*^x^*^oxp^* (24)

ϴ_NM = total used NM’s ∈ N, ϴ_IPM = total used IPM’s ∈ N, ϴ_LPM = total used LPM’s ∈ N (25)

*all_LNMs_t_* *=* [*all_maps_1,t_, all_maps_2,t_, all_maps_3,t_, …, all_maps*_ϴ_σ_*_,t_*] (26)

*all_NMs_t_* *=* [NM_1_*_,t_*, NM_2_*_,t_*, NM_3_*_,t_*, …, NM_ϴ_NM_*_,t_*] (27)

*all_IPMs_t_* *=* [IPM_1_*_,t_*, IPM_2_*_,t_*, IPM_3_*_,t_*, …, IPM_ϴ_IPM_*_,t_*] (28)

*all_LPMs_t_* *=* [LPM_1_*_,t_*, LPM_2_*_,t_*, LPM_3_*_,t_*, …, LPM_ϴ_LPM_*_,t_*] (29)

*all_navmaps_t_* *=* [*all_LNMs_t_, all_NMs_t_, all_IPMs_t_, all_LPMs_t_*] (30)

*modcode* = module identification code ∈ N (31)

*mapcode* = [*modcode, mapno*] (32)

*χ* = [*mapcode, x, y, z*] (33)

*feature* ∈ R (34)

*procedure* ∈ R (35)

*linkaddress* *χ’* ∈ *χ* (36)

Ф_*feature* = last *feature* contained by a cell, Ф_*procedure* = last *procedure* contained by a cell,
 Ф_χ = last χ (i.e., address) contained by a cell (37)

*cellfeatures_χ,t_ =* [*feature_1,t_*, *feature_2,t_*, *feature_3,t_*, …, *feature*_Ф_feature_*_,t_*] (38)

*cellprocedures_χ,t_ =* [*procedure_1,t_*, *procedure_2,t_*, *procedure_3,t_*, …, *procedure*_Ф__*_procedure,t_*] (39)

*linkaddresses_χ,t_ =* [*χ_1,t,_ χ_2,t,_ χ_3,t,_ …*, *χ*_Ф_χ_*_,t_*] (40)

*cellvalues_χ,t_ =* [*cellfeatures_χ,t_*, *cellprocedures_χ,t_*, *linkaddresses_χ,t_*] (41)

*cellvalues_χ,t_* = *all_navmaps_χ,t_* (42)

*linkaddresses_χ,t_* = link(*χ,t*) (43)

| **LNM** | local navigation map – one sensory system maps to any local navigation map  different set of LNMs for each sensory system  each set of LNMs stored in that particular sensory Input Sensory Vectors Association Module (Figure 1)  array structure of dimensions m,n,o,p (x,y,z and non-spatial p dimension) |
| --- | --- |
| **NM** | multisensory navigation map – different sensory system features can be written to this map  stored in the Causal Memory Module (Figure 1)  array structure of dimensions m,n,o,p (x,y,z and non-spatial p dimension) |
| **IPM** | -instinctive primitive navigation map – “instinctive primitive”  -primitives are procedures to perform on cells of other navigation maps  -primitives are navigation maps mainly filled with procedures in their cells, but they can store features and linkaddresses in their cells as well  -these primitives come with the architecture  -stored in the Instinctive Primitives Module (Figure 1)  -array structure of dimensions m,n,o,p (x,y,z and non-spatial p dimension) |
| **LPM** | -learned primitive navigation map – “learned primitive”  -primitives are procedures to perform on cells of other navigation maps  -primitives are navigation maps mainly filled with procedures in their cells, but they can store features and linkaddresses in their cells as well  -these primitives are learned by the architecture  -stored in the Learned Primitives Module (Figure 1)  -array structure of dimensions m,n,o,p (x,y,z and non-spatial p dimension) |
| ***all_maps****_1_* | vector of all of the LNMs in *σ*=1, i.e., visual Input Sensory Vectors Association Module (*note: ‘t’ which indicates changes with time has been removed here and following entries for simplification purposes*)  (***all_maps****_2_* i.e., *σ*=2, is auditory module, and so on) |
| ***all_LNMs*** | vector of all of the LNMs in all of the Input Sensory Vectors Association Modules |
| ***all_NMs*** | vector of all of the NM’s in the Causal Memory Module |
| ***all_IPMs*** | vector of all of the IPM’s in the Instinctive Primitives Module |
| ***all_LPMs*** | vector of all of the LPM’s in the Learned Primitives Module |
| ***all_navmaps*** | vector of all of the LNMs, NM’s, IPM’s, LPM’s in the architecture |
| ***mapcode*** | [module identification code, map number]  points to a particular navigation map among all the LNMs, NM’s, IPM’s, and LPM’s in the architecture |
| ***χ*** | [mapcode, x, y, z]  points to particular cell (x,y,z) in a particular navigation map ([module identification code, map number]) |
| *feature*  *features* | arbitrary real number representing a feature modality and value within a cell ***χ***  (Note: For stylistic reasons, in some places the text may write “*features”* which should be taken as the same variable as “*feature*”.) |
| *procedure* | arbitrary real number representing a procedure modality and value within a cell ***χ*** |
| linkaddress | address within a cell ***χ*** pointing to another cell (possibly in another navigation map) ***χ’*** |
| ***cellfeatures_χ_*** | vector of all the features within a cell ***χ*** |
| ***cellprocedures_χ_*** | vector of all the procedures within a cell ***χ*** |
| ***linkaddresses_χ_*** | vector of all the linkaddress within a cell ***χ*** |
| ***cellvalues_χ_*** | vector of all of the features, procedures and linkaddresses within a cell ***χ*** |
| ***all_navmaps_χ_*** | vector of all of the features, procedures and linkaddresses within a cell ***χ***  note that the value of some cell ***χ*** in some cell in some navigation map (i.e., within the collection of  ***all_navmaps***) = ***cellvalues_χ_*** |
| link(***χ****,t*) | pseudocode that returns all the linkaddresses within a cell ***χ*** , i.e., same value as ***linkaddresses_χ_*** |

Table A6. Explanation of Symbols and Pseudocode in Equations (24) – (43)

| **Input:** | *not applicable – definitions of data structures in this section* |
| --- | --- |
| **Output:** | *not applicable – definitions of data structures in this section* |
| **Description:** | -In this section some of the key data structures utilized by the architecture are discussed.  -These data structures are largely based on the “navigation map” which maps spatial features (e.g., are there pixels representing ground at this x,y,z coordinate?), potential procedures (e.g., do something with the data in this or other cells of the navigation map), and link addresses (e.g., possibly go to the cell in possibly another navigation map specified by the link address) at a particular x,y,z coordinate (“cell” or “cube”).  -The architecture in conjunction with the navigation map data structure allows a solution to the classical binding problem.  -Applies to CCA5, CCA6 and CCA7 versions of the architecture |

Table A7. Summary of the Operation of Equations (24) – (43)

A.4 The Sequential/Error Correcting Module

This section of Appendix A applies to the CCA5 (shown in Figure 1), the CCA6 (Figure 5) and the CCA7 (Figure 9) versions of the Causal Cognitive Architecture.

Consider the sensory inputs through the CCA6 architecture as they become temporally bound. The Sequential/Error Correcting Module plays a key role in temporally binding the sensory inputs.

Normalized sensory input arrays **S’***_σ,t_* represented by ***s’****(t)* (from the output of the Input Sensory Vectors Shaping Modules (10, 11)) feed into the Sequential/Error Correcting Module. After processing these signals the Sequential/Error Correcting Module sends a “motion prediction vector” to the Navigation Module. The motion prediction vector allows changes in an object(s) in a navigation map to be represented much as other spatial features on a navigation map.

In (44) ***s’_series****_t_* is a time series of the input sensory vector ***s’****(t)*. In (45) and (46) the visual and auditory sensory times series are extracted from ***s’_series****_t_* and stored respectively as ***visual_series****_t_* and ***auditory_series****_t_*. Although it is possible to create motion prediction vectors for all the senses, in the current simulation this is only done so for the visual and auditory sensory systems.

In (47) the pseudocode Sequential_Mod.visual_match()matches ***visual_series****_t_* with visual time series stored in the Sequential/Error Correcting Module. If there is a reasonable match with few differences, then the matched time series will be updated with the new information. If there is no close enough match, then a new times series will be stored in the Sequential/Error Correcting Module. A motion prediction vector  ***visual_motion****_t_* is then computed from visual time series data (47). A similar process occurs in computing the motion prediction vector ***auditory_motion****_t_* (48). These motion prediction vectors are then stored much like any spatial feature in a navigation map called the Vector Navigation Map **VNM’’***_t_* (50a, 50b). **VNM’’***_t_* is then propagated to the Navigation Module complex (Figure 1).

In (51) the pseudocode Sequential_Mod.auditory_match_process() extracts sound patterns from ***auditory_series****_t_,* and stores these patterns spatially in a navigation map **AVNM***_t_*. Then **AVNM***_t_* is propagated to the Navigation Module (Figure 1) or Navigation Module A (Figure 5).

These equations remain largely unchanged in the CCA6 and CCA7 architectures from the prior CCA5 architecture, other than distinguishing the two Navigation Modules as mentioned in the text.

***s’_series****_t_* = [***s’****(t-3)*, ***s’****(t-2)*, ***s’****(t-1)*, ***s’****(t)*] (44)

***visual_series****_t_* = Sequential_Mod.visual_inputs( ***s’_series****_t_* ) (45)

***auditory_series****_t_* = Sequential_Mod.auditory_inputs( ***s’_series****_t_* ) (46)

***visual_motion****_t_* = Sequential_Mod.visual_match( ***visual_series****_t_* ) (47)

***auditory_motion****_t_* = Sequential_Mod.auditory_match( ***auditory_series****_t_* ) (48)

**VNM** ∈ R*^m^*^x^*^n^*^x^*^oxp^*, **AVNM** ∈ R*^m^*^x^*^n^*^x^*^oxp^* (49)

**VNM’***_t_* = **VNM***_t_* ∪ ***visual_motion****_t_* (50a)

**VNM’’***_t_* = **VNM’***_t_* ∪ ***auditory_motion****_t_* (50b)

**AVNM***_t_* = Sequential_Mod.auditory_match_process( ***auditory_series****_t_* ) ((51)

In the next section it will be seen that the Object Segmentation Gateway Module (Figure 1) will segment a sensory scene into objects of interest. The individual objects segmented in the sensory scene, as well as the entire scene itself treated as one composite object, will then trigger similar navigation maps in the Causal Memory Module to be retrieved and moved to the Navigation Module A. For example, a visual scene of a river, a rock and a leaf floating in the river, might be segmented in the river object, the rock object and the leaf object. In order to obtain the motion information about a segmented object, each segmented object navigation map must be sent to the Sequential/Error Correcting Module.

In (52) a navigation map called the Visual Segmented Navigation Map **VSNM** is defined. **VSNM***_i,t_*_-3_, **VSNM***_i,t_*_-2_, **VSNM***_i,t_*_-1_, and **VSNM***_i,t_* containing a visual segmented object on a navigation map at different time intervals, are sent from the Object Segmentation Gateway Module to the Sequential/Error Correcting Module where they are stored in vector ***visual_segmented_series****_i,t_* (53). There may be several **VNSM**’s produced for one sensory scene in the Object Segmentation Gateway Module, e.g., a sensory scene of a river with a rock and leaf floating in, it will produce a **VSNM** for the river, for the rock and for the leaf. Hence, the use of subscript *i* to refer to a particular **VSNM***_i_* for the sensory scene in a cognitive cycle.

The same pseudocode used in (47) is used again in (54) but this time on ***visual_segmented_series****_i,t_* and produces ***visseg_motion****_i,t_* which is a motion prediction vector for the motion information, if any, in ***visual_segmented_series****_i,t_*. This motion prediction vector is copied, like any spatial feature, onto the original navigation map **VSNM**_i_*_,t_* and the updated Visual Segmented Navigation Map **VSNM**’_i_*_,t_* is then sent back to the Object Segmentation Gateway Module/Navigation Module (55).

These equations remain largely unchanged in the CCA6 architecture from the prior CCA5 architecture, other than distinguishing the two Navigation Modules as mentioned in the text. This largely continues to apply to the CCA7 architecture.

**VSNM** ∈ R*^m^*^x^*^n^*^x^*^oxp^* (52)

***visual_segmented_series****_i,t_* = [**VSNM***_i,t_*_-3_, **VSNM***_i,t_*_-2_,**VSNM***_i,t_*_-1_, and **VSNM***_i,t_*] (53)

***visseg_motion****_i,t_* = Sequential_Mod.visual_match(***visual_segmented_series****_i,t_*) (54)

**VSNM’**_i_*_,t_* **= VSNM**_i_*_,t_* ∪ ***visseg_motion****_i,t_* (55)

| ***s’****(t)* | -vector representing the normalized sensory input sensory arrays of the different sensory systems **S’***_σ,t_* produced by the Input Sensory Vectors Shaping Module (Figure 5)  -sent to both Input Sensory Vectors Association Modules and to the Sequential/Error Correcting Module (Figure 1 or Figure 5 5) |
| --- | --- |
| ***s’****(t-1)* | ***s’****(t)* value in the previous cognitive cycle, i.e., t-1 |
| ***s’_series****_t_* | a time series of ***s’*** at time t, time t-1, time t-2 (two cognitive cycles ago) and time t-3  = [***s’****(t-3)*, ***s’****(t-2)*, ***s’****(t-1)*, ***s’****(t)*] |
| Sequential_Mod.visual_inputs(***s’_series****_t_*)  ***🡪 visual_series****_t_* | -pseudocode for an algorithm that extracts the visual sensory normalized inputs, i.e., **S’**_1_*_,t_* (*σ* =1 for visual system) from ***s’****(t)*  -produces ***visual_series****_t_* as its output, which essentially is [**S’**_1_*_,t_*_,_ **S’**_1_*_,t_*_-1,_ **S’**_1_*_,t_*_-2,_ **S’**_1_*_,t_*_-3_] |
| Sequential_Mod.auditory_inputs(***s’_series****_t_*)  🡪 ***auditory_series****_t_* | -pseudocode for an algorithm that extracts the auditory sensory normalized inputs, i.e., **S’**_2_*_,t_* (*σ* =2 for auditory system) from ***s’****(t)*  -produces ***auditory_series****_t_* as its output, which is [**S’**_2_*_,t_*_,_ **S’**_2_*_,t_*_-1,_ **S’**_2_*_,t_*_-2,_**S’**_2_*_,t_*_-3_] |
| Sequential_Mod.visual_match( ***visual_series****_t_* )  🡪 ***visual_motion****_t_* | -pseudocode for an algorithm that matches ***visual_series****_t_* with visual time series stored in the Sequential/Error Correcting Module (Figure 1 or Figure 5 5)  -a best match is chosen (or if no matches close enough then ***visual_series****_t_* used itself as the best match  -the best match is then updated from ***visual_series****_t_*  -the updated best match is stored in the Sequential/Error Correcting Module for future matching  -the updated best match is then transformed into a motion prediction vector (i.e., showing and predicting the motion of the object) is output as ***visual_motion****_t_* |
| Sequential_Mod.auditory_match(***auditory_series****_t_*)  🡪 ***auditory_motion****_t_* | -pseudocode for an algorithm that matches ***auditory_series****_t_* with auditory time series stored in the Sequential/Error Correcting Module (Figure 1 or Figure 5 5)  -a best match is chosen (or if no matches close enough then ***auditory_series****_t_* used itself as the best match  -the best match is then updated from ***auditory_series****_t_*  -the updated best match is stored in the Sequential/Error Correcting Module for future matching  -the updated best match is then transformed into a motion prediction vector (i.e., showing and predicting the motion of the object) and is output as ***auditory_motion****_t_* |
| **VNM** | a “Vector Navigation Map” is just another ordinary navigation map used to store the motion prediction vectors |
| **AVNM** | an “Audio Vector Navigation Map” is just another ordinary navigation map used to store more detailed motion prediction vectors about the sound patterns, useful for advanced analysis of sound patterns in perceiving the environment and for language |
| **VNM***_t_* ∪ ***visual_motion****_t_*  **🡪 VNM’***_t_* | the ***visual_motion****_t_* motion prediction vector is copied to, i.e., stored, in the Vector Navigation Map **VNM** just like any other spatial feature – binding of temporal features as a spatial feature occurs here  **VNM’**_t_ is the updated **VNM** (essentially a new navigation map has ***visual_motion****_t_* copied to it) |
| **VNM’***_t_* ∪ ***auditory_motion****_t_*  **🡪 VNM’’***_t_* | -the ***auditory_motion****_t_* motion prediction vector is copied to, i.e., stored, in the Vector Navigation Map **VNM’** just like any other spatial feature – binding of temporal features as a spatial feature occurs here again  **-VNM’’**_t_ is the updated **VNM’** (essentially **VNM’’**_t_ is a navigation map which has ***visual_motion****_t_* and ***auditory_motion****_t_* copied to it – vectors respectively showing the previous and predicted motion of an object based on visual sensory inputs and based on auditory sensory inputs )  -(note: in the simulation at present if there are different ***visual_motion****_t_* and ***auditory_motion****_t_* vectors, i.e., pointing in different directions, then the better quality result is used as a sole result in upstream algorithms in the Navigation Module; in the future more sophisticated algorithms can be used to take advantage of these motion prediction vectors) |
| Sequential_Mod.auditory_match_process (***auditory_series****_t_* )  🡪 🡪 **AVNM***_t_* | -pseudocode for an algori_t_hm that matches ***auditory_series****_t_* with auditory time series stored in the Sequential/Error Correcting Module (Figure 1 or Figure 5 5)  -a best match is chosen (or if no matches close enough then ***auditory_series****_t_* used itself as the best match  -the best match is then updated from ***auditory_series****_t_*  -the updated best match is stored in the Sequential/Error Correcting Module for future matching  -the updated best match is then transformed into a complex advanced motion prediction vector (i.e., showing in more detail the pattern of the auditory sensations) and is output as **AVNM**_t_ (which as described above is an “Audio Vector Navigation Map”) |
| **VSNM***_i,t_* | -a “Visual Segmentation Navigation Map” **VSNM** which is an ordinary navigation map with visually segmented information stored on it  -the Object Segmentation Gateway Module (Figure 1 or Figure 5 5) will “segment” a sensory scene into objects of interest (described in more detail in the following section)  -each segmented object is treated as a separate navigation map (there is also a navigation map of all the objects together on the navigation map)  -the subscript *i* refers to multiple **VSNM**’s that can be created for a given sensory scene  -the subscript *t* refers to time since the values change each cognitive cycle  -it is useful to calculate motion prediction vectors, if they exist (often they will not) for each segmented object (e.g., if there is a river object, a rock object and leaf floating in the river object, then it is very useful to segment the sensory scene into these objects (i.e., river, rock and leaf) and a motion prediction vector to show the motion of the leaf is very useful – thus there will be **VSNM**_1_*_,t_* for river, **VSNM**_2_*_,t_* for rock and **VSNM**_3_*_,t_* for leaf, in this example)  -only visual motion is calculated for segmented objects (auditory motion and motion of other senses is not calculated in the current simulation, but could be in the future, for example, the motion of a radar signal in a future embodiment) |
| ***visual_segmented_series****_i,t_* | -a time series of the current **VSNM**_i_*_,t_* and **VSNM**_i_ from one cognitive cycle ago (i.e., t-1), two cycles ago (i.e., t-2) and three cycles ago (i.e., t-3)  = [**VSNM**_i_*_,t_*_-3_, **VSNM**_i_*_,t_*_-2_,**VSNM_i_*_,t_***_-1_, and **VSNM**_i_*_,t_*]  -if there were three **VSNM**’s produced from scene (such as the example of the river with the rock and the leaf floating in it) then there would be three different ***visual_segmented_series****_i,t_* produced, with i=1, i=2, and i=3 |
| Sequential_Mod.visual_match(***visual_segmented_series****_i,t_*)  ***🡪 visseg_motion****_i,t_* | -pseudocode for an algorithm that matches ***visual_segmented_series****_i,t_* with visual time series stored in the Sequential/Error Correcting Module (Figure 1 or Figure 5 5)  -a best match is chosen (or if no matches close enough then ***visual_segmented_series****_i,t_* is used itself as the best match  -the best match is then updated from ***visual_segmented_series****_i,t_*  -the updated best match is stored in the Sequential/Error Correcting Module for future matching  -the updated best match is then transformed into a motion prediction vector (i.e., showing and predicting the motion of the object) and is output as ***visseg_motion****_i,t_* |
| **VSNM***_i,t_* ∪ ***visseg_motion****_i,t_*  **🡪 VSNM’***_i,t_* | **-VSNM**_i_*_,t_* was previously received from the Object Segmentation Gateway Module (Figure 1 or Figure 5 5) containing the spatial information about an object (also received and stored were [**VSNM***_i,t-3_*, **VSNM***_i,t_*_-2_,**VSNM***_i,t_*_-1_, and **VSNM***_i,t_*] so that a motion prediction vector could be calculated)  **-**in this step the motion prediction vector ***visseg_motion****_i,t_* about the object (e.g., perhaps the leaf in the example above) in this **VSNM**_t_ navigation map is copied to the **VSNM_i_*_,t_*** navigation map–binding of temporal features as a spatial feature occurs here for this segmented object  -**VSNM’***_i,t_* is produced in this operation (i.e., the original **VSNM***_i,t_* plus the motion prediction vector (***visseg_motion****_i,t_*) and is sent back to Object Segmentation Gateway Module/Navigation Module (Figure 1 or Figure 5 5)  -if there were three **VSNM**’s produced from scene (such as the example of the river with the rock and the leaf floating in it) then there would be three different **VSNM’***_i,t_* produced and returned to the Object Segmentation Gateway Module/Navigation Module (Figure 1 or Figure 5 5), with *i*=1, *i*=2, and *i*=3 |

Table A8. Explanation of Symbols and Pseudocode in Equations (44) – (55)

| **Input:** | - ***s’****(t)* is a vector representing all the normalized sensory input arrays **S’**_1_*_,t_* _…_ **S’**_ϴ__*_σ,t_*  (**s’**(*t*-1), **s’**(*t*-2), **s’**(*t*-3) also from *t-*1, *t-*2 and *t*-3 previous cognitive cycles are stored so that a time series can be processed)  **-VSNM**_i_*_,t_* is one of several **VSNM** navigation maps propagated from the Object Segmentation Gateway Module containing visual segments (hence the name **VSNM**), i.e., segments of the sensory scene recognized as distinct objects  -e.g., if the sensory scene was a river with a rock and a leaf floating down a river, a **VSNM**_1_*_,t_* could contain the river, a**VSNM**_2_*_,t_* could contain the rock, and a **VSNM**_3_*_,t_* could contain the leaf – they are being sent to the Sequential/Error Correcting Module for temporal binding of motion, i.e., for insertion of a motion prediction vector if the object is moving |
| --- | --- |
| **Output:** | **-VNM’**’_t_ is a navigation map binding visual motion and auditory motion of the sensory scene) propagated to the Navigation Module A complex (Figure 1 or Figure 5 5)  **-AVNM**_t_  is a navigation map binding advanced auditory patterns propagated to the Navigation Module A complex (Figure 1 or Figure 5 5) (useful for auditory analysis as in better recognition of the environment and language)  **-VSNM’**_i_*_,t_* is the original **VSNM**_i_*_,t_* from the Object Segmentation Gateway Module with a motion prediction vector added if the object was moving (or else unchanged if there was no movement), and it is then returned to the Object Segmentation Gateway Module/Navigation Module A (Figure 1 or Figure 5 5) |
| **Description:** | **-**If there is movement in a sensory scene rather than requiring thirty versions of the sensory scene per second, only a single version is required but a motion prediction vector is added to show motion that has occurred and is still predicted to occur.  -**VNM’**’*_t_* is a navigation map produced with motion prediction vectors showing any motion with regard to overall visual and sound features in the sensory scene.  **-AVNM***_t_* is a navigation map produced with multiple motion prediction vectors showing more detailed and advanced sound patterns useful for environment recognition and spoken language.  -**VSNM’***_i,t_* is a navigation map for a given object (or “segment”) of the sensory scene with a motion prediction vector added if there is motion of the object. There can be several (i.e., *i*=1, *i*=2, and so on) **VSNM’***_i,t_* navigation maps for any given scene.  -**VNM’**’*_t_* , **AVNM***_t_* , and **VSNM’**_i_*_,t_* are sent to the Object Segmentation Gateway Module/Navigation Module (Figure 1 or Figure 5 5).  -This module allows a solution to the temporal binding.  -Although the examples such as river, rock and leaf floating in it, are very concrete ones, there can be binding of motion of more abstract concepts by the same mechanisms described above.  -Applies to CCA5, CCA6 and CCA7 versions of the architecture |

Table A9. Summary of the Operation of the Sequential/Error Correcting Module per Equations (44) – (55)

A.5 The Object Segmentation Gateway Module

This section of Appendix A applies to the CCA5 (shown in Figure 1), the CCA6 (Figure 5) and the CCA7 (Figure 9) versions of the Causal Cognitive Architecture.

At this point the sensory inputs have been transformed into best matching and updated visual, auditory, and olfactory local navigation maps (represented by the vector ***lnm****_t_* ). The local navigation maps **LNM**s are propagated to the Object Segmentation Gateway Module. The complex of three tightly connected modules—the Object Segmentation Gateway Module, the Navigation Module and the Causal Memory Module will transform the input sensory data into a Working Navigation Map **WNM** upon which instinctive and learned primitives (i.e., essentially small algorithms) can act and possibly produce an action output.

The Object Segmentation Gateway Module will attempt to segment each sensory scene into different objects. In the current version of the CCA6 it is only performed visually—the module attempts to recognize coherent visual shapes in the sensory scene. (In theory, this could be done with multiple senses, and may be done in future versions of the architecture.)

Continuing the example given above of a sensory scene with only visual stimuli– a river with a rock and leaf floating in the river, the leaf, the rocks and the river are recognized by the Object Segmentation Gateway Module and segmented as separate objects each on their own **VSNM** navigation map (while at the same time keeping the entire scene and all objects on another navigation map). There will be an attempt to calculate motion prediction vectors for each different object’s navigation map, but only the leaf will have a motion prediction vector since its position is changing in this example. Note that the use of labels such as ‘river’, ‘rocks’, ‘leaf’ is for the benefit of the reader. The CCA6 does not have a full English language implemented at this time, and uses its own internal labels. As well, current recognition is from a catalog of objects and simulated—the architecture has little deep understanding of the objects.

The “Visual Segmentation Navigation Map” **VSNM***_i,t_* (52) is an ordinary navigation map with visually segmented information stored on it, i.e., each object such as the leaf floating in the river, the rocks, and the river of the example get put onto a separate **VSNM***_i_* navigation map. For example, **VSNM**_1_*_,t_* for river, **VSNM**_2_*_,t_* for one of the group of rocks, and perhaps **VSNM**_3_*_,t_* for the leaf. (There is a subscript *t* since the values of these navigation maps change each cognitive cycle.)
The pseudocode/algorithm Object_Seg_Mod.visualsegment in equation (61) takes the best-matching visual input sensory local navigation map **LNM**_(1,_ *_ϓ ,t_*_)_ and segments it into whatever objects it can find in its local memory stores or that meet certain algorithmic criteria (e.g., pixels separate from other pixels, and so on). The navigation maps **VSNM**_t,i=1..ϴ_i_ (e.g., in the example above of the river, group or rocks and leaf producing **VSNM**_1_*_,t_* for river, **VSNM**_2_*_,t_* for one of the group of rocks, and perhaps **VSNM**_3_*_,t_* for the leaf) are produced as a result. These navigation maps **VSNM**_t,i=1..ϴ_i_ are sent to Sequential/Error Correcting Module where according to equations (52–55) attempts are made to see if there is motion occurring (via time series of **VSNM**’s from different cognitive cycles) and if so a motion prediction vector is bound onto the particular **VSNM** navigation map, which is returned back to the Object Segmentation Gateway.

One of the arguments to pseudocode/algorithm Object_Seg_Mod.visualsegment (61) is as noted above the best-matching visual input sensory local navigation map **LNM**_(1,_ *_ϓ ,t_*_)_. This **LNM** is actually transmitted in parallel to the Object Segmentation Gateway Module along with the other **LNM**s from other sensory system. Having the different sensory systems’ best-matching **LNM**s via vector ***lnm***, and in (56) it is trivial to extract **LNM**_(1,_ *_ϓ ,t_*_)_. Another argument is the contextual value **CONTEXT**, which actually is a navigation map, and will help influence which objects are detected. In (57–59) it is seen to be set to the value of the previous cognitive cycle’s Working Navigation Map **WNM’**, which effectively is the previous cognitive cycle’s action taken or intermediate results. The contextual value will help influence which objects are detected. The third argument to (61) is **VNM’’**_t_. As shown above in the Sequential/Error Correcting Module, **VNM’’**_t_  shows the main visual motion and sound motion of the overall sensory scene. The vector navigation map **VNM’’**_t_ helps influence segmentation in (61) by different motions and sound production. In (62) **LNM**_(1,_ *_ϓ ,t_*_)_ is updated to **LNM’**_(1,_ *_ϓ ,t_*_)_ with the information (i.e., motion prediction vectors, which does include visual and sensory motion of the main object in the scene) from **VNM’’**_t_ .

These equations remain largely unchanged in the CCA6 architecture from the prior CCA5 architecture.

**LNM**_(1,_ *_ϓ ,t_*_)_ = ***lnm****_t_*[0] (56)

**CONTEXT** = ∈ R*^m^*^x^*^n^*^x^*^oxp^* (57)

**WNM’** = ∈ R*^m^*^x^*^n^*^x^*^oxp^* (58)

**CONTEXT***_t_* **= WNM’***_t_*_-1_ (59)

ϴ_i = total objects segmented in this sensory scene ∈ N (60)

**VSNM***_t_*_,_*_i_*_=1..ϴ_i_ = Object_Seg_Mod.visualsegment( **LNM**_(1,_ *_ϓ ,t_*_)_, **CONTEXT***_t_***_,_ VNM’’***_t_* ) (61)

**LNM’**_(1,_ *_ϓ ,t_*_)_ = **LNM**_(1,_ *_ϓ ,t_*_)_ ∪ **VNM’’***_t_* (62)

| **LNM**_(_*_σ_*_,_ *_ϓ ,t_*_)_  Equation (18) described in an earlier section | the local navigation map **LNM** stored in the Input Sensory Vectors Association Module *σ* with map number *ϓ* which best matches in the incoming *σ* sensory inputs (e.g., if *σ* = 1 then it would be visual sensory inputs)  e.g., if the visual sensory inputs best match the local navigation map #3456 in the visual module of the Input Sensory Vectors Association Modules, then at that moment **LNM**_(1,_ *_ϓ ,t_*_)_ would be navigation map #3456 in the visual module  -Applies to CCA5, CCA6 and CCA7 versions of the architecture |
| --- | --- |
| **LNM**_(_*_σ_*_,_ *_ϓ ,t_*_)_ ∪ **S’***_σ,t_*  **🡪 LNM**  Equation (21) described previously | as shown previously:  update **LNM**_(_*_σ_*_,_ *_ϓ ,t_*_)_ with any new information in input sensory signal **S’***_σ,t_* (i.e., copy **S’***_σ,t_* onto **LNM_(_***_σ_*_,_ *_ϓ ,t_*_)_ ) thereby creating an updated **LNM**_(_*_σ_*_,_ *_ϓ ,t_*_)_ |
| **LNM**_(_*_σ_*_,_*_new_map,t_*_)_∪ **S’***_σ,t_*  **🡪 LNM**  Equation (22) described previously | OR  copy **S’***_σ,t_* onto an empty **LNM**_(_*_σ_*_,_ *_new_map_* *_,t_*_)_ thereby creating an updated  **LNM**_(_*_σ_*_,_ *_ϓ ,t_*_)_ |
| ***lnm****_t_*  Equation (23) described previously | i.e., a vector holding [**LNM**_(1,_ *_ϓ ,t_*_)_, **LNM**_(2,_ *_ϓ ,t_*_)_, **LNM**_(3,_ *_ϓ ,t_*_)_, …, **LNM**_(_*_ϴ_σ_*_,_ *_ϓ ,t_*_)_] -- the local navigation maps **LNM** of each sensory system which best match the corresponding sensory input array **S’***_σ,t_* and then are updated to **LNM** with the actual sensory information conveyed by **S’***_σ,t_* |
| **LNM**_(1,_ *_ϓ ,t_*_)_ | from the definition of ***lnm****_t_* this is just the first member of ***lnm****_t_* which is ***lnm****_t_*[0] (indexing starts from 0 in this array) which is the **LNM** for the visual sensory inputs |
| **WNM’** | -Working Navigation Map  -the current Working Navigation Map **WNM’**_t_  is the navigation map which the Navigation Module A focuses its attention on, i.e., applies operations on to make a decision to take some sort or no action  -as will be seen in the following sections, a navigation map can be assigned as being the **WNM’**_t_ for that cognitive cycle, and then a different one in the next cognitive cycle, and so on, depending on the sensory information being processed and the results obtained |
| **CONTEXT** | -a normal navigation map (i.e., same dimensions as the other navigation maps in the architecture) used to hold contextual information which help influence which objects are detected  -at present it is set to the value of the previous (i.e., in the previous cognitive cycle) Working Navigation Map **WNM’**_t-1_ |
| **VNM**  Equation (49) described previously | a “Vector Navigation Map” is just another ordinary navigation map used to store the motion prediction vectors |
| **VNM’’**_t_  Equations (50a, 50b)  described previously | as described previously in the Sequential/Error Correcting Module:  -essentially **VNM’’**_t_ is a navigation map which has ***visual_motion****_t_* and ***auditory_motion****_t_* copied to it – vectors respectively showing the previous and predicted motion of an object based on visual sensory inputs and based on auditory sensory inputs  -tends to refer to motion of the overall scene or major object rather than the segmented objects in the sensory scene |
| **VSNM**_i_*_,t_* **🡪 VSNM’**_i_*_,t_*  (Note: **VSNM**_i_*_,t_* is first created in the Object Segmentation Gateway Module, and then updated to **VSNM’**_i_*_,t_* in the Sequential/Error Correcting Module) | -a “Visual Segmentation Navigation Map” **VSNM** which is an ordinary navigation map with visually segmented information stored on it  -the Object Segmentation Gateway Module will “segment” a sensory scene into objects of interest  -each segmented object is treated as a separate navigation map (there is also a navigation map of all the objects together on the navigation map)  -the subscript *i* refers to multiple **VSNM**’s that can be created for a given sensory scene  -the subscript *t* refers to time since the values change each cognitive cycle  -it is useful to calculate motion prediction vectors, if they exist (often they will not) for each segmented object (e.g., if there is a river object, a rock object and leaf floating in the river object, then it is very useful to segment the sensory scene into these objects (i.e., river, rock and leaf) and a motion prediction vector to show the motion of the leaf is very useful – thus there will be **VSNM**_1_*_,t_* for river, **VSNM**_2_*_,t_* for rock and **VSNM**_3_*_,t_* for leaf, in this example)  -continuing this example: **VSNM**_1_*_,t_* for river, **VSNM**_2_*_,t_* for rock and **VSNM**_3_*_,t_* for leaf will be propagated to the Sequential/Error Correcting Module for computation of motion prediction vectors for each of these **VSNM**’s  -continuing this example: a motion prediction vector is computed and stored on **VSNM**_3_*_,t_* for leaf, but **VSNM**_1_*_,t_* for river, **VSNM**_2_*_,t_* for rock did not have enough motion for such vectors and are unchanged  -continuing this example: **VSNM’**_1_*_,t_* for river (unchanged), **VSNM’**_2_*_,t_* for rock (unchanged) and **VSNM’**_3_*_,t_* for leaf (motion prediction vector added) are then returned back to the Object Segmentation Gateway Module |
| **VSNM**_t,i=1..ϴ_i_ | -all the Visual Segmentation Navigation Maps **VSNM**’s created for a sensory scene  -for example, in the example just given there would be **VSNM**_1_*_,t_* for river, **VSNM**_2_*_,t_* for rock and **VSNM**_3_*_,t_* for leaf |
| Object_Seg_Mod.visualsegment( **LNM**_(1,_ *_ϓ ,t_*_)_, **CONTEXT**_t_**_,_ VNM’’**_t_ )  🡪 **VSNM**_t,i=1..ϴ_i_ | -pseudocode for an algorithm that takes the best-matching visual input sensory local navigation map **LNM**_(1,_ *_ϓ ,t_*_)_ and segments it into whatever objects it can find in its local memory stores or that meet certain algorithmic criteria (e.g., pixels separate from other pixels, and so on)  -currently only segments via visual features (although one of the arguments **VNM’’**_t_ can contain information about auditory motion)  -arguments were described above:  **LNM**_(1,_ *_ϓ ,t_*_)_ : the best-matching visual input sensory local navigation map  **CONTEXT**_t_ : to hold contextual information which help influence which objects are detected  **VNM’’**_t_ : auditory and visual motion of the overall scene or major object  -produces **VSNM**_t,i=1..ϴ_i_ which are all the Visual Segmentation Navigation Maps **VSNM**’s created for a sensory scene |
| **LNM’**_(1,_ *_ϓ ,t_*_)_ | **LNM’**_(1,_ *_ϓ ,t_*_)_  is the updated best-matching visual sensory Local Navigation Map **LNM**_(1,_ *_ϓ ,t_*_)_ further updated with any visual or auditory motion prediction vectors from **VNM’’**_t_ |

Table A10. Explanation of Symbols and Pseudocode in Equations (56) – (62)

| **Initial Input:** | **-AVNM**_t :_ a navigation map binding advanced auditory patterns; used in the next section  **-LNM**_(1,_ *_ϓ ,t_*_)_ : the best-matching visual input sensory local navigation map; from the Input Sensory Vectors Association Module will be segmented for objects in the sensory scene  -other sensory system **LNM**s (represented by ***lnm****_t_*); used in the next section  -**WNM’**_t_ : Working Navigation Map from the Navigation Module; stored and used as **WNM’**_t-1_ to produce a value for **CONTEXT**_t_ which holds contextual information which helps influence which objects are detected  -**VNM’’**_t_ : auditory and visual motion of the overall scene or major object; from the Sequential/Error Correcting Module |
| --- | --- |
| **Initial Output:** | -produces **VSNM**_t,i=1..ϴ_i_ which are all the Visual Segmentation Navigation Maps **VSNM**’s created for a sensory scene; sent to the Sequential/Error Correcting Module |
| **Final Input:** | **-VSNM’**_t,i=1..ϴ_i_ : the Sequential/Error Correcting Module will add where indicated a motion prediction vector to the **VSNM**’s it receives if there is motion of the object, and then send the **VSNM’**_t,i i=1..ϴ_i_ (i.e., all the **VSNM**’s) back to the Object Segmentation Gateway Module  -e.g., in the example above: : **VSNM’**_1_*_,t_* for river (unchanged), **VSNM’**_2_*_,t_* for rock (unchanged) and **VSNM’**_3_*_,t_* for leaf (motion prediction vector added since apparent motion of leaf detected) |
| **Final Output:** | **- VSNM’**_t,i=1..ϴ_i_ : visual segmented (i.e., objects separated) navigation maps with motion prediction vectors if motion detected  - **AVNM**_t :_ a navigation map binding advanced auditory patterns; used in the next section  - **LNM’**_(1,_ *_ϓ ,t_*_)_ : updated best-matching visual sensory Local Navigation Map **LNM**_(1,_ *_ϓ ,t_*_)_ including any motion prediction vectors from **VNM’’**_t_  - other sensory system **LNM**s (represented by ***lnm****_t_*); passed through the module; used in the next section |
| **Description:** | -This module attempts to segment the input sensory by objects in it.  -Segmentation is largely visual at present (although some limited auditory motion at present).  -After segmenting a sensory scene into objects, the individual objects are sent to the Sequential/Error Correcting Module to detect motion of the objects, and if motion exists then a motion prediction vector is added to the **VSNM** navigation maps representing each object producing **VSNM’**.  -Applies to CCA5, CCA6 and CCA7 versions of the architecture |

Table A11. Summary of the Operation of the Object Segmentation Gateway Module per Equations (56) – (62)

**A.6 The Causal Memory Module**

This section of Appendix A applies to the CCA5 (shown in Figure 1), the CCA6 (Figure 5) and the CCA7 (Figure 9) versions of the Causal Cognitive Architecture.

The input sensory inputs at this point have been transformed as follows:

- visual sensory inputs 🡪 VSNM’_(t,i=1...ϴ_i)_ : visual segmented (i.e., objects separated) navigation maps with motion prediction vectors if motion detected
- visual sensory inputs 🡪 LNM’_(1,_ *_ϓ ,t_*_)_ : updated best-matching visual sensory Local Navigation Map LNM_(1,_ *_ϓ ,t_*_)_ including any motion prediction vectors from VNM’’_t_
- auditory sensory inputs 🡪 **AVNM**_t :_ a navigation map binding auditory patterns
- olfactory sensory inputs 🡪 **LNM**_(3,_ *_ϓ ,t_*_)_ : updated best-matching olfactory sensory Local Navigation Map **LNM**_(3,_ *_ϓ ,t_*_)_
- other sensory systems inputs 🡪 **LNM**_(4…_*_n_σ_*_,_ *_ϓ ,t_*_)_ **:** updated best-matching other sensory (e.g., tactile, radar, etc.) Local Navigation Maps **LNM**_(4…_*_n_σ_*_,ϓ_*_,t_*_)_

These single sensory system navigation maps will now be matched against the previously stored multi-sensory navigation maps stored in the Causal Memory Module. The matching algorithm Object_Seg_Mod.match_best_map (63) chooses previously stored Navigation Map A as the best match. Thus, Navigation Map A (i.e., ***χ*** = [[modcode=Causal_Mem_Mod, *mapno*=3579], x,y,z] ) is set to become the Working Navigation Map **WNM** (63).



**WNM** must be updated with the actual sensory inputs, since after all it is at this point simply a retrieved best matching navigation map. If there are too many changes between the actual sensory inputs ***actual****_t_* (64) and an arbitrary threshold h’ (65), then rather than update **WNM** a new Working Navigation Map **WNM’** will be created from the actual sensory inputs ***actual****_t_* via copying the actual sensory inputs ***actual****_t_* to an empty navigation map **TempMap** (69). Note from (66) that **TempMap** is actually defined as the temporary memory area “TempMap” within the Navigation Module A(Figure 5). Often values from the Causal Memory Module (e.g., a linked navigation map) or Navigation Module may have automatically been sent (i.e., copied) to **TempMap**. Thus, the pseudocode Nav_ModA.TempMap.erase() (68) is called to ensure **TempMap** has no contents and is equivalent to an empty navigation map before using it in (69).

However, if **WNM** (i.e., which is a retrieved navigation map from the Causal Memory Module) (63) is close enough to the actual input sensory inputs ***actual****_t_ ,* then the information from ***actual****_t_* is copied to **WNM**, and creates the updated Working Navigation Map **WNM’***_t_* (67).

In (70) **WNM’** is stored in the Causal Memory Module—in future operations of the architecture it will also be matched against various sensory inputs. This pseudocode also specifies that there is updating of the ***linkaddresses*** to and from other navigation maps in the Causal Memory Module and the updated Working Navigation Map **WNM’***_t_* .

These equations remain largely unchanged in the CCA6 architecture from the prior CCA5 architecture, other than distinguishing the two Navigation Modules A and B. This still largely applies to the CCA7 architecture.

**WNM***_t_* = Object_Seg_Mod**.**match_best_map
**(VSNM’**_(_*_t,i_*_=1…ϴ_i)_ , **LNM’**_(1,_ *_ϓ ,t_*_)_ , **AVNM***_t_* , **LNM**_(3,_ *_ϓ ,t_*_)_ , **LNM**_(4…_*_n_σ_*_,ϓ_*_,t_*_)_**)** (63)

***actual****_t_* = [**VSNM’**_(_*_t_*_,_*_i_*_=1...ϴ_i )_, **LNM’**_(1,_ *_ϓ ,t_*_)_ , **AVNM**_t_ , **LNM**_(3,_ *_ϓ ,t_*_)_ , **LNM**_(4…_*_n_σ_*_,ϓ_*_,t_*_)_] (64)

h’ **=** number of differences allowed to be copied onto existing navigation map ∈ R (65)

‘

**TempMap** = Nav_ModA.TempMap ∈ R*^m^*^x^*^n^*^x^*^oxp^* (66)

| Object_Seg_Mod.differences(***actual****_t_* , **WNM***_t_*)**| ≤**h’**,**

⇒ **WNM’***_t_* **= WNM***_t_* ∪ ***actual****_t_* (67)

Nav_ModA.TempMap.erase() (68)

| Object_Seg_Mod.differences(***actual****_t_* , **WNM***_t_*)**| >**h’**,**

⇒ **WNM’***_t_* **= TempMap** ∪ ***actual****_t_* (69)

Causal_Mem_Mod.store_WNM_update_links(**WNM’***_t_*) (70)

| Object_Seg_Mod.match_best_  map**(**  **VSNM’**_(_*_t,i_*_=1…ϴ_i)_ ,  **LNM’**_(1,_ *_ϓ ,t_*_)_ ,  **AVNM**_t_ ,  **LNM**_(3,_ *_ϓ ,t_*_)_ ,  **LNM**_(4…_*_n_σ_*_,ϓ_*_,t_*_)_ **)**  **🡪 WNM** | -pseudocode for an algorithm that takes processed single sensory navigation maps and matches them to the best matching multisensory navigation map in the Causal Memory Module  -note that this pseudocode is run from the Object Segmentation Gateway Module since all of the arguments of this pseudocode are in the latter module  -a small example of this algorithm is illustrated in Figure 5 where the best-matching visual local navigation map **LNM’**_(1,_ *_ϓ ,t_*_)_, the visual segmentation navigation maps **VSNM’**_(_*_t,i_*_=1…ϴ_i),_ the navigation map binding auditory patterns **AVNM**_t ,_ and the olfactory local navigation map **LNM**_(3,_ *_ϓ ,t_*_)_ , are matched against the multi-sensory navigation maps of the Causal Memory Module  **-**the arguments to the algorithm (**VSNM’**_(_*_t,i_*_=1...ϴ_i )_, **LNM’**_(1,_ *_ϓ ,t_*_)_ , **AVNM**_t_ , **LNM**_(3,_ *_ϓ ,t_*_)_ , **LNM**_(4…_*_n_σ_*_,ϓ_*_,t_*_)_) are described individually below  -the best matching multisensory map is designated as the Working Navigation Map **WNM** |
| --- | --- |
| argument used in match_best_  map**: VSNM’***_t,i_*_=1...ϴ_i_  **VSNM***_t,i_*_=1…ϴ_i_ **🡪 VSNM’***_t,i_*_=1...ϴ_i_  (Note: **VSNM** is first created in the Object Segmentation Gateway Module, and then updated to **VSNM’** in the Sequential/Error Correcting Module) | -a “Visual Segmentation Navigation Map” **VSNM** is an ordinary navigation map with visually segmented information stored on it  -the Object Segmentation Gateway Module will “segment” a sensory scene into objects of interest  -each segmented object is treated as a separate navigation map (there is also a navigation map of all the objects together on the navigation map)  -the subscript *i* refers to multiple **VSNM**’s that can be created for a given sensory scene  -*i*=1…ϴ_i refers to the full set for **VSNM**’s created for a given sensory scene, i.e., **VSNM**_1_ to the last **VSNM**_ϴ_i_ created  -the subscript *t* refers to time since the values change each cognitive cycle  -it is useful to calculate motion prediction vectors, if they exist (often they will not) for each segmented object (e.g., if there is a river object, a rock object and leaf floating in the river object, then it is very useful to segment the sensory scene into these objects (i.e., river, rock and leaf) and a motion prediction vector to show the motion of the leaf is very useful – thus there will be **VSNM**_1_*_,t_* for river, **VSNM**_2_*_,t_* for rock and **VSNM**_3_*_,t_* for leaf, in this example)  -continuing this example: **VSNM**_1_*_,t_* for river, **VSNM**_2_*_,t_* for rock and **VSNM**_3_*_,t_* for leaf will be propagated to the Sequential/Error Correcting Module for computation of motion prediction vectors for each of these **VSNM**’s  -continuing this example: a motion prediction vector is computed and stored on **VSNM**_3_*_,t_* for leaf, but **VSNM**_1_*_,t_* for river, **VSNM**_2_*_,t_* for rock did not have enough motion for such vectors and are unchanged  -continuing this example: **VSNM’**_1_*_,t_* for river (unchanged), **VSNM’**_2_*_,t_* for rock (unchanged) and **VSNM’**_3_*_,t_* for leaf (motion prediction vector added) are then returned back to the Object Segmentation Gateway Module |
| argument used in match_best_  map**:**  **LNM’**_(1,_ *_ϓ ,t_*_)_ | **-LNM’**_(1,_ *_ϓ ,t_*_)_  is the updated best-matching visual sensory Local Navigation Map --**LNM**_(1,_ *_ϓ ,t_*_)_ is further updated with any visual or auditory motion prediction vectors from **VNM’’**_t_  to create **LNM’**_(1,_ *_ϓ ,t_*_)_ |
| argument used in match_best_  map**:**  **AVNM***_t_*  Sequential_Mod.auditory_  match_process (***auditory_series****_t_* )  🡪 🡪 **AVNM***_t_* | -pseudocode for an algorithm that matches ***auditory_series****_t_* with auditory time series stored in the Sequential/Error Correcting Module  -a best match is chosen (or if no matches close enough then ***auditory_series****_t_* used itself as the best match)  -the best match is then updated from ***auditory_series****_t_*  -the updated best match is stored in the Sequential/Error Correcting Module for future matching  -the updated best match is then transformed into a complex advanced motion prediction vector (i.e., showing in more detail the pattern of the auditory sensations) and is output as **AVNM**_t_ (which as described above is an “Audio Vector Navigation Map”) |
| argument used in match_best_  map**:**  **LNM**_(3,_ *_ϓ ,t_*_)_ | the local navigation map **LNM** stored in the Input Sensory Vectors Association Module *σ* with map number *ϓ* which best matches in the incoming *σ* sensory inputs (e.g., if *σ* = 3 then it would be olfactory sensory inputs) |
| argument used in match_best_  map**:**  **LNM**_(4…_*_n_σ_*_,ϓ_*_,t_*_)_ | -for illustrative purposes only three sensory systems: visual, auditory and olfactory are often used here, but there can be additional ones such as tactile, or synthetic ones such as radar, etc.  -4…*n_σ* means from **LNM**_4_ to the last sensory system being used and its local navigation map **LNM***_n_σ_* |
| ***actual****_t_* | -this is a vector that holds all the arguments for the pseudocode match_best_map  =[**VSNM’**_(t,i=1...ϴ_i )_, **LNM’**_(1,_ *_ϓ ,t_*_)_ , **AVNM**_t_ , **LNM**_(3,_ *_ϓ ,t_*_)_ , **LNM**_(4…_*_n_σ_*_,ϓ_*_,t_*_)_] |
| **TempMap** (NavMod A module) | -a navigation map of the same dimensions as the other navigation maps in the architecture but it is a short-term memory region in the Navigation Module A Nav_ModA.TempMap rather than being an ordinary navigation map |
| Object_Seg_Mod.  differences() | -pseudocode for an algorithm that calculates the differences between two navigation maps  -similar to Input_Assocn_Mod*_σ_*.differences described above (21, 22)  -used in (67) and (68) to calculate the differences between the processed input sensory signal ***actual****_t_* and the retrieved best matching multi-sensory navigation map **WNM***_t_* |
| **WNM***_t_* ∪ ***actual****_t_*  **🡪 WNM’***_t_* | -**WNM** must be updated with the actual sensory inputs  -the processed sensory inputs ***actual****_t_* are copied to **WNM**, thereby forming **WNM’** the updated Working Navigation Map which will be used by the Navigation Module in the section below |
| h’  **TempMap***_t_* ∪ ***actual****_t_*  **🡪 WNM’***_t_* | -**WNM** must be updated with the actual sensory inputs  -if there are too many changes between the actual sensory inputs ***actual****_t_* (64) and this arbitrary threshold h’, then rather than update **WNM** , the actual sensory inputs ***actual****_t_* will be used to create a new navigation map which is considered the Working Navigation Map **WNM’** which will be used by the Navigation Module in the section below  -as noted above **TempMap** is a temporary memory region in the Navigation Module A that simulates a navigation map; ***actual****_t_* is being copied to an empty navigation map and results in **WNM’***_t_* |
| **WNM’***_t_* | -updated Working Navigation Map  -the current Working Navigation Map **WNM’***_t_*  is the navigation map which the Navigation Module A focuses its attention on, i.e., applies operations on to make a decision to take some sort or no action  -as will be seen in the following sections, a navigation map can be assigned as being the **WNM’***_t_* for that cognitive cycle, and then a different one in the next cognitive cycle, and so on, depending on the sensory information being processed and the results obtained |
| Causal_Mem_Mod.  store_WNM_update_links (**WNM’***_t_*) | -pseudocode specifying that the updated Working Navigation Map **WNM’***_t_* is stored in the Causal Memory Module  - pseudocode specifying updating of the ***linkaddresses*** to and from the updated Working Navigation Map **WNM’***_t_* is stored in the Causal Memory Module  -in future cognitive cycles the processed sensory inputs ***actual****_t_* will be matched against the navigation maps in the Causal Memory Module which includes this newly stored navigation map |
| Nav_ModA.TempMap.erase() | -pseudocode specifying that **TempMap** is erased  -thus, it will act as an empty navigation map the next time it is required |

Table A12. Explanation of Symbols and Pseudocode in Equations (63) – (70)

| **Input:** | **-VSNM’**_(t,i=1…ϴ_i)_ : visual segmented (i.e., objects separated) navigation maps with motion prediction vectors if motion detected  **-LNM’**_(1,_ *_ϓ ,t_*_)_ : best-matching visual sensory Local Navigation Map **LNM**_(1,_ *_ϓ ,t_*_)_ including any motion prediction vectors from **VNM’’**_t_  -**AVNM**_t_ **_:_** a navigation map binding auditory patterns  -**LNM**_(3,_ *_ϓ ,t_*_)_ : the olfactory local navigation map  **-LNM**_(4…_*_n_σ_*_,ϓ_*_,t_*_)_ : other sensory local navigation maps  (these processed sensory inputs are represented by the vector ***actual****_t_* ) |
| --- | --- |
| **Output:** | **WNM’**_t_ : updated Working Navigation Map which will be used in the Navigation Module A (as well as other modules of the architecture) |
| **Description:** | -In this module the processed single sensory system navigation maps (***actual****_t_*) will be matched against the previously stored multi-sensory navigation maps stored in the Causal Memory Module.  -The best matching navigation map is then updated with the actual sensory inputs, i.e., ***actual****_t_* , and acts as the Working Navigation Map **WNM’** for this cognitive cycle.  -The current Working Navigation Map **WNM’**_t_  is the navigation map which the Navigation Module A focuses its attention on, i.e., applies operations on to make a decision to take some sort or no action. -Applies to CCA5, CCA6 and CCA7 versions of the architecture |

Table A13. Summary of the Operation of the Causal Memory Module per Equations (63) – (70)

A.7 Navigation Module A

This section of Appendix A applies to the CCA6 (Figure 5) and the CCA7 (Figure 9) versions of the Causal Cognitive Architecture.

As seen in Figure 5 there are now in the CCA6 two Navigation Modules—Navigation Module A and Navigation Module B (as opposed to the single Navigation Module in the prior CCA5 architecture, as seen in Figure 1). As seen in Figure 9 there are now in the CCA7 multiple Navigation Module B’s. However, this section still applies to the single Navigation Module A in the CCA7 architecture.

In the Navigation Module A an instinctive primitive or a learned primitive (which themselves are navigation maps) in conjunction with the Working Navigation Map **WNM’** may result in an action signal. This signal goes to the Output Vector Association Module A and to the external embodiment, completing the cognitive cycle.

In some cognitive cycles there will be no output but intermediate results from the Navigation Module A are fed back and re-operated on in the next cognitive cycle. This will be discussed in the following section. In this section, the more straightforward case is considered where a learned or instinctive primitive is applied against a Working Navigation Map **WNM’** and an action is produced.

From Figure 5 the Autonomic Module and the Goal/Emotion Module can be seen. These modules, which operate in the areas much as their names indicate, are essential for an agent such as the Causal Cognitive Architecture 6. However, these modules will not be formalized so as to focus on the Navigation Module A in this section. The *autonomic* variable (71) which reflects the energy levels and maintenance issues with embodiments of the architecture, will affect the value of the *emotion* variable (72) and the **GOAL** navigation map (73) as shown in equation (74). The **GOAL** variable reflects both intuitive and learned goals of the architecture acting as an agent and is represented as a full navigation map. The *emotion* variable will affect which features the architecture pays more attention to in their processing as well as in selecting the appropriate instinctive or learned primitive to act on the current Working Navigation Map **WNM’**.

As mentioned earlier, instinctive and learned primitives, which act as small rules or productions, are stored in modified navigation maps, called respectively instinctive primitive navigation maps **IPM**, and learned primitive navigation maps **LPM**. These primitive navigation maps have cells that contain procedures. However, the cells can also contain features and linkaddresses.

In the Navigation Module A, a learned or instinctive primitive navigation map is compared against the Working Navigation Map **WNM’**. By simple array operations and logical operations, the Navigation Module A may produce an *action* signal, i.e., a signal to move some actuator or send an electronic signal. The *action* signal goes to the Output Vector Association Module A and then to the external embodiment.

Instinctive primitives **IPM’**s are stored in the Instinctive Primitives Module and come preprogrammed with the architecture. Learned primitives **LPM**’s in the CCA6 are now distributed among the many Input Sensory Vectors Association Modules and the Causal Memory Module (Figure 5). Both instinctive and learned primitive navigation maps have cells that contain procedures. However, the cells can also contain features and linkaddresses.

The pseudocode indicating the algorithm Instinctive_Primitives_Mod**.**match_best_primitive (***actual****_t_*, *emotion_t_*, **GOAL***_t_* ) (76) will choose the most appropriate instinctive primitive **WIP***_t_* (75) to apply against the Working Navigation Map **WNM’** in the Navigation Module A. Similarly, the pseudocode indicating the algorithm Learned_Primitives_Mod**.**match_best_primitive (***actual****_t_*, *emotion_t_*, **GOAL***_t_* ) (78) will choose the most appropriate learned primitive **WLP**_t_ (77) to apply against the Working Navigation Map **WNM’** in the Navigation Module A.

Then a decision is made to use either the best instinctive primitive **WIP***_t_* or the best learned primitive **WLP**_t_ as the Working Primitive **WPR**_t_ (79) which will actually be the primitive applied against the Working Navigation Map **WNM’**. At present a simple scheme is used to make this decision. If there is no learned primitive **WLP**_t_ then the best instinctive primitive **WIP***_t_* is assigned to be the Working Primitive **WPR**_t_ (80). Otherwise, if a best learned primitive exists, then it is assigned to be the Working Primitive **WPR**_t_ (81).

At this point in the Navigation Module A there is a Working Primitive **WPR**_t_ and a Working Navigation Map **WNM’**. By simple array operations and logical operations, the Navigation Module A compares and operates on the Working Primitive **WPR**_t_ and the Working Navigation Map **WNM’**. Equation (82) shows this is indicated by pseudocode Nav_ModA.apply_primitive(**WPR***_t_*, **WNM’***_t_*). As a result, an *action_t_* value may be produced, which is a signal to move some actuator or send an electronic signal. The *action* signal goes to the Output Vector Association Module A and then to the external embodiment (Figure 5).

The *action* signal from the Navigation Module A may be, for example, **<move right>**. This signal is processed by the Output Vector Association Module A (Figure 5). The pseudocode Output_Vector_Mod**.**action _to_output( *action_t_*, **WNM’***_t_* ) (84) results in ***output_vector****_t_* which contains more detailed instructions for the actuators to cause the embodiment to move right. However, ***output_vector****_t_* is first sent to the Sequential/Error Correcting Module where it will be corrected for motion and timing issues, resulting in a ***motion_correction****_t_* vector (86). The pseudocode Output_Vector_Mod**.**apply_motion_correction( ***output_vector****_t_*, ***motion_correction****_t_* ) (87) produces the final signal ***output_vector’****_t_* which is sent to the Output Vector Shaping Module (Figure 5). In the Output Vector Shaping Module the final signal shaping and low-level transformations of the signal are performed (not indicated in the equations) to directly signal movement of the embodiment’s actuators or signal transmission of an electronic signal.

These equations in the CCA6 architecture are somewhat changed from the prior CCA5 architecture to reflect the duplication of the Navigation Modules. They continue to still apply to the CCA7 architecture with regard to Navigation Module A and related modules.

*autonomic* ∈ R (71)

*emotion* ∈ R (72)

**GOAL** ∈ R*^m^*^x^*^n^*^x^*^oxp^* (73)

[*emotion_t_*, **GOAL***_t_*] = Goal/Emotion_Mod**.**set_emotion_goal( *autonomic_t_*, **WNM’***_t-1_* ) (74)

**WIP** ∈ R*^m^*^x^*^n^*^x^*^oxp^* (75)

**WIP***_t_* = Instinctive_Primitives_Mod**.**match_best_primitive( ***actual****_t_*, *emotion_t_*, **GOAL***_t_* ) (76)

**WLP** ∈ R*^m^*^x^*^n^*^x^*^oxp^* (77)

**WLP***_t_* = Learned_Primitives_Mod**.**match_best_primitive( ***actual****_t_*, *emotion_t_*, **GOAL***_t_* ) (78)

**WPR** ∈ R*^m^*^x^*^n^*^x^*^oxp^* (79)

**WLP***_t_* **=** [ ], ⇒ **WPR***_t_* **= WIP***_t_* (80)

**WLP***_t_* **≠** [ ], ⇒ **WPR***_t_* **= WLP***_t_* (81)

*action_t_* = Nav_ModA.apply_primitive(**WPR***_t_*, **WNM’***_t_*) (82)

***output_vector***  ∈ R^n’^ (83)

*action_t_*  **=** [“move*”], ⇒ ***output_vector****_t_* = Output_Vector_Mod**.**

action_to_output( *action_t_*, **WNM’***_t_* ) (84)

***motion_correction*** ∈ R^2^ (85)

*action_t_* **=** [“move*”]**,** ⇒ ***motion_correction****_t_* = Sequential_Mod.

motion_correction( *action_t_*, **WNM’***_t_*, ***visual_series****_t_* ) (86)

***output_vector’****_t_* = Output_Vector_Mod**.**

apply_motion_correction( ***output_vector****_t_*, ***motion_correction****_t_* ) (87)

| *autonomic* | variable which reflects the energy levels and maintenance issues with embodiments of the architecture |
| --- | --- |
| *emotion* | variable which affects which features the architecture pays more attention to |
| **GOAL** | reflects both intuitive and learned goals of the architecture acting as an agent, and is represented as a full navigation map |
| Goal/Emotion_Mod.set_emotion_goal( *autonomic_t_*, **WNM’***_t_* _-1_)  🡪 [*emotion_t_*, **GOAL***_t_*] | -pseudocode that calculates the values of the *emotion* and **GOAL** variables  -Goal/Emotion_Mod refers to the Goal/Emotion Module  -the argument **WNM’***_t_* _-1_ is the Working Navigation Map from the previous (i.e., t-1) cognitive cycle |
| Instinctive_Primitives_Mod**.**  match_best_primitive  ( ***actual****_t_*, *emotion_t_*, **GOAL***_t_* )  🡪 **WIP***_t_* | -pseudocode that returns the most appropriate instinctive primitive navigation map **IPM** from the Instinctive Primitives Module given the arguments of the processed sensory inputs ***actual****_t_* , the current *emotion_t_* value, and the current **GOAL***_t_* navigation map  -Instinctive_Primitives_Mod refers to the Instinctive Primitives Module  -the most appropriate instinctive primitive **IPM** returned is considered to be the Working Instinctive Primitive **WIP***_t_* |
| Learned_Primitives_Mod**.**match_best_primitive  ( ***actual****_t_*, *emotion_t_*, **GOAL***_t_* )  🡪 **WLP***_t_* | -pseudocode that returns the most appropriate learned primitive navigation map **LPM** from the Instinctive Primitives Module given the arguments of the processed sensory inputs ***actual****_t_* , the current *emotion_t_* value, and the current **GOAL***_t_* navigation map  -Learned_Primitives_Mod refers to the Learned Primitives Module  -the most appropriate instinctive primitive **LPM** returned is considered to be the Working Learned Primitive **WLP***_t_* |
| **WLP***_t_* **=** [ ], ⇒ **WPR***_t_* **= WIP***_t_*  **WLP***_t_* **≠** [ ], ⇒ **WPR***_t_* **= WLP***_t_*  🡪 **WPR** | -the Working Primitive **WPR** is the primitive that will be applied against the Working Navigation Map **WNM’** in the Navigation Module A  -it is composed of inputs from the Working Instinctive Primitive **WIP** and the Working Learned Primitive **WLP**  -currently a simple scheme to calculate it is used since there is only a very small number of learned primitives **LPM**’s—if a Working Learned Primitive **WLP** exists then **WLP***_t_*  will be used as **WPR***_t_*, otherwise the Working Instinctive Primitive **WIP***_t_* will be used as **WPR***_t_* |
| *procedure* | -a cell in a navigation map can hold values which are *procedure* variables (35), *feature* variables (34), and linkaddress *χ’* variables (36)  -a *procedure* value could be, for example, <move right> which would indicate that the embodiment of the architecture should move towards the right |
| Nav_ModA.apply_primitive(**WPR***_t_*, **WNM’***_t_*)  🡪 *action_t_* | -Nav_ModA refers to the Navigation Module A (Figure 5)  -apply_primitive(**WPR***_t_*, **WNM’***_t_*) is pseudocode for an algorithm which applies the Working Primitive **WPR**_t_ to the Working Navigation Map **WNM’**, often producing an *action_t_* value  -note that in some cognitive cycles no *action_t_* value may result from the current **WPR***_t_* and **WNM’***_t_*  -in other cognitive cycles instead of an actionable *action_t_* value there is the feeding back of intermediate results for re-processing in the next cognitive cycle (discussed in the next section)  -the mechanism of producing an *action_t_* value is via simple array operations (both **WPR***_t_* and **WNM’***_t_* are arrays) and very simple logical operations that could be biologically feasible as possible |
| Output_Vector_Mod**.**  action_to_output( *action_t_*, **WNM’***_t_* )  🡪 ***output_vector****_t_* | -Output_Vector_Mod refers to the Output Vector Association Module A  -pseudocode describing taking the *action_t_* value and the current Working Navigation Map **WNM’***_t_*  and creating ***output_vector****_t_* which would provide more detailed instructions for the actuators of the embodiment in order to carry out the *action_t_* value  -for example, the ***action*** value could be, for example, **<move right>** which this pseudocode would transform into ***output_vector****_t_* which provides more detailed instructions for the embodiment’s actuators with regard to moving to the right |
| Sequential_Mod.  motion_correction( *action_t_*, **WNM’***_t_*, ***visual_series****_t_* )  🡪 ***motion_correction****_t_* | -Sequential_Mod refers to the Sequential/Error Correcting Module  -pseudocode which produces a motion correction value based on the relative movement of the embodiment itself and the motion which is desired to achieve  -the pseudocode considers what *action_t_* is desired, the current Working Navigation Map **WNM’***_t_*  and the current movement of the embodiment via the visual movement detected via ***visual_series****_t_* (44, 45)  -in the future additional sensory systems (e.g., a positioning system, an inertial system, and so on) could be used in addition to the information in the ***visual_series****_t_*  -the vector ***motion_correction****_t_* is computed and returned to the Output Vectors Association Module  -for example, the current ***action*** value is **<move right>** so the pseudocode would compute if any motion correction was required based on the current actual movement of the embodiment of the architecture |
| Output_Vector_Mod**.**  apply_motion_correction( ***output_vector****_t_*, ***motion_correction****_t_* )  🡪 ***output_vector’****_t_* | -Output_Vector_Mod refers to the Output Vector Association Module A  -pseudocode that applies the computed vector ***motion_correction****_t_* against the previously computed ***output_vector****_t_*  -an updated ***output_vector’****_t_*  is produced  🡪 ***output_vector’****_t_*  provides more detailed and motion corrected instructions for the actuators in this case, e.g, **<move right>**, to move the embodiment to the right |

Table A14. Explanation of Symbols and Pseudocode in Equations (71) – (87)

| **Input:** | **WPR***_t_* : the Working Primitive **WPR** is the primitive that will be applied against the Working Navigation Map **WNM’** in the Navigation Module A  **WNM’***_t_* : the current Working Navigation Map which is derived from the processed sensory inputs |
| --- | --- |
| **Output:** | *action_t_* : a signal to move some actuator or send an electronic signal  e.g., **<move right>**  it is sent to the Output Vector Association Module A where more detailed instructions are created to effect the required actuator outputs |
| **Description:** | -The Navigation Module A applies the Working Primitive **WPR***_t_* against the current Working Navigation Map **WNM’** and may produce an *action_t_*  signal (sometimes no signal is produced or sometimes there is a signal to feedback intermediate results, discussed in the next section).  -Applies to the CCA5 version of the architecture (single Navigation Module) and to the CCA6 and CCA7 versions of the architecture with respect to Navigation Module A and associated modules |

Table A15. Summary of the Operation of the Navigation Module A per Equations (71) – (87)

A.8 Feedback Signals and Intermediate Results

This section of Appendix A applies to the CCA5 version of the architecture (Figure 1) but there is only a single Navigation Module. This section of the Appendix A applies CCA6 (Figure 5) and the CCA7 (Figure 9) versions of the Causal Cognitive Architecture where there is a separate Navigation Module A and B (with the latter plural in the CCA7).

The Causal Cognitive Architecture, including the CCA6, makes use of feedback pathways—states of a downstream module can influence the recognition and processing of more upstream sensory inputs. This is advantageous in order to better recognize noisy or incomplete input sensory information. In previous versions of the architecture and again in the CCA6, the feedback pathways between the Input Sensory Vectors Association Modules and the Navigation Module A remain enhanced so that they can allow not just a feedback signal, but the full intermediate results from the Navigation Module A to be stored in the Input Sensory Vectors Association Modules.

In some cognitive cycles in the CCA6 there is no output signal. In certain states (for example, no actionable output from the Navigation Module A) the information in the Navigation Module A can be fed back and stored in the Input Sensory Vectors Association Modules. When this happens then in the next cognitive cycle these intermediate results will automatically be considered as the input sensory information and propagated to the Navigation Module A and operated on again. As noted above in the body of the paper, by feeding back and re-operating on the intermediate results, the Causal Cognitive Architecture can formulate and explore possible cause and effect of actions, i.e., generate causal behavior.

The Working Primitive **WPR***_t_* has been applied against the Working Navigation Map **WNM’***_t_*) producing an *action_t_* signal: *action_t_* = Nav_ModA.apply_primitive(**WPR***_t_*, **WNM’***_t_*) (82). In the previous section the *action_t_* signal was actionable, i.e., it contained the string “move*” and so via equations (84–87) it was propagated to the Output Vector Association Module A and to the Output Vector Shaping Module A where it then moved the actuators of the embodiment or sent an electronic signal in accordance with the *action* specified. However, what if the *action_t_* signal produced is not actionable, i.e., it does not contain the string “move*”? There is no indication that any actuator should be moved, or any electronic signal should be sent. In this case *action_t_* would be discarded. Another *action_t_* signal would be produced in the next cognitive cycle, and perhaps it would be actionable.

However, given the enhancement in feedback pathways between the Navigation Module A and the Input Sensory Vectors Association Modules, the Causal Cognitive Architecture can in these cases feed back the intermediate results of the Navigation Module A and store them in the Input Sensory Vectors Association Modules (88). Note that equation (88) will feedback the intermediate results (i.e., the Working Navigation Map **WNM’***_t_* if *action_t_* ≠ “move*”, i.e., the *action_t_* signal is not actionable. However, if the Working Primitive **WPR***_t_* explicitly specifies to discard the intermediate results they will not be fed back. Similarly, if the Working Primitive **WPR_t_** explicitly specifies to feed back the intermediate results they will be fed back even if they are actionable. In equation (88) the pseudocode Nav_ModA**.**feedback_to_assocn_mod(**WNM’***_t_*)specifies to feed back and store **WNM’***_t_* in the Input Sensory Vectors Association Modules.

In the next cognitive cycle, in equation (89) the pseudocode Input_Sens_Vectors_Assoc_Module**_σ_.** extract_*σ*(**WNM’***_t_*) specifies that the Working Navigation Map just fed back (i.e., **WNM’***_t_*) is to have its components extracted (i.e., effectively stored) into the best matching local navigation maps of the various Input Sensory Vectors Associations Modules in the next cognitive cycle t+1. Thus, as the cognitive cycle progresses, the actual sensory inputs will be ignored, and instead these components of the previous Working Navigation Map **WNM’***_t_* (i.e., the intermediate results from the Navigation Module A in the last cognitive cycle) are propagated towards the Navigation Module A and operated on again, perhaps by new instinctive or learned primitives in this new cognitive cycle. Feeding back the Navigation Module A’s intermediate results back to the sensory stages and then processing the intermediate results in the next cycle by the Navigation Module A, over and over again as needed, will allow a robust application of rules to be applied onto the modeled world, and allow exploration and examination of what the effect of a cause will be. As described in the text of the paper above, previous Causal Cognitive Architectures have shown that by feeding back and re-operating on the intermediate results, the architecture can generate causal behavior.

These equations in the CCA6 architecture are somewhat changed from the prior CCA5 architecture to reflect the duplication of the Navigation Modules.

(*action_t_*  ≠ “move*” and **WPR***_t_*  ≠ [“discard*”]) or **WPR***_t_* = [“feedback*”]**,**

⇒ Nav_ModA**.**feedback_to_assocn_mod(**WNM’***_t_*) (88)

⇒ ∀*_σ_* : **LNM_(_***_σ, ϓ ,t+1_***_)_ =** Input_Sens_Vectors_Assoc_Module**_σ_.**extract_*σ*(**WNM’***_t_*) (89)

| (*action_t_* ≠ “move*” and **WPR***_t_*  ≠ [“discard*”])  or **WPR***_t_* = [“feedback*”]**,**  ⇒ …. | -if *action_t_* which the Navigation Module A (e.g., Figure 5 or Figure 9) produced (82) is not actionable (i.e., does not specify an activation of an actuator or the transmission of an electronic signal) and also there is no specification by the Working Primitive **WPR***_t_* to discard *action_t_*  (i.e., the Navigation Module A should not do anything this cognitive cycle) then operations after the ⇒ symbol will occur  -if Working Primitive **WPR***_t_* specifies that the intermediate results of the Navigation Module A should be fed back, then regardless of whether *action_t_* is actionable or not, the operations after the ⇒ symbol will occur |
| --- | --- |
| ⇒Nav_ModA**.**feedback_to_assocn_mod(**WNM’***_t_*) | -pseudocode to feed back and store the intermediate results of the Navigation Module A, i.e., the Working Navigation Map **WNM’_t_** to the Input Sensory Vectors Association Modules |
| ⇒∀*_σ_* : **LNM_(_***_σ, ϓ ,t+1_***_)_ =**  Input_Sens_Vectors_Assoc_Module**_σ_.**extract_*σ*(**WNM’***_t_*) | -pseudocode that specifies that the Working Navigation Map just fed back (i.e., **WNM’***_t_* ) is to have its components extracted (i.e., effectively stored) into the best matching local navigation maps of the various Input Sensory Vectors Associations Modules of the next cognitive cycle t+1  -thus, as the cognitive cycle progresses, the actual sensory inputs will be ignored, and instead these components of the previous Working Navigation Map **WNM’***_t_* (i.e., the intermediate results from the Navigation Module A in the last cognitive cycle) will be propagated towards the Navigation Module A and operated on again |

Table A16. Explanation of Symbols and Pseudocode in Equations (88) – (89)

| **Initial Input:** | *not applicable as this is a continuation of Navigation Module A operations* |
| --- | --- |
| **Initial Output:** | **WNM’***_t_* : the current Working Navigation Map is fed back to the Input Sensory Vectors Association Modules |
| **Second Input:** | in the next cognitive cycle **LNM_(σ, ϓ ,t+1)_** for some or all of the sensory systems, derived from the fed back **WNM’***_t_* , are treated as inputs and processed again by the Navigation Module A complex (i.e., the Object Segmentation Gateway Module, the Causal Memory Module and the Navigation Module A) |
| **Second Output:** | -in the next cognitive cycle, the Navigation Module A will produce again an *action_t_* signal as per equation (82)  -if *action_t_* is actionable (i.e., an activation of an actuator or the transmission of an electronic signal) then the *action_t_* signal will be sent to the Output Vector Association Module A, as in the previous section  -if *action_t_* is not actionable then intermediate results of the Navigation Module A (i.e., **WNM’***_t_*) may be fed back again to the Input Sensory Vectors Association Modules (88, 89) |
| **Description:** | -In the previous section it was seen that the Navigation Module A applies the Working Primitive **WPR***_t_* against the current Working Navigation Map **WNM’** and produced an *action_t_*  signal which was sent to the Output Vector Association Module A and then to the external embodiment to activate an actuator or send an electronic signal.  -In this section it is seen that if *action_t_* is not actionable then intermediate results of the Navigation Module A (i.e., **WNM’***_t_*) may be fed back again to the Input Sensory Vectors Association Modules and then in the next cognitive cycle return back to the Navigation Module A where it can be operated on again.  -By feeding back and re-operating on the intermediate results, the architecture can generate causal behavior.  -Applies to the CCA5 version of the architecture (Figure 1) but there is only a single Navigation Module. Applies CCA6 (Figure 5) and the CCA7 (Figure 9) versions of the Causal Cognitive Architecture where there is a separate Navigation Module A and B (with the latter plural in the CCA7). |

Table A17. Summary of the Feedback Operations from the Navigation Module A per Equations (88) – (89)

**A.9 Analogical Problem Solving**

This section of Appendix A applies to the CCA5 (Figure 1), CCA6 (Figure 5) and the CCA7 (Figure 9) versions of the Causal Cognitive Architecture. In the CCA5 version of the architecture its sole Navigation Module should be considered the same as Navigation Module A below.

In the last section above, it was shown how with enhanced feedback pathways from the Navigation Module A to the Input Sensory Vectors Association Modules and some small changes, if there is no actionable output from the Navigation Module A, then the Navigation Module A can feed back the Working Navigation Map **WNM’**. Without many changes to the architecture, when this happens then in the next cognitive cycle these intermediate results will automatically be considered as the input sensory information and propagated back to the Navigation Module A and operated on again. As discussed in the text of the paper above, it has previously been shown that in this type of Causal Cognitive Architecture that by feeding back and re-operating on the intermediate results, the architecture is sometimes able to formulate and explore possible cause and effect of actions, i.e., generate causal behavior.

However, often the combinations of sensory inputs leading to **WNM’** and the chosen instinctive or learned primitives leading to **WPR’** which operates on the Working Navigation Map **WNM’**, does not give a causally related or even a useful output.

This section describes a small, evolutionarily plausible modification of the feedback algorithm from the Navigation Module A to the Input Sensory Vectors Association Modules which readily emerges. The result is the ready generation of analogical results that may be more useful than simply feeding back and returning the intermediate results unchanged in the next cognitive cycle.

In the last section above it was seen in (88) that if the *action*_t_ which is produced by the Navigation Module A (i.e., *action_t_* = Nav_ModA.apply_primitive(**WPR***_t_*, **WNM’***_t_*) (82)) is not actionable (i.e., there is no actuator to move or no electronic signal to send) then the Navigation Module A feeds back what will now be considered intermediate results of the Navigation Module A (i.e., **WNM’***_t_*) for storage in the Input Sensory Vectors Association Modules. (A property which can easily emerge by simply enhancing feedback pathways in the architecture.) In the next cognitive cycle, **WNM’***_t_* is treated as the input signal (89) and thus automatically propagated back to the Navigation Module A where it can be operated on. Perhaps different instinctive or learned primitives operating on **WNM’***_t_*_-1_ (“*t -* 1” just means it is from the previous cognitive cycle) or the transformation of the data, will produce a useful, actionable output during this cognitive cycle. Equations (88) and (89) are reproduced again below. Note that the Working Primitive **WPR***_t_* can specify to discard results (i.e., **WPR***_t_*  = [“discard*”]) after a cognitive cycle (i.e., nothing will happen except wait for the next cognitive cycle) or it can specify to feed back the results of the Navigation Module A even if the action is actionable (i.e., **WPR***_t_* = [“feedback*”]).

(*action_t_*  ≠ “move*” and **WPR***_t_*  ≠ [“discard*”]) or **WPR***_t_* = [“feedback*”]**,**

⇒ Nav_ModA.feedback_to_assocn_mod(**WNM’***_t_*) (88)

⇒ ∀*_σ_* : **LNM_(_***_σ, ϓ ,t_***_)_ =** Input_Sens_Vectors_Assoc_Module**_σ_.**extract_*σ*(**WNM’***_t_*_-1_) (89)

These small changes in the architecture are sufficient to easily allow the emergence of a different type of feedback by the Navigation Module A. The feedback shown in (88) and (89) will no longer occur by default. However, if the Working Primitive **WPR***_t_* specifically signals “feedback” then the same feedback algorithm as before (i.e., described above (88, 89)) will occur. Equations (88, 89) are replaced by (88a, 89) to specify this condition where the previously described feedback algorithm will still occur:

**WPR***_t_* = [“feedback*”]**,**

⇒ Nav_ModA.feedback_to_assocn_mod(**WNM’***_t_*) (88a)

⇒ ∀*_σ_* : **LNM_(_***_σ, ϓ ,t_***_)_ =** Input_Sens_Vectors_Assoc_Module**_σ_.**extract_*σ*(**WNM’***_t_*_-1_) (89)

The new default feedback algorithm which will occur is now specified as starting in (90). If the *action_t_* signal produced by the Navigation Module A (i.e., *action_t_* = Nav_ModA.apply_primitive(**WPR***_t_*, **WNM’***_t_*) (82)) is not actionable (*action_t_*  ≠“ move*”—there is no actuator to move or no electronic signal to send) then fed back are what will now be considered intermediate results of the Navigation Module A (i.e., **WNM’***_t_*) for storage in the Input Sensory Vectors Association Modules (90). This is the same effect as occurred before and still occurs in (88a). (Note also from (90) that if the Working Primitive **WPR***_t_*  specifies “analogical” feedback then this will occur even if the *action_t_* signal is actionable. Similarly, if **WPR***_t_*  specifies to “discard” the results, then nothing will happen except wait for the next cognitive cycle.) Thus, **WNM’***_t_*  is sent (i.e., fed back) to the Input Sensory Vectors Association Modules as before (90).

**WNM’***_t_*  is also sent to the Causal Memory Module (91). Sending **WNM’**_t_ to the Causal Memory Module (91) triggers Causal_Mem_Mod**.**match_best_map (**WNM’***_t_*). This pseudocode matches **WNM’***_t_* against the multisensory navigation maps stored in the Causal Memory Module, and assigns this best matching navigation map as the new **WNM’***_t_*  value. This matching algorithm is similar to other matching algorithms for navigation maps used by the architecture. **WNM’***_t_*  is also sent to the TempMap region of the Navigation Module A. This happens automatically.

Results of almost all operations from the Navigation Module A and the Causal Memory Module are routinely copied to the TempMap region. Often the information in the TempMap region (i.e., “TempMapA”, the TempMap region in Navigation Module A) is not used and it is overwritten in the next cognitive cycle. This is the case now—the contents of the TempMap region will not be used. However, in later steps in equations (92, 93) the TempMap region is indeed used. Note that in the equations the TempMap region is defined as an array **TempMap** which acts like an ordinary navigation map (66).

In (92) the pseudocode Nav_ModA.use_linkaddress1_map(**WNM’***_t_*)activates in the Causal Memory Module the navigation map which the most recently used linkaddress used in the past by the current Working Navigation Map **WNM’***_t_* points to. As shown in (92) this result of the Causal Memory Module is automatically copied to **TempMap***_t_*. (As mentioned above results of the Navigation Module A and the Causal Memory Module are routinely and automatically copied to **TempMap**.)
 (Note that other algorithms besides use_linkaddress1_map()are possible. For example, rather than the most recently used linkaddress, a number of the linkaddresses used by this navigation map in the past can be explored and one particular linkaddress chosen, and so on. Similarly, most recently produced *action*’s can also be explored.)

In (93) the pseudocode Nav_ModA.subtract(**TempMap**, **WNM’***_t_*) the navigation map **WNM’***_t_* is subtracted from **TempMap**. (The navigation maps are arrays and can be treated as such via simple array operations.) The result becomes the new Working Navigation Map **WNM’***_t_* (93).

The next cognitive cycle then occurs. At this point, the contents of the **WNM’***_t_* Working Navigation Map in the Navigation Module A contains the difference between the previous **WNM’***_t_* and retrieved ***linkaddress*** ***χ’*** Navigation Map stored in **TempMap**. At this point, the Input Sensory Vectors Association Modules contain the original Working Navigation Map **WNM’***_t_*_-1_ (*t*-1 just means something from the previous cognitive cycle) which was fed back and stored here. Thus, during this cognitive cycle, the actual sensory inputs will be ignored, and the previous **WNM’***_t_*_-1_ will be propagated towards the Navigation Module A.

To indicate continuation of the operations on associated equations (90–93), in (94) it is specified “(*action_t-1_* ≠ “move*” or **WPR***_t_*_-1_ = [“analogical*”]) and **WPR***_t-_*_1_ ≠ [“discard*”] and **WPR***_t_***_-_**_1_≠ [“feedback*”] )**,**” and then specified to run this specific pseudocode: **WNM’***_t_* = Nav_ModA.retrieve_and_add_vector_assocn()(94). Rather than propagate **WNM’***_t_*_-1_ towards the Navigation Module A and then it replace the existing **WNM’***_t_* and is operated on by the current Working Primitive **WPR’** , as occurred automatically with the previous feedback algorithm above, the pseudocode retrieve_and_add_vector_assocn() specifies that **WNM’***_t_*_-1_ should not replace but simply be added to the existing **WNM’***_t_* .

Now the original Working Navigation Map **WNM’***_t_* in the Navigation Module A also contains the action that occurred in the past of a similar Working Navigation Map in a possible analogical situation.

The equations below are somewhat changed from the prior CCA5 architecture to reflect the duplication of the Navigation Modules. As noted above, while applying more now the CCA6 and CCA7 versions of the architecture, for the CCA5 version its sole navigation module should be considered as Navigation Module A.

( (*action_t_*  ≠“ move*” or **WPR***_t_* = [“analogical*”]) and **WPR***_t_*  ≠ [“discard*”] and **WPR***_t_* ≠ [“feedback*”] )**,**

⇒Nav_ModA.feedback_to_assocn_mod(**WNM’***_t_*) (90)

⇒ **WNM’***_t_* = Causal_Mem_Mod**.**match_best_map(**WNM’***_t_*) (91)

⇒ **TempMap***_t_* = Nav_ModA.use_linkaddress1_map(**WNM’***_t_*) (92)

⇒ **WNM’_t_** = Nav_ModA.subtract(**WNM’***_t_* , **TempMap**) (93)

( (*action_t-1_* ≠ “move*” or **WPR***_t_*_-1_ = [“analogical*”]) and **WPR***_t-_*_1_ ≠ [“discard*”] and **WPR***_t_***_-_**_1_≠ [“feedback*”] )**,**

⇒ **WNM’***_t_* = Nav_ModA.retrieve_and_add_vector_assocn() (94)

| **WPR***_t_* = [“feedback*”]**,**  ⇒ …. | -replaces equation (88) (see Table A17)  -previously described feedback algorithm now only occurs if the Working Primitive **WPR***_t_* specifies it (i.e., **WPR***_t_* = [“feedback*”]**,**). |
| --- | --- |
| ⇒Nav_ModA.feedback_to_assocn_mod(**WNM’***_t_*) | -previously described in Table A17  -applied to equations (88)/(88a) or (90)  -pseudocode to feed back and store the intermediate results of the Navigation Module A, i.e., the Working Navigation Map **WNM’***_t_* in the Input Sensory Vectors Association Modules (Figure 5 or Figure 9) |
| ⇒∀*_σ_* : **LNM_(_***_σ, ϓ ,t_***_)_ =**  Input_Sens_Vectors_Assoc_Module**_σ_.**extract_*σ*(**WNM’***_t-1_*) | -previously described in Table A17  -applies to equation (89)  -pseudocode that specifies that the Working Navigation Map just fed back (i.e., **WNM’***_t-1_* which is **WNM’***_t_* of the previous cognitive cycle) is to have its components extracted (i.e., effectively stored) into the best matching local navigation maps of the various Input Sensory Vectors Associations Modules  -thus, as the cognitive cycle progresses, the actual sensory inputs will be ignored, and instead these components of the previous Working Navigation Map **WNM’***_t-1_* (i.e., the intermediate results from the Navigation Module A in the last cognitive cycle) will be propagated towards the Navigation Module A and operated on again  -see Figure 5 (CCA6 version) or Figure 9 (CCA7 version of the architecture) for overview of modules and interconnections |
| ( (*action_t_*  ≠“ move*” or **WPR***_t_* = [“analogical*”]) and **WPR***_t_*  ≠ [“discard*”] and **WPR***_t_* ≠ [“feedback*”] )**,**  ⇒ | -**if** the *action_t_* signal produced by the Navigation Module A (i.e., *action_t_* = Nav_ModA.apply_primitive(**WPR***_t_*, **WNM’***_t_*) (82)) is not actionable (*action_t_*  ≠“ move*”—there is no actuator to move or no electronic signal to send)  -**or** if the Working Primitive **WPR***_t_*  specifies “analogical” feedback (regardless if the *action_t_* signal is actionable)  -**and** the Working Primitive **WPR***_t_*  does not specify to “discard” the results (regardless if the *action_t_* signal is actionable, if “discard” is specified then nothing happens except to wait for the next cognitive cycle)  -**and** the Working Primitive **WPR***_t_*  does not specify to use the previous “feedback” pseudocode (i.e., equations (88, 89)) (regardless if the *action_t_* signal is actionable)  -**then** equations/pseudocode which follows—which are equations (90,91,92, 93)—will be run |
| ⇒Nav_ModA.feedback_to_assocn_mod(**WNM’***_t_*) | -equation (90) is the same pseudocode as equations (88)/ (88a)  -pseudocode to feed back and store the intermediate results of the Navigation Module A, i.e., the Working Navigation Map **WNM’***_t_* in the Input Sensory Vectors Association Modules |
| ⇒Causal_Mem_Mod**.**match_best_map(**WNM’***_t_*)  🡪 **WNM’***_t_* | **-WNM’***_t_*  is also sent to the Causal Memory Module (91)  -sending **WNM’**_t_ to the Causal Memory Module (91) triggers Causal_Mem_Mod**.** match_best_map (**WNM’***_t_*)  -this pseudocode matches **WNM’***_t_*  against the multisensory navigation maps stored in the Causal Memory Module, and assigns this best matching navigation map as the new **WNM’***_t_* value |
| ⇒Nav_ModA.use_linkaddress1_map(**WNM’***_t_*)  🡪 **TempMap***_t_* | -the pseudocode Nav_ModA.use_linkaddress1_map(**WNM’***_t_*) activates in the Causal Memory Module the navigation map which the most recently used linkaddress used in the past by the current Working Navigation Map **WNM’***_t_* points to (92)  -the result of the Causal Memory Module is automatically copied to **TempMap***_t_*.  -as noted earlier, in the CCA6 version of the architecture, results of the Navigation Module A and the Causal Memory Module are routinely and automatically copied to **TempMap** |
| ⇒Nav_ModA.subtract(**WNM’***_t_*, **TempMap**) (93)  🡪 **WNM’***_t_* | -the pseudocode Nav_ModA.subtract(**TempMap**, **WNM’***_t_*)subtracts the navigation map **WNM’***_t_*  from the navigation map in **TempMap** (93)  -the result becomes the new Working Navigation Map **WNM’***_t_* |
| ( (*action_t-1_* ≠ “move*” or **WPR***_t_*_-1_ = [“analogical*”]) and **WPR***_t-_*_1_ ≠ [“discard*”] and **WPR***_t_***_-_**_1_≠ [“feedback*”] )**,**  ⇒ | -“*t-1*” simply refers to the cognitive cycle before this one, i.e., to the values from the previous cognitive cycle  -if true then the pseudocode of equation (94) is run  -if equation (90)’s conditions are true and its pseudocode is run, then this will also be true (exact same conditions except referring to the previous cognitive cycle), thus equations (90) to (94) are run one after another |
| ⇒Nav_ModA.retrieve_and_add_vector_assocn() (94)  🡪 **WNM’***_t_* | -rather than propagate **WNM’***_t_*_-1_ (which is in the Sensory Input Vectors Associations Modules, and will utilized in the new cognitive cycle, rather than the actual sensory inputs) towards the Navigation Module A and then automatically replace the existing **WNM’***_t_* , the pseudocode specifies that **WNM’***_t_*_-1_ should not replace but simply be added to the existing **WNM’***_t_*  -thus, now the original Working Navigation Map **WNM’***_t_* in the Navigation Module A will contain the action or navigation map that occurred in the past of a similar Working Navigation Map in a possible analogical situation |

Table A18. Explanation of Symbols and Pseudocode in Equations (88a) – (94)

| **Input:** | *not applicable as this is a continuation of Navigation Module A operations* |
| --- | --- |
| **Temporary**  **Inputs/Outputs occurring:** | ***note:*** *to distinguish between the several different navigation maps which are considered the current Working Navigation Map* ***WNM’*** *at some point, descriptive suffixes are added to* ***WNM’***  *(however, note that there is only one navigation map in the Navigation Module A at one time designated as* ***WNM’****; the descriptive suffixes are for the reader, they do not exist in the architecture)*  **-****WNM’***_t_*-*original*: the original current Working Navigation Map is fed back to the Input Sensory Vectors Association Modules (90)  **- WNM’***_t_*-*original*: the original current Working Navigation Map is propagated to the Causal Memory Module and the resultant best matching navigation map is sent back to the Navigation Module A and replaces **WNM’***_t_*-*original* with **WNM’***_t_*-*best_match*  (91)  **- WNM’***_t_*-*best_match*-*linkaddress1*: its most recently used ***linkaddress*** (i.e., pointing and accessing another navigation map) is sent to the Causal Memory Module and puts the retrieved (actually just activated) navigation map into **TempMap** (92)  -**TempMap**: holding the retrieved navigation map **WNM’***_t_*-*best_match*-*linkaddress1* (92), which will then subtract the current Working Navigation Map **WNM’***_t_*-*best_match*  and the result will be the new Working Navigation Map **WNM’***_t_*-*difference* (93)  -in the next cognitive cycle **LNM_(σ, ϓ ,t)_** for some or all of the sensory systems, derived from the fed back **WNM’***_t-1_*-*original*, are treated as inputs and added to the existing Working Navigation Map **WNM’***_t_*-*difference*  resulting in **WNM’***_t_*-*analogical*  (94) |
| **Output:** | *not applicable as this is a continuation of Navigation Module A operations* |
| **Description:** | -Earlier it was seen that the Navigation Module A applies the Working Primitive **WPR***_t_* against the current Working Navigation Map **WNM’** and produced an *action_t_*  signal which was sent to the Output Vector Association Module A and then to the external embodiment to activate an actuator or send an electronic signal.  -In the previous section it was seen that if *action_t_* is not actionable then intermediate results of the Navigation Module A (i.e., **WNM’***_t_*) may be fed back again to the Input Sensory Vectors Association Modules and then in the next cognitive cycle return back to the Navigation Module A where it can be operated on again. By feeding back and re-operating on the intermediate results, the architecture can generate causal behavior.  -In this section shown is a different “analogical” feedback algorithm to use if *action_t_* is not actionable.  -The analogical feedback algorithm in this section starts off similarly to the previous feedback algorithm, but rather than simply feeding back the Working Navigation Map **WNM’***_t_* unchanged to be processed further in the next cognitive cycle, it feeds back and constructs a navigation map that occurred in the past of a similar Working Navigation Map **WNM’***_t_*  (which can call “**WNM’***_t_*-*analogical*” to distinguish it from the other navigation maps that are set as **WNM’***_t_* at different points).  -**WNM’***_t_*-*analogical* may be more likely in a possible analogical situation. This navigation map can then be processed further in the next cognitive cycle.  -By using a possibly analogical navigation map it makes it more likely the navigation map which becomes the Working Navigation Map **WNM***_t_* in the next cognitive cycle, and upon which possibly another (or possibly the same) **WPR***_t_* will be applied, that an actionable *action_t_* will result since it is using a navigation map that was tried in the past.  **-**At the present, the algorithm underlying the pseudocode Nav_ModA.use_linkaddress1 _map(**WNM’***_t_*)(92) is simple and straightforward. However, in future versions more sophisticated algorithms for this pseudocode could choose better among matching navigation maps that were more successful in the past in producing an actionable *action*.  -The next section will show that induction by analogy is actually occurring in this process.  - Applies to the CCA5 (Figure 1), CCA6 (Figure 5) and the CCA7 (Figure 9) versions of the Causal Cognitive Architecture; in the CCA5 version of the architecture its sole Navigation Module should be considered the same as Navigation Module A |

Table A19. Summary of the Analogical Feedback Operations from the Navigation Module A per Equations (88a) – (94)

**A.10 Induction by Analogy**

This section of Appendix A applies to the CCA5 (Figure 1), CCA6 (Figure 5) and the CCA7 (Figure 9) versions of the Causal Cognitive Architecture. In the CCA5 version of the architecture its sole Navigation Module should be considered the same as Navigation Module A.

Below it is shown that the movement and comparison of navigation maps in equations (90 – 94) allows induction by analogy to occur. These equations are re-written using the more descriptive terms for the Working Navigation Maps used in Table A19—to distinguish between the several different navigation maps which are considered the current Working Navigation Map **WNM’** at some point, descriptive suffixes are added to **WNM’**. These descriptive suffixes are for the reader, they do not exist in the architecture.

( (*action_t_*  ≠“ move*” or **WPR***_t_* = [“analogical*”]) and **WPR***_t_*  ≠ [“discard*”] and **WPR***_t_* ≠ [“feedback*”] )**,**

⇒Nav_ModA.feedback_to_assocn_mod(**WNM’***_t_*-*original*) (95) *(from 90)*

⇒ **WNM’***_t_*-*best_match*  = Causal_Mem_Mod**.**match_best_map(**WNM’***_t_*-*original*) (96) *(from 91)*

⇒ **TempMap***_t_* = Nav_ModA.use_linkaddress1_map(**WNM’***_t_*-*best_match*) (97) *(from 92)*

⇒ **WNM’***_t_*-*difference*  = Nav_ModA.subtract(**WNM’***_t_*-*best_match* , **TempMap***_t_*) (98) *(from 93)*

( (*action_t-1_* ≠ “move*” or **WPR***_t_*_-1_ = [“analogical*”]) and **WPR***_t-_*_1_ ≠ [“discard*”] and **WPR***_t_***_-_**_1_≠ [“feedback*”] )**,**

⇒ **WNM’***_t_*-*analogical*  = Nav_ModA.retrieve_and_add_vector_assocn() (99) *(from 94)*

Consider a definition of induction by analogy. There are two variables **x** and **y**. Variable **x** has properties P_1_, P_2_, P_3_, P_4_, … P_n_  (100). Variable **y** also has properties P_1_, P_2_, P_3_, P_4_, … P_n_  (101). It happens that variable **y** has another property N (102). Therefore in (103) it can be concluded by induction by analogy that variable **x** also has property N.

In (95) consider **WNM’***_t_*-*original* as variable **x**, or perhaps as navigation map **x**. It is desired to know what this navigation map **x** will do next, i.e., which navigation map will it call. Consider variable **y**, or perhaps named as navigation map **y**, as referring to (96) **WNM’***_t_*-*best_match*. It is the best matching navigation map to navigation map **x** and thus assumed it will share many properties. Next there is exploration of what navigation map **y** does next (i.e., what navigation map does the ***linkaddress*** chosen link to). Navigation map **y** calls the navigation map in **TempMap** (97) and that the difference between navigation map **y** and **TempMap** is **WNM’***_t_*-*difference* (98). Thus, consider this difference, i.e., **WNM’***_t_*-*difference* to be property N. Since navigation map **y** has property N, therefore by induction by analogy, it can be said that navigation map **x** also has property N (103). Thus, add property N, which is actually **WNM’***_t_*-*difference*, to navigation map **x**, which is actually **WNM’***_t_*-*original*, producing the result of navigation map **x** with property N as being **WNM’***_t_*-*analogical*  (99).

P_1_**x &** P_2_**x & …** P_n_**x** (100)

P_1_**y &** P_2_**y & …** P_n_**y** (101)

N**y** (102)

**∴** N**x □** (103)

It is important to note that analogical problem solving is not a separate module in the architecture (for example, to be used when solving intelligence tests or difficult problems) but rather part of the core mechanism of day-to-day functioning of the architecture. These equations remain largely unchanged in the CCA6 architecture from the prior CCA5 architecture although there are small modifications to account for the duplicate Navigation Module. Note that equations (i) to (ix) in the text of paper above are easy-to-read versions of equations (95) to (103).

| ( (*action_t_*  ≠“ move*” or **WPR***_t_* = [“analogical*”]) and **WPR***_t_*  ≠ [“discard*”] and **WPR***_t_* ≠ [“feedback*”] )**,**  ⇒ | -see Table A18  -see Figure 5 (CCA6 version) or Figure 9 (CCA7 version of the architecture) for overview of modules and interconnections |
| --- | --- |
| ⇒Nav_ModA.feedback_to_assocn_mod(**WNM’***_t_*-*original*) (95) | -see Table A18  - **WNM’***_t_*-*original* – the current Working Navigation Map  **WNM’***_t_* which is given the label ‘original’ for better understanding of what is happening |
| ⇒Causal_Mem_Mod**.**match_best_map(**WNM’***_t_*-*original*) (96)  🡪  **WNM’***_t_*-*best_match* | -see Table A18  **WNM’***_t_*-*original* – the current Working Navigation Map  **WNM’***_t_* which is given the label ‘original’ for better understanding of what is happening |
| ⇒Nav_ModA.use_linkaddress1_map(**WNM’***_t_*-*best_match*) (97)  🡪 **TempMap***_t_* | -see Table A18  **-WNM’***_t_*-*best_match* – the current Working Navigation Map  **WNM’***_t_* which is given the label ‘best-match’ for better understanding of what is happening |
| ⇒Nav_ModA.subtract(**WNM’***_t_*-*best_match* , **TempMap***_t_*) (98)  🡪  **WNM’***_t_*-*difference* | -see Table A18  - **WNM’***_t_*-*difference*  – the current Working Navigation Map  **WNM’***_t_* which is given the label ‘difference’ for better understanding of what is happening |
| ( (*action_t-1_* ≠ “move*” or **WPR***_t_*_-1_ = [“analogical*”]) and **WPR***_t-_*_1_ ≠ [“discard*”] and **WPR***_t_***_-_**_1_≠ [“feedback*”] )**,** ⇒ | -see Table A18 |
| ⇒ **WNM’***_t_*-*analogical*  = Nav_ModA.retrieve_and_add_vector_assocn() (99)  🡪  **WNM’***_t_*-*analogical* | -see Table A18  - **WNM’***_t_*-*analogical*  – the current Working Navigation Map  **WNM’***_t_* which is given the label ‘analogical’ for better understanding of what is happening |
| P_1_**x &** P_2_**x & …** P_n_**x** (100) | -explanation of definition of induction by analogy  - variable (or navigation map) **x** has properties P_1_, P_2_, P_3_, P_4_, … P_n_ |
| P_1_**y &** P_2_**y & …** P_n_**y** (101) | -explanation of definition of induction by analogy  -variable (or navigation map) **y** also has properties P_1_, P_2_, P_3_, P_4_, … P_n_ |
| N**y** (102) | -explanation of definition of induction by analogy  - variable (or navigation map) **y** also has property N |
| **∴** N**x □** (103) | -explanation of definition of induction by analogy  - thus, can conclude by induction by analogy that variable (navigation map) **x** also has property N |

Table A20. Explanation of Symbols and Pseudocode in Equations (95) – (103)

| **Input:** | *not applicable as this is a continuation of Navigation Module A operations* |
| --- | --- |
| **Output:** | - **WNM’***_t_*-*analogical*  (99) |
| **Description:** | -See Table A18. Equations (95 – 99) are re-write of equations (90 – 94) using the more descriptive terms for the Working Navigation Maps. These descriptive suffixes are for the reader, they do not exist in the architecture.  -To distinguish between the several different navigation maps which are considered the current Working Navigation Map **WNM’** at some point, descriptive suffixes are added to **WNM’** listed above.  - Equations (100 – 103) consider a definition of induction by analogy applied to the operations performed on the Working Navigation Maps.  - Applies to the CCA5 (Figure 1), CCA6 (Figure 5) and the CCA7 (Figure 9) versions of the Causal Cognitive Architecture; in the CCA5 version of the architecture its sole Navigation Module should be considered the same as Navigation Module A |

Table A21. Summary of the Analogical Feedback Operations from the Navigation Module A per Equations (95) – (103)

**A.11 Grounding**

This section of Appendix A applies to the CCA5 (shown in Figure 1), the CCA6 (Figure 5) and the CCA7 (Figure 9) versions of the Causal Cognitive Architecture.

Like the prior CCA5 architecture, the CCA6 architecture provides a solution to the grounding problem—information enters the system through experiential sensory systems. Information will automatically be mapped to real-world sensations and actions. No information is kept as an isolated symbol—*everything* in the CCA6 is within a navigation map linked to other navigation maps.

A *feature* was defined initially in (34). Cells in navigation maps can contain *feature*s, *procedure*s, or ***linkaddresses***. Below equation (104) defines a *grounded_feature* as being any *feature* such that the *feature* is in some sensory system Local Navigation Map ***all_LNMs_χ_*** and that the *feature* also was present in some sensory system array **S***_σ,t_* (1–7) represented by ***s***(*t*) (9). This implies that the CCA6 experientially acquired this *feature*, and it is in its navigation maps associated with other sensory inputs and possibly associated with previous or upcoming *feature*s. The symbol grounding problem is largely resolved by grounding symbols with their real-world sensations, interactions, and linkages.

However, the second part of equation (104) deals with the case where information has been acquired by electronic transfer or by the equivalent of human memorization, or possibly an experiential acquisition of the *feature* was poor, and grounding is poor. A *feature* is considered as a *grounded_feature* if the *feature* is present in a Working Navigation Map **WNM’***_t_* and in the previous cognitive cycle the analogical feedback mechanism was used. Note that if in the previous cognitive cycle (*t-1*) the action was not actionable, i.e., *action_t-1_* ≠ “move*”, then the analogical feedback mechanism will occur, or if the previous Working Primitive **WPR***_t_*_-1_ specified an analogical feedback loop, then the analogical feedback mechanism will occur.

Equation (105) essentially states that any cell in any of the addressable navigation maps (e.g., it will not include, for example, the specialized navigation maps in the Sequential/Error Correcting Module) will either contain a *grounded_feature* or contain a ***linkaddress*** pointing to another cell which most likely will be in another navigation map, or else the cell has no features.

With regard to accepting as grounded a ***linkaddress*** pointing to another cell/navigation map, it is assumed that there are so many links pointing to so many other navigation maps in a chain of ***linkaddresses*** that if a cell points to another cell most likely in another navigation map, it will be grounded there by association with some valid grounding condition (e.g., sensations, actions, etc.) or else point to another navigation map where it will be ground there, and so on. There may be differences in the quality of the grounding as such, but it will always be grounded to some extent.

These equations remain largely unchanged in the CCA6 architecture from the prior CCA5 architecture.

*grounded_feature* = ∀*_feature_* : ( *feature* ∈ ***all_LNMs_χ_*** AND *feature* ∈ ***s***(*t*) )

OR

∀*_feature_* : ( ( *feature* ∈ **WNM’***_t_*  AND *action_t-1_* ≠ “move*” AND **WPR***_t_*_-1_ ≠ [“feedback*”]) OR
**WPR***_t_*_-1_ = [“analogical*”] ) (104)

∀***_χ,t_*** : ***all_navmaps_χ,t_* =** *grounded_feature* OR link*(****all_navmaps* _χ_*_,t_***) **≠** [ ] OR ***cellfeatures_χ,t_* =** [ ] (105)

| *feature* | - *feature* ∈ R (34)  - some arbitrary real number representing a *feature* modality and value  - for example, *feature*_13,(_*_χ_*_modcode=*Causal_Memory_Mod*,_ *_mapno_*_=3456,2,3,4)_ would be *feature* number 13 in the cell x=2,y=3,z=4 in map number 3456 in the Causal Memory Module; its value, for example, could represent a visual line |
| --- | --- |
| *grounded_feature* | - any *feature* such that the *feature* is in some sensory system Local Navigation Map ***all_LNMs_χ_***  and that the *feature* also was present in some sensory system array **S***_σ,t_* (1–7) represented by ***s***(*t*) (9)  - or if the *feature* has been acquired by electronic transfer or by the equivalent of human memorization then consider a *feature* as a *grounded_feature* if the *feature* is present in a Working Navigation Map **WNM’***_t_*  and in the previous cognitive cycle the analogical feedback mechanism was used  -see Figure 1( CCA5 version) Figure 5 (CCA6 version) or Figure 9 (CCA7 version of the architecture) for overview of modules and interconnections |
| ∀*_feature_* : ( *feature* ∈ ***all_LNMs_χ_*** AND *feature* ∈ ***s***(*t*) )  🡪 *grounded_feature* | - any *feature* such that the *feature* is in some sensory system Local Navigation Map ***all_LNMs_χ_***  and that the *feature* also was present in some sensory system array **S***_σ,t_* (1–7) represented by ***s***(*t*) (9) |
| ∀*_feature_* : ( ( *feature* ∈ **WNM’***_t_*  AND *action_t-1_* ≠ “move*” AND **WPR***_t_*_-1_ ≠ [“feedback*”]) OR  **WPR***_t_*_-1_ = [“analogical*”] )    🡪 *grounded_feature* | - for example, a *feature* that has been acquired by electronic transfer or by the equivalent of human memorization, then the analogical feedback mechanism will occur (when the *feature* is considered), and consider that *feature* as a *grounded_feature*  - although the older feedback mechanism *may* result in grounding the feature adequately, often it won’t, thus requiring utilization of the analogical feedback mechanism to ensure grounding |
| ***cellfeatures_χ,t_* =** [ ] | *-****cellfeatures_χ_****_,t_* are all the features within a given cell ***χ***  (38)  -thus, ***cellfeatures_χ,t_* =** [ ] means there are no features in that cell at address ***χ*** |
| ∀***_χ,t_*** : ***all_navmaps_χ,t_* =** *grounded_feature* OR link*(****all_navmaps* _χ_*_,t_***) **≠** [ ] OR ***cellfeatures_χ,t_* =** [ ] | - any cell in any of the addressable navigation maps will either contain a *grounded_feature*, or contain a ***linkaddress*** pointing to another cell which most likely will be in another navigation map, or else the cell has no features |

Table A22. Explanation of Symbols and Pseudocode in Equations (104) – (105)

| **Input:** | *not applicable as this is a continuation of Navigation Module A operations* |
| --- | --- |
| **Output:** | *not applicable as this is a continuation of Navigation Module A operations* |
| **Description:** | -These equations specify that for every addressable navigation map in the Causal Cognitive Architecture *feature*s can be considered to be grounded or to be used in a grounded fashion.  - Applies to the CCA5 (shown in Figure 1), the CCA6 (Figure 5) and the CCA7 (Figure 9) versions of the architecture |

Table A23. Summary of the Grounding Requirements with the Architecture per Equations (104) – (105)

**A.12 Simple Language Generation**

This section of Appendix A applies to the CCA6 (Figure 5) and the CCA7 (Figure 9) versions of the Causal Cognitive Architecture.

The generation of simple language (i.e., as opposed to full compositional language) was discussed in the previous Causal Cognitive Architectures, including the previous CCA5 one. To a large extent, some way of communicating what is in a navigation map(s) from one CCA6 (or CCA7) embodiment to another CCA6(or CCA7) embodiment is required. In the CCA5 architecture this was accomplished with a straightforward instinctive primitive named apply_primitive_nav_to_protolang() .

In the CCA6 or CCA7 version of the architecture, while compositional language comprehension and behavior makes use of Navigation Module B, as was illustrated in the text and figures of the main body of the paper, language expression remains simple (i.e., essentially a simple read-back of what is on the navigation maps is being communicated) and still remains generated by Navigation Module A. In future versions of the architecture, full compositional generation of language is expected.

In (106) Nav_ModA.apply_primitive_nav_to_protolang (**WPR***_t_*, **WNM’***_t_*) produces an output action *action_t_* . As before, per equations (83 – 87), *action_t_* is propagated from the Navigation Module A to the Output Vector Association Module A. As before, there is motion correction of the output signal with interactions with the Sequential/Error Correcting Module, and then the output signal is sent to the Output Vector Association Module A and then on to the actual output actuators.

The equation below remains largely unchanged in the CCA6 or CCA7 versions of architecture from the prior CCA5 architecture, although it now specifies the Navigation Module being used.

*action_t_*= Nav_ModA.apply_primitive_nav_to_protolang (**WPR***_t_*, **WNM’***_t_*) (106)

| Nav_ModA.apply_primitive_nav_to_protolang(**WPR***_t_*, **WNM’***_t_*)  🡪 *action_t_* | -Nav_Mod refers to the Navigation Module A (Figure 5 or Figure 9)  -apply_primitive_nav_to_protolang (**WPR***_t_*, **WNM’***_t_*) is pseudocode for an algorithm which applies the Working Primitive **WPR**_t_ to the Working Navigation Map **WNM’**, such that the current **WNM’** and sequence of **WNM’** Working Navigation Maps preceding it (i.e., what happened? why did it happen?) are converted into a simple proto-language which is represented in the *action_t_* value  -this is a simple read-back (limited verbs used) of the navigation maps  - the *action_t_* value will be transformed into an output signal that activates the appropriate actuators to communicate this simple proto-language communication to another CCA6 embodiment or a human  - see Table A14 for more details of converting WPR*_t_* and WNM’*_t_* into an actionable output, which in this case represents a communication |
| --- | --- |

Table A24. Explanation of Symbols and Pseudocode in Equation (106)

| **Input:** | **WPR***_t_* : the Working Primitive **WPR** is the primitive that will be applied against the Working Navigation Map **WNM’** in the Navigation Module A, and with regard to (106) will involve transforming the recently stored (in the Causal Memory Module) Working Navigation Maps **WNM’**s into an explanation and communication of what happened and why it happened, as well as possibly communication of actions, requests, or concepts  **WNM’***_t_* : the current Working Navigation Map which is derived from the processed sensory inputs or analogic feedback results |
| --- | --- |
| **Output:** | *action_t_* : a signal to move some actuator or send an electronic signal  e.g., **<move right>** e.g., <**sound 2000Hz>**  it is sent to the Output Vector Association Module A where more detailed instructions are created to effect the required actuator outputs |
| **Description:** | -The Navigation Module A applies the Working Primitive **WPR***_t_* against the current Working Navigation Map **WNM’** in the Navigation Module A, and with regard to (106) will involve transforming the recently stored (in the Causal Memory Module) Working Navigation Maps **WNM’**s into an explanation and communication of what happened and why it happened, as well as possibly communication of actions, requests, or concepts.  -Applies to the CCA6 or CCA7 versions of the architecture |

Table A25. Summary of the Operation of Simple (Non-Compositional) Language Generation per Equation (106)

A.13 Navigation Module B

This section of Appendix A applies to the CCA6 (Figure 5) and CCA7 (Figure 9) versions of the Causal Cognitive Architecture.

As seen in Figure 5 there are now in the CCA6 two Navigation Modules—Navigation Module A and Navigation Module B (as opposed to the single Navigation Module in the prior CCA5 architecture, as seen in Figure 1). (This largely holds for the CCA7 version of the architecture although in this version there are multiple Navigation Module B’s. However, in this section, one of the Navigation Module B’s can be considered.)

Similar to Navigation Module A, in Navigation Module B an instinctive primitive or a learned primitive (which themselves are navigation maps) in conjunction with the Working Navigation MapB **WNMB’** (similar to the Working Navigation Map **WNM’** that Navigation Module A operates on) may result in an action signal. This signal goes to the Output Vector Association Module B and to the external embodiment (Figure 5).

Working Navigation Map B **WNMB’** (107) is defined similarly to Working Navigation Map A **WNM’**–both are navigation maps, i.e., treated as arrays. Similarly, as equation (108) shows, any operation that in theory Navigation Module A can perform on a Working Navigation Map, Navigation Module B can also perform a similar operation on a similar Working Navigation Map. This implies that equations (1) to (106) above all also apply to the operations Navigation Module B on Working Navigation Map B **WNMB’**. Indeed, Navigation Module B originates from the duplication of Navigation Module A. However, the duplication is assumed to be somewhat of a partial duplication (not all pathways would have duplicated similarly necessarily) and in the architecture described in the main body of the text above, Navigation Module B is used mainly for compositional processing. Hence, in (108) there are what are called *navA_only_operation*’s—operations which are only performed by Navigation Module A.

While in the architecture described in the main body of the text above, Navigation Module B is used mainly for compositional language comprehension and resultant compositional behavior, in fact, Navigation Module B can be used for much more abstract compositional comparisons of two sets of data in the two Navigation Modules. This is beyond the scope of the present paper. The set of *navA_only_operation*’s can readily be adjusted in different versions of the architecture.

Consider a demonstration example shown above in Figure 6A. There are a number of shape objects in the sensory scene plus the instruction “place the black sphere on top of the black block which is not near a cylinder.” As described by equations (1) to (106) above, the objects in the sensory scene will become mapped/matched/updated onto the **WNM’** navigation map in Navigation Module A. The instruction, however, will trigger the instinctive primitive parse_sentence()(which is broken down in sub-parts in equations (109) to (111)).

Equation (109) shows that if a sensory input is recognized as an instruction sentence *instruction_sentence* then the instinctive primitive Nav_ModB.parse_sentence.copy()maps the instruction sentence onto Working Navigation Map B **WNMB’** in Navigation Module B.

The instinctive primitive Nav_ModB.parse_sentence.parse()then parses through the instruction sentence, i.e., Navigation Map B **WNMB’** in Navigation Module B (110). For each word in the instruction sentence Nav_ModB.parse_sentence.parse.match() matches the word against the Causal Memory Module (111). If an action word is found then it is mapped to cells in Navigation Map A **WNM’** in Navigation Module A which contain matching features to the cells in the instruction sentence (i.e., Navigation Map B **WNMB’** in Navigation Module B) associated with the action word.

Equation (112) shows that if a *near_trigger* occurs in parsing, then the instinctive primitive Nav_ModB.physics_near_object() is triggered. A *near_trigger* is a word (or other sensory input that does the same thing) related to the geometric concepts of near and not near. Continuing the compositional example, the word “near” acts as a *near_trigger* (Navigation Module B). Since there was a “not” beside it, and then “cylinder” this instinctive primitive flags the cell in Navigation Module A (i.e., Navigation Map A **WNM’**) with “cylinder” with the flag “not”. Also, the physics_near_object() instinctive primitive will mark “not” in the matched cell and all the adjoining non-null cells.

Equation (113) shows that once the instinctive primitive Nav_ModB.parse_sentence()has reached the end of the instruction or communication sentence, i.e., there is nothing left to parse, then it looks for cells which have been tagged or marked with tags. Possible associated tags depend on the collection of instinctive primitives, which at present only has the single option of place_object() (but thousands of instinctive and learned primitives are possible here).

Given a *<place>* tag in cell (0,0,0) of Navigation Module A in Figure 7B in the example in the text, the instinctive primitive Nav_ModA.place_object() will be triggered (113). Note that this instinctive primitive will operate on Navigation Module A (i.e., Navigation Map A **WNM’** which is the representation of the sensory scene.

The Nav_ModA.place_object()instinctive primitive will attempt to move and place an object somewhere. Continuing the example above, the tag <“place”> is most closely associated with “sphere, black”—that is the object that will be placed somewhere. The primitive now looks for other tagged notations such as <“top”> in the navigation map (Figure 7B). It will see a <“not”> in the cell with the features of a black block (Schneider, 2024) at (2,0,0) and not consider the <“top”> tag in that cell . However, the cell with the features of black block at (4,0,0) has a valid tag such as <“top”>. Thus, this primitive will in turn trigger the instinctive primitive Nav_ModA.move()to perform the movement of the black sphere to cell (4,0,0) (on top of it if three dimensions were being used) (114).

Nav_ModA.move() can be used for many actions of the CCA6. In this example, actions from Navigation Module A cause an action signal to go to the Output Vector Association Module A (Figure 5). The Output Vector Association Module A performs motion planning and motion corrections via feedback from the Sequential/Error Correcting Module. The corrected motion action signal from the Output Vector Association Module A is shown in Figure 5. This signal goes to the Output Vector Shaping Module which produces the actual signals (Figure 5) for actuators to move the black sphere to the cell with the black block on the right. Thus, the black sphere is then moved to (and on top of it in 3 dimensions) the black block on the right side.

Then a new cognitive cycle repeats again, finally processing again the actual sensory inputs streaming into the architecture.

**WNMB’** = ∈ R*^m^*^x^*^n^*^x^*^oxp^* (107)

∀ *operation_NavModA_*()**,**

( ~*navA_only_operation* ⟹ *operation_NavModB_*(**WNM***_m_*) = *operation_NavModA_*(**WNM***_m_*) ) (108)

( *instruction_sentence* )**,**

⇒**WNMB’***_t_* = Nav_ModB.parse_sentence.copy() (109)

⇒ Nav_ModB.parse_sentence.parse(**WNMB’***_t_*)**,** (110)

⇒ Nav_ModB.parse_sentence.parse.match() (111)

⇒ *near_trigger***,**

⇒Nav_ModB.physics_near_object() (112)

⇒ *end_of_communication***,**

*<place>,*

⇒ Nav_ModA.place_object() (113)

⇒ Nav_ModA.move() (114)

| **WNMB’** | -Working Navigation Map B  -the current Working Navigation Map B **WNMB’**_t_  is the navigation map which the Navigation Module B focuses its attention on, i.e., applies operations on to make a decision to take some sort or no action |
| --- | --- |
| ∀ *operation_NavModA_*(**WNM***_m_*)**,**  **(** ~*navA_only_operation* ⟹ *operation_NavModB_*(**WNM***_m_*) = *operation_NavModA_*(**WNM***_m_*) **)** | -any operation that in theory Navigation Module A can perform on some Working Navigation Map *m* in Navigation Module A **WNM***_m_* (*operation_NavModA_*(**WNM***_m_*)*)*, Navigation Module B can also perform a similar operation on a similar Working Navigation Map *m* **WNM***_m_* in Navigation Module B  -implies that equations (1) to (106) above all also apply to the operations of Navigation Module B on Working Navigation Map B **WNMB’**  **-***navA_only_operation*’s—operations which are only performed by Navigation Module A; set of these nav_modA-only operations can be adjusted for different versions of the architecture  -in CCA6 and CCA7 versions of the architecture, Navigation Module B largely used for compositional language comprehension and behavior |
| *instruction_sentence* | -a sensory input which triggers recognition as an input instruction sentence by the Input Sensory Vectors Association Module and/or Instinctive Primitives and/or Learned Primitives |
| **WNMB’***_t_* = Nav_ModB.  parse_sentence.copy() | -instinctive primitive parse_sentence.copy()maps the *instruction_sentence* instruction sentence onto Working Navigation Map B **WNMB’** in Navigation Module B |
| Nav_ModB.parse_sentence.  parse(**WNMB’***_t_*) | -instinctive primitive parse_sentence.parses()parses through the instruction sentence, i.e., Navigation Map B **WNMB’** in Navigation Module B |
| Nav_ModB.parse_sentence.  parse.match() | -for each word in the instruction sentence Nav_ModB.  parse_sentence.parse.match() matches the word against the Causal Memory Module  - if an action word is found then it is mapped to the Navigation Map A **WNM’** in Navigation Module A. |
| *near_trigger* | -word related to positional relationships of near and not near  -triggers instinctive primitive physics_near_object() |
| *near_trigger***,**  ⇒Nav_ModB.  physics_near_object() | -if a *near_trigger* occurs in parsing, then the instinctive primitive Nav_ModB.physics_near_object() is triggered  -the Nav_ModB.physics_near_object()will mark “near” (or “not” if “not” is specified in the instruction) the cell, in Navigation Map A **WNM’** in Navigation Module A, which matches to this instinctive primitive, as well as all the adjoining non-null cells. |
| *end_of_communication* | -end of a language sentence, i.e., nothing left to parse |
| *<place>* | -existence of a <”place”> tag |
| *end_of_communication***,**  *<place>,*  ⇒ Nav_ModA.  place_object() | -once the instinctive primitive Nav_ModB.parse_sentence()has reached the end of the instruction or communication sentence, i.e., there is nothing left to parse, then it will trigger associated tags  -possible associated tags depend on the collection of instinctive primitives, which at present only has the single option of place_object() (but thousands of instinctive and learned primitives are possible here)  -given a *<place>* tag in cell (0,0,0) of Navigation Module A in Figure 5 in the example in the text, the instinctive primitive Nav_ModA.place_object() is triggered  - Note that this instinctive primitive will operate on Navigation Module A (i.e., Navigation Map A **WNM’**)  - The Nav_ModA.place_object()instinctive primitive will attempt to move and place an object somewhere depending on what tags the other instinctive primitives Nav_ModB.parse_sentence.  parse.match()and Nav_ModB.physics_near_object()have written to Navigation Map A **WNM’** |
| Nav_ModA.move() | -once Nav_ModA.place_object()has a location to move the object to, Nav_ModA.move()is triggered and actually sends an action signal to the Output Vector Association Module A which then outputs a more processed action signal to other modules resulting in the architecture’s output actuators being instructed to move the object |

Table A26. Explanation of Symbols and Pseudocode in Equations (107) – (114)

| **Input:** | **WNM’***_t_* : the current Working Navigation Map A in Navigation Module A which is derived from the processed sensory inputs representing the sensory scene  **WNMB’***_t_* : the current Working Navigation Map B in Navigation Module B which is derived from a communication sentence  **WPR***_t_* : various Working Primitives **WPR**’s are the primitives that will be applied against the Working Navigation Map A **WNM’** in the Navigation Module A and/or Working Navigation Map B **WNMB’** in the Navigation Module B; these include:  - Nav_ModB.parse_sentence()  - Nav_ModB.physics_near_object()  - Nav_ModA.place_object()  - Nav_ModA.move() |
| --- | --- |
| **Output:** | *action_t_* : a signal to move some actuator or send an electronic signal  -it is sent from Navigation Module A to the Output Vector Association Module A (Figure 5) where more detailed instructions are created to effect the required actuator outputs to the Output Vector Shaping Module (1) to the actual real-world actuators |
| **Description:** | -A communication or instruction sentence is parsed in Navigation Module B and applied against a relevant represented sensory scene in Navigation Module A. As such, the beginnings of compositional language comprehension and behavior emerge.  - Applies to the CCA6 (Figure 5) and CCA7 (Figure 9) versions of the Causal Cognitive Architecture; however, there are multiple Navigation Module B’s in the CCA7 version, thus one of the Navigation Module B’s can be considered |

Table A27. Summary of the Operation of Compositional Language Comprehension per Equations (107) – (114)

A.14 Enhanced Navigation Module B’s

This section only applies to the CCA7 version of the architecture.

Consider an agent, i.e., a robot, controlled by the CCA7 architecture shown in Figure 9. For simplicity the CCA7 architecture and the robot embodiment will be called the “CCA7” or “CCA7 robot.” The CCA7 robot comes to location “X” in Figure 10. It receives the instruction that starting at its existing position (i.e., “X”) it must visit each object and then return to the starting location.

While in location “X” the CCA7 robot maps a sensory scene into the navigation map in Navigation Module A, which is what it automatically does in each available cognitive cycle when there are new sensory inputs to process. The resulting navigation map in Navigation Module A is shown in Figure 11A. The CCA7 robot receives distances (either with the visual sensory information or via a separate ultrasonic distance sensory system). The numbers refer to the distance (in centimeters) between the objects in the different cells. (The distance number can be determined by matching the same number in the path between two cells. As well, note a clockwork recording of distances in each cell.) The instruction “go to all objects and go back” is placed in Navigation Module B as shown in Figure 11B. These operations are similar in nature to ones already described above for the CCA6 version of the architecture in its initial processing of the example of the sensory scene and instruction concerning the “placing a black sphere on top of the black block which is not near a cylinder” (Figure 6, Figure 7).

However, as described above and shown in Figure 9, there are now in the CCA7 multiple Navigation Modules—one Navigation Module A and over a thousand (1024) duplicated Navigation Module B’s. Equation (115) (taken from Appendix A in the Supplementary Material section) indicates that the Working Navigation Map B’ **WNMB’** (upon which primitives operate in Navigation Module B) is an array like before, but now can be one of 1024 different navigation maps (corresponding to a different navigation map in each of the Navigation Module B’s.)

The Navigation Module B’s are numbered n=1 to n=1023. The top (or first) Navigation Module B appears to be the n=1 Navigation Module B, as shown in Figure 9. However, a n=0 Navigation Module B exists and is used to store a copy of the compositional instructions so that if the other layers are overwritten, there is still a copy of the instructions. Layer n=0 is considered “reserved” and will not be overwritten. If there is other information that an instinctive or learned primitive needs to ensure remains intact for the current operations, other Navigation Module B’s can be temporarily designated “reserved” as well.

Equation (116) indicates that the same instinctive primitive or the same learned primitive is initially applied to all of the Navigation Module B’s. (In subsequent cognitive cycles the initial primitive applied may trigger different primitives in different Navigation Modules.) If an instinctive primitive or learned primitive does not examine processing paths in parallel, then it will simply use the results of operations in Navigation Module B n=1 and ignore the other Navigation Module B’s.

As shown in Figure 11A is Navigation Module A containing Working Navigation Map (**WNMA**) of the sensory scene of the various places the agent has to navigate to. In Navigation Module B n=1 (Figure 11B) is a Working Navigation Map (**WNMB**_n=1_) of the instruction sentence to “go to all the objects and go back.”

The word “go” in the first cell of the navigation map in Navigation Module B (Figure 11A) is matched against the Causal Memory Module as an action word and triggers the instinctive primitive “goto()” (117). “**WNMA’***_t_* = Nav_ModA.goto()” indicates that this instinctive primitive, i.e., “goto()” is being applied to the Working Navigation Map A in Navigation Module A.

The instinctive primitive “goto()” causes the CCA7 robot to tag a location(s) and then essentially move to whatever location is indicated by the tag(s). The word “all” which is associated with active word <“go”> (until another action word is encountered, as in an earlier example above) will cause the tag <“all”> to be placed in all the cells with objects in the navigation map in Navigation Module A (Figure 12A).

The words “go back” is also associated with the instruction word <“go”> and will cause the tag <“back”> to be placed in the starting cell (which is (0,0,0) in this example). This can be seen in Figure 12A.

Once the instinctive primitive “goto()” tags the cell(s) where it has to move to, it then decides if it will move (i.e., “go to”) the cell with the tag. However, if there are multiple tags, i.e., multiple locations to navigate to (“locations > 1” in (118–123)), then the “small_plan()” instinctive primitive is activated instead of moving to a single location. As discussed above, this instinctive primitive will plan a navigation route to whatever multiple tagged locations are indicated on the navigation map(s).

Once activated (118), the instinctive primitive “small_plan()” (regardless of argument) will copy the Navigation Map A to all “non-reserved” Navigation Module B’s, i.e., n=1…1023 in this example. It will remove any action words such as “go” in the example above. This copying is indicated by the arrow in Figure 12. Thus, the instruction sentence in Navigation Module B n=1 (Figure 11) is overwritten here. (Navigation Module B n=0 is “reserved” for a copy of the instruction sentence, although it actually will not be used again in this example.) A number of existing operations in various instinctive primitives already transfer or compare the contents of Navigation Module A and Navigation Module B with each other. Thus, the emergence of this step is a feasible one in the continued evolution of the architecture.

Equation (118) describes instinctive primitive “small_plan(random=False)” acting on the Working Navigation Map (**WNMB’***_t,_* _n=1_) in Navigation Module B n=1 (Nav_ModB_n=1_) at time t=t (i.e., at some specific time). The instinctive primitive “small_plan(random=False)” follows nearest neighbour algorithm discussed above. In making a plan where to navigate to, this primitive will choose the tag (i.e., location) that is closest to tag (i.e., location) from where it is navigating. The argument random=False indicates that this instinctive primitive does not introduce any random variations. As will be seen below, in the other Navigation Module B’s n=2…1023 random variations will be introduced.

In n=1 Navigation Module B, “small_plan(random=False)” operates on the navigation map shown in Figure 12b and decides which tagged cell to navigate first. It uses a nearest neighbor algorithm in its planning actions. For example, in Figure 12B, the cell (0,0,0) in which the CCA7 is starting from, has a distance of 25, 31, and 42 units (actually centimeters, but “small_plan()” will disregard the actual units) to the other objects. (They are listed as “25, 42, 31” in cell (0,0,0) in Figure 12B due to a clockwise organization of distances.) According to the nearest neighbor algorithm it chooses the shortest distance, which is 25, i.e., it plans to navigate first to cell (2,2,0) containing the white sphere. Thus, it changes the <“all”> tag to a <1>. This can be seen in Figure 12C.

The instinctive primitive then considers navigating from cell (2,2,0)—which object to navigate to next? As can be seen in Figure 12B or Figure 12C, 22 is the shortest distance, thus it decides to navigate to cell (3,0,0) which contains the black sphere. It changes the <“all”> tag to a <2>. It then considers navigating from cell (3,0,0)—which object to navigate to next? Actually, the only untagged object remaining is the white block in cell (4,2,0), which is then tagged with a <3>. This is shown in Figure 12C.

If this was the previous CCA6 version of the architecture (albeit, retrofitted with these new equations) with only one Navigation Module B, then at this point the instinctive primitive “small_plan(random=False)” would trigger the instinctive primitive “move()” to move a CCA6 robot to cell (2,2,0) containing the white sphere. Then the instinctive primitive “move()” is triggered again to move to cell (3,0,0) containing the black sphere. Then the instinctive primitive “move()” is triggered again to move to cell (4,2,0) containing the white block. Then the instinctive primitive “move()” is triggered again to move to cell (0,0,0) which was the starting point. From Figure 12C, note that the sum of the distances is 25+22+20+42= 109 cm in this navigation route.

However, in the CCA7 version of the architecture being considered here, there are over a thousand Navigation Module B’s. As equation (120) indicates, for Navigation Module B n=2…1023 the instinctive primitive “small_plan(random=weight_distance)” will perform a similar nearest neighborhood planning algorithm in the other modules for this same navigation map (Figure 12B). However, as indicated by the argument random=weight_distance random fluctuations are introduced now, so a slightly different navigation route may occur in different Navigation Module B’s n=2…1023 (14D).

The instinctive primitive “small_plan(random=weight_distance)” follows a similar nearest neighbor algorithm to the one described above. However, now random fluctuations may (or may not) be introduced at each step a navigation decision is made. These fluctuations are weighted by distance position, as explained below. Normally, the destination with the shortest distance will be chosen, as seen above for Navigation Module B n=1 (Figure 12C). Here this is likely to occur also, but some randomness means another destination can be chosen, although the destinations the farthest away are the least likely to be chosen as the next destination, as will be shown below.

Consider that there is at any given decision point the list ***destination*** containing sorted destinations [a ,b, c, d, e…] which still can be navigated to (124). This list is sorted by distance such that navigation to destination “a” is the shortest, then navigation to destination “b” is the next shortest, and so on (125). The value a in the list is the distance to destination “a”, the value b in the list the distance to destination “b”, and so on.

Consider an example where there are 5 possible destinations which the CCA7 can now navigate to from some starting point, i.e., to object “a”, to object “b”, to object “c”, to object “d” or to object “e”. As per (124) ***destination*** = [a, b, c, d, e] , where the distance from the starting point to “a” is less than or equal to the distance from the starting point to “b”, and so on (125). Object “a” represented by element a in ***destination*** is considered to have position=1 in the list, while object “b” has position=2, and so on. Similarly, object “a” is considered to have inverse_position=5 in this list, while object “b” has inverse_position=4, and so on (124).

Equation (126) shows that when the instinctive primitive “small_plan(random= weight_distance)” is triggered, a parameter “*weight*” is given a value of 4. Equation (127) shows that when the instinctive primitive “small_plan(random= weight_distance)” is triggered, the probability of selecting destination “x” to navigate to is given by “probability_destination_x_” which can be computed as “inverse_position_x_^*^weight^* / ∑ inverse_position^*^weight^* ”.

Continuing with the example above of choosing to navigation to locations “a”, “b”, “c”, “d” or “e”, consider equations (126) and (127). Consider navigating to the first destination “a” (which is the shortest navigation path from the starting point since it is the first element in ***destination***). Thus, as per (127), x = “a” and the value of the term “inverse_position_a_^*^weight^* ” is thus 5^ *^weight^*. The parameter *weight*=4 (126), thus the value of the term “inverse_position_a_^*^weight^* ” is 5^4, or 625. Similarly, the value of all the inverse positions raised to the fourth power (*weight*=4) added up, i.e., “∑inverse_position^^^*^weight^* ” is 625+256+81+16+1=979 (127).

(In the actual CCA7 version of the architecture, other than as needed internally (and encapsulated) for artificial neural networks being used, only very simple arithmetic is explicitly available. Thus, in (127) the “probability_destination_x_” is shown as being approximately equal to a term which must be calculated via high exponential powers and involves the manipulation of many decimal places. While (127) is fine for some simulations of the architecture, the relationship shown in (127) can be achieved more realistically by the architecture by making use of stored probability distributions (see below). A limited number of such probability distributions can approximate (127) in deciding which object or city to navigate next to in a planning task.)

Continuing with the example above of choosing to navigation to locations “a”, “b”, “c”, “d” or “e”, the instinctive primitive “small_plan(random= weight_distance)” has just been triggered. Thus, *weight* is given a value of 4 (126). The probability of the algorithm in this instinctive primitive choosing, for example, destination “a” to navigate next to, is probability_destination_a_. By (127) this is equal to “= inverse_position_a_^*^weight^* / ∑ inverse_position^*^weight^*”. Above we calculated term “inverse_position_a_^*^weight^* ” to be 625, and the term “∑inverse_position^^^*^weight^* ” to be 979. Thus, the probability of the algorithm in this instinctive primitive to choose destination “a” to navigate next to is 625/979, or 64%.

From similar calculations the probability of choosing any of these sorted destinations (i.e., “a” is closer, “e” is the farthest away from the starting point) in this example of [a, b, c, d, e] is [64%, 26%, 8%, 2%, .1%]. Thus, when small_plan(random=weight_distance)is used in this example, of the five potential destinations to choose from in [a, b, c, d, e], there is in this example, a 64% chance of navigating to the nearest neighbor “a”, a 26% chance of navigating to the next nearest neighbor “b”, but only a 0.1% chance of navigating to the farthest neighbor “e”.

In (128) it can be seen that when the instinctive primitive “small_plan(random= False)” is triggered, a parameter “*weight*” is given a value of 30. The result of this high *weight* is that is that the nearest neighbor destination is always used, i.e., there is little randomness (129). Thus, the probability distribution for navigation to potential objects/cities [a, b, c, d, e] is [100%, 0, 0, 0, 0], i.e., there is a 100% chance of choosing object/location “a’ to navigate to, and 0% chance of choosing object/location “b”, “c”, “d” or “e” to navigate to.

As noted above, if there is only one Navigation Module B in the system, or if this is a CCA7 version of the architecture and this is Navigation Module B n=1, then as equation (118) indicates, the instinctive primitive “small_plan(random=False)” is triggered. The nearest neighbor (i.e., shortest distance) from the starting point of cell (0,0,0) (Figure 12C; there is a <0> put in that cell) is cell (2,2,0)—there is a <1> put tag in that cell. The next nearest neighbor is cell (3,0,0)—there is a <2> put tag in that cell. The next nearest neighbor is the only one left which is cell (4,2,0)—there is a <3> tag put in that cell. Then with no more active cells to navigate to left, there is navigation back to the starting point of (0,0,0)—there is a <0> tag there.

Once all cells are tagged, the instinctive primitive “small_plan(random=False)” would trigger the instinctive primitive “move()” to move a CCA7 robot to the tagged cells (122, 123). The instinctive primitive “move()” first moves the CCA7 to cell (2,2,0) with the tag <1> containing the white sphere. Then the instinctive primitive “move()” is triggered again to move to cell (3,0,0) with the tag <2> containing the black sphere. Then the instinctive primitive “move()” is triggered again to move to cell (4,2,0) with the tag <3> containing the white block. Then the instinctive primitive “move()” is triggered again to move to cell (0,0,0) containing the tag <0> which was the starting point. From Figure 10 and Figure 12C, note that the sum of the distances is 25+22+20+42= 109 cm in this navigation route.

Now consider the Navigation Module B’s n=2…1023 in the CCA7 version of the architecture. In Figure 12D Navigation Module B n=2 is shown. As equation (120) indicates, the instinctive primitive “small_plan(random=False)” is triggered. As before the instinctive primitive “small_plan()” considers which possible destination it can navigate to is the shortest, albeit now with a random fluctuation introduced. From the starting point of cell (0,0,0) the CCA7 can navigate next to cells (2,2,0), (3,0,0) or (4,2,0). From Figure 10 it can be seen these correspond to possible distances of (25cm, 31cm and 42cm).

As discussed above, “small_plan(random=weight_distance)” will introduce a random fluctuation in deciding which object/location to navigate to via equations (124–127). The sorted list [25, 31, 42] (124, 125). The sum of the inverse positions is 3^4+2^4+1^4, or 98, and thus the probability distribution is [81/98,16/98,1/98], or [83%, 16%, 1%]. The likelihood of navigating to the first position destination of 25cm corresponding to cell (2,2,0) is 83%, while the probability of navigating to (3,0,0) is 16% and the probability of navigating to (4,2,0) is 1%. A cumulative probability distribution results essentially from considering these probabilities: [=<83%, =<99%, =<100%]. A random number between 0 and 1 is obtained which happens to be, for example, .55 or 55%. It is within the 83% cumulative probability corresponding to the first position destination. Thus the CCA7 architecture tags cell (2,2,0) with a <1>.

Now the instinctive primitive “small_plan(random=weight_distance)” must consider navigating to the next object/location. From the starting point of cell (2,2,0) the CCA7 can navigate next to cells (3,0,0) or (4,2,0). From Figure 10 it can be seen that these correspond to possible distances of (22cm and 24cm). The sum of the inverse positions is 2^4+1^4, or 17, and thus the probability distribution is [16/17,1/17], or [94%, 6%]. The likelihood of navigating to the first position destination of 22cm corresponding to cell (3,0,0) is 94%, while the probability of navigating to (4,2,0) is 6%. A cumulative probability distribution results essentially from considering these probabilities: [=<94%, =<100%]. A random number between 0 and 1 is obtained which happens to be, in this case, for example, .95 or 95%. Thus, instead of navigating to the nearest neighbor, the CCA7 will tag the second nearest neighbor, i.e., (4,2,0) as the next destination to navigate to with a <2> (Figure 12D). The next nearest neighbor is the only one left which is cell (3,0,0)—there is a <3> tag put in that cell. Then with no more active cells to navigate to left, there is navigation back to the starting point of (0,0,0)—there is a <0> tag there. The tagged cells can be seen in Figure 12D.

From Figure 10 and Figure 12D note that the sum of the distances is 25+24+20+31= 100 cm in this navigation route. Thus, even though this route ended up taking a path between two locations which was not the shortest distance (i.e., going from cell (2,2,0) to cell (4,2,0) which was 24cm rather than going to cell (3,0,0) which was 22cm) it turned out that the total distance in navigating to all object/locations turned out to be shorter than the path obtained in Navigation Module B n=1 where the nearest neighbor algorithm was followed at each decision point.

Similar algorithms are also running in the other Navigation Module B’s at the same time. The total distance sum obtained in each Navigation Module B is transferred to the TempMap memory areas of Navigation Module B n=0 (119, 121). Although there are many more **TempMap** memory areas now available in the CCA7 version of the architecture, this instinctive primitive actually just keeps track of the navigation map number (i.e., which “n” from n=1…1023) which has yielded the smallest total distance of the best (i.e., shortest) navigation plan found (122). Thus **Nav_ModB**_n=best_  where “best” is the Navigation Module B “n” which showed the shortest total navigation distance.

The instinctive primitive “small_plan()” then activates the instinctive primitive move(**WNMB’***_t,_* _n=best_ ) (123). To continue the above example, “best” is Navigation Module B n=2, i.e., shown in Figure 12D. (The total distance here was 100cm versus 109cm in Navigation Module B n=1, and versus 119cm obtained in other Navigation Module B’s). It will then repeatedly trigger the instinctive primitive “move()” to navigate to <1> (the white sphere), then <2> (the white block), then <3> (the black sphere), and then return to the starting cell <0>.

This navigation planning example involves navigating to three locations and then returning back to the starting position. Thus, there are only a handful of possible variations in navigation to consider, and thus, despite the random fluctuations, many variations will repeat among the over thousand Navigation Module B’s. However, many real-world problems may involve more locations (or social situations or other analogous “locations”) than this simple problem. In the next section, the CCA7 architecture will be applied to a larger dataset of navigation locations.

The instinctive primitive “small_plan()” effectively helps to decide what sequence to perform operations in. While such problems can be physically moving to different locations, they can also range from navigating in the social hierarchy space of a society to involve navigating through an idea space of more abstract concepts.

**WNMB’**_n=0…1023_  = ∈ R*^m^*^x^*^n^*^x^*^oxp^* (115)

(**WNMB’** _n=x_ ≠ reserved AND **WNMB’** _n=y_ ≠ reserved )
 ⇒ *initial_primitive***_WNMB’_**_n=x_  = *initial_primitive***_WNMB’_**_n=y_  (116)

<“go”>

⟹ **WNMA’***_t_* = Nav_ModA.goto() (117)

locations > 1**,**

⟹ **WNMB’***_t,_* _n=1_ = Nav_ModB_n=1_.small_plan(random=False) (118)

⟹ **TempMap_WNMB’_** _n=0_ = minimum(total_distance_n=1_) (119)

⟹ **WNMB’***_t,_* _n=2…1023_

= Nav_ModB *_t,_*  _n=2…1023_.small_plan(random=weight_distance) (120)

⟹ **TempMap_WNMB’_** _n=0_ = minimum(total_distance_n=2…1023_) (121)

⟹ best = **TempMap_WNMB’_** _n=0, minimum(total_distance)_ (122)

⟹ Nav_ModB_n=best_.move(**WNMB’***_t,_* _n=best_ ) (123)

***destination*** = [a, b, c, d, e…] (124)

distance_a_ <= distance_b_ , distance_b_ <= distance_c_ , …. (125)

small_plan(random=weight_distance)**,**

⟹ *weight* = 4 (126)

⟹ probability_destination_x_ ≈ inverse_position_x_^*^weight^* / ∑ inverse_position^*^weight^* (127)

small_plan(random=False)**,**

⟹ *weight* = 30 (128)

⟹ probability_destination_a, weight>9_ = 1 (129)

| **WNMB’**_n=0...1023_ | -Working Navigation Map B n=0 to n=1023  -there are now 1024 Navigation Module B’s, and each one has a separate Working Navigation Map B **WNMB’**_n_ |
| --- | --- |
| **WNMB’**_n=0…1023_  = ∈ R*^m^*^x^*^n^*^x^*^oxp^* (115) | -the current Working Navigation Map B **WNMB’**_n,t_ is the navigation map which the Navigation Module B_n_ focuses its attention on, i.e., applies operations on to make a decision to take some sort or no action - Navigation Module B_n=0_  to Navigation Module B_n=1023_  all operate in parallel - Navigation Module B_n=0_ is “reserved” for a copy of the compositional instructions, thus operations really occur in Navigation Module B’s n=1 to n=1023 |
| (**WNMB’**_x_ ≠ reserved AND  **WNMB’**_y_ ≠ reserved ) ⇒ *initial_primitive***_WNMB’_**_n=x_  = *initial_primitive***_WNMB’_**_n=y_  (116) | -the same initial instinctive primitive or learned primitive is applied to all the Working Navigation Maps in the different Navigation Module B’s (unless a Navigation Module B is reserved in which case any primitives will be ignored in that particular reserved Navigation Module B) |
| <“go”> ⇒  **WNMA’***_t_* = Nav_ModA.goto() (117) | -if an instruction related to go to a place or location occurs, then the instinctive primitive goto()will be triggered  -instinctive primitive Nav_ModA.goto()will tag in the navigation map in the Navigation Module A the cells where the CCA7 or an object is supposed to go to  - once the instinctive primitive “goto()” tags the cell(s) where it has to move to, it then decides if it will move (i.e., “go to”) the cell with the tag; however, if there are multiple tags, i.e., multiple locations to navigate to (“locations > 1” in (118–123)), then the “small_plan()” instinctive primitive is activated instead of moving to a single location |
| locations > 1**,**  ⟹ **WNMB’***_t,_* _n=1_ = Nav_ModB_n=1_.small_plan(random=False) (118)  ⟹ **TempMap_WNMB’_** _n=0_ = minimum(total_distance_n=1_) (119) ⟹ best =  **TempMap_WNMB’_** _n=0, minimum(total_distance)_ (122) | - once activated (118), the instinctive primitive “small_plan()” (regardless of argument) will copy the Navigation Map A to all “non-reserved” Navigation Module B’s, i.e., n=1…1023 generally  -it will remove any action words (e.g., “go” in the example above.)  - the instinctive primitive “small_plan(random=False)” follows nearest neighbour algorithm discussed above; in making a plan where to navigate to, this primitive will choose the tag (i.e., location) that is closest to tag (i.e., location) from where it is navigating.  -the argument random=False indicates that this instinctive primitive does not introduce any random variations  - instinctive primitive “small_plan()” operates on all the Navigation Module B’s in parallel; the total distance sum obtained in each Navigation Module B is transferred to the TempMap memory areas of Navigation Module B n=0 (119, 121); although there are many more **TempMap** memory areas now available in the CCA7 version of the architecture, this instinctive primitive actually just keeps track of the navigation map number (i.e., which “n” from n=1…1023) which has yielded the smallest total distance of the best (i.e., shortest) navigation plan found (122) |
| ⟹ **WNMB’***_t,_* _n=2…1023_  = Nav_ModB *_t,_*  _n=2…1023_.small_plan(random=weight_distance) (120)  ⟹ **TempMap_WNMB’_** _n=0_ = minimum(total_distance_n=2…1023_) (121)  ⟹ best =  **TempMap_WNMB’_** _n=0, minimum(total_distance)_ (122) | - just like (118), as (120) indicates, for Navigation Module B n=2…1023 the instinctive primitive “small_plan(random=weight_distance)” will perform a similar nearest neighborhood planning algorithm in the other modules for this same navigation map (Figure 12b); however, as indicated by the argument random=weight_distance random fluctuations are introduced now, so a slightly different navigation route may occur in different Navigation Module B’s n=2…1023  - “small_plan(random=weight_distance)” follows a similar nearest neighbor algorithm to the one shown above. However, random fluctuations are introduced at each step a navigation decision is made – see below  - the destination with the shortest distance will most likely be chosen, as seen above for Navigation Module B n=1; but some randomness means another destination can be chosen, albeit the destinations the most far away, are the least likely to be chosen as the next destination  - instinctive primitive “small_plan()” operates on all the Navigation Module B’s in parallel; the total distance sum obtained in each Navigation Module B is transferred to the TempMap memory areas of Navigation Module B n=0 (119, 121); although there are many more **TempMap** memory areas now available in the CCA7 version of the architecture, this instinctive primitive actually just keeps track of the navigation map number (i.e., which “n” from n=1…1023) which has yielded the smallest total distance of the best (i.e., shortest) navigation plan found (122) |
| destination = [a, b, c, d, e…] (124)  distance_a_ <= distance_b_ , distance_b_ <= distance_c_ , …. (125) | - consider that there are at any given decision point the sorted destinations [a ,b, c, d, e…] which still can be navigated to (124). This list is sorted by distance such that navigation to destination a is the shortest, navigation (i.e., again from the starting point) to destination b is the next shortest, and so on (125). The value a in the list is the distance to destination a, the value b in the list the distance to destination b, and so on.  - |
| small_plan(random=weight_distance)**,**  ⟹ *weight* = 4 (126)  ⟹ probability_destination_x_ ≈ inverse_position_x_^*^weight^* / ∑ inverse_position^*^weight^* (127) | - The index value of a in [a, b, c, d, e] is 0. However, the “position” of a is considered here to be 1.The positions of these destinations a, b, c ,d, e,... can be considered to be 1,2,3,4,5... Consider an example where there are 5 possible destinations in total: [a, b, c, d, e]. The inversion positions of these destinations in this example, can be considered to be 5, 4, 3, 2,1. Thus, the value of the inversion position for destination “a” would be 5. Continuing with this example and looking at equation (127), where destination x = “a”, the value of the term “inverse_position_a_^*^weight^* ” is thus 5^ *^weight^*, or since *weight*=4 (126), 5^^4^, or 625. Similarly, the value of all the inverse positions raised to the fourth power (*weight*=4) added up, i.e., “∑inverse_position^^^*^weight^* ” is 625+256+81+16+1=979 (127).  - Equation (126) shows that when the instinctive primitive “small_plan(random= weight_distance)” is triggered, a parameter “*weight*” is given a value of 2. Equation (127) shows that when the instinctive primitive “small_plan(random= weight_distance)” is triggered, the probability of selecting destination “x” to navigate to is given by “probability_destination_x_” which can be computed as “inverse_position_x_^*^weight^* / ∑ inverse_position^*^weight^* ”.  - Consider the example just mentioned where there are five potential destination locations which can be navigated to [a, b, c, d, e]. The instinctive primitive “small_plan(random= weight_distance)” has just been triggered. Thus, *weight* is given a value of 4 (126). The probability of the algorithm in this instinctive primitive choosing, for example, destination “a” to navigate next to, is probability_destination_a_. By (127) this is approximately equal to “inverse_position_a_^*^weight^* / ∑ inverse_position^*^weight^*”. Above we calculated term “inverse_position_a_^*^weight^* ” to be 625, and the term “∑inverse_position^^^*^weight^* ” to be 979. Thus, the probability of the algorithm in this instinctive primitive to choose destination “a” to navigate next to is 625/9795, or 64%.  -note: the exponential arithmetic of (127) is not actually performed by the CCA7 but instead the same result is obtained via the use of pre-stored probability distributions for different arrangements of destination locations |
| small_plan(random=False)**,**  ⟹ *weight* = 30 (128)  ⟹ probability_destination_a, weight>9_ = 1 (129) | -when the instinctive primitive “small_plan(random= False)” is triggered, a parameter “*weight*” is given a value of 30 -the result of this high *weight* is that is that the nearest neighbor destination is always used, i.e., there is little randomness; the probability distribution for navigation to potential objects/cities [a, b, c, d, e] is [100%, 0, 0, 0, 0], i.e., there is a 100% chance of choosing object/location “a’ to navigate to, and 0% chance of choosing object/location “b”, “c”, “d” or “e” to navigate to |
| ⟹ best = **TempMap_WNMB’_** _n=0, minimum(total_distance)_ (122)  ⟹Nav_ModB_n=best_.move  (**WNMB’***_t,_* _n=best_ ) (123) | - the total distance sum obtained in each Navigation Module B is transferred to the TempMap memory areas of Navigation Module B n=0 (119, 121); this instinctive primitive actually just keeps track of the navigation map number which has yielded the smallest total distance of the best (i.e., shortest) navigation plan found (122)  - **Nav_ModB**_n=best_  where “best” is the Navigation Module B “n” which showed the shortest total navigation distance.  - the instinctive primitive “small_plan()” then activates the instinctive primitive move(**WNMB’***_t,_* _n=best_ ) (123).  - it will then repeatedly trigger the instinctive primitive “move()” to navigate to the tagged locations in order |

Table A28. Explanation of Symbols and Pseudocode in Equations (115) – (129)

| **Input:** | **WNM’***_t_* : the current Working Navigation Map A in Navigation Module A which is derived from the processed sensory inputs representing the sensory scene  **WNMB’***_t_* : the current Working Navigation Map B in Navigation Module B n=1 which is derived from a communication sentence (a copy is also present in Navigation Module B n=0)  **WPR***_t_* : various Working Primitives **WPR**’s are the primitives that will be applied against the Working Navigation Map A **WNM’** in the Navigation Module A and/or Working Navigation Map B **WNMB’** in the Navigation Module B; these include:  - Nav_ModB.goto() - Nav_ModB.small_plan() - small_plan.random()(used internally by small_plan()) - Nav_ModB.minimum())(shown for (119,121) but used internally by other primitives also)  - Nav_ModB.move()  -Note: No advanced arithmetic is performed by any of these instinctive primitives. There is no requirement for the use of other instinctive or learned primitives for exponential arithmetic operations or complex decimal operations, as the exponential arithmetic of (127) is not actually performed by the CCA7 but instead the same result is obtained via the use of pre-stored probability distributions for different arrangements of destination locations |
| --- | --- |
| **Output:** | *action_t :_* a signal to move some actuator or send an electronic signal  -where the CCA7 robot is physically moving or an object is being moved, the signal *action_t_* is sent from Navigation Module B n=best to the Output Vector Association Module B where more detailed instructions are created to effect the required actuator outputs to the Output Vector Shaping Module to the actual real-world actuators |
| **Description:** | -A communication or instruction sentence is parsed in Navigation Module B and applied against a relevant represented sensory scene in Navigation Module A.  -If the communication concerns navigating to more than one location then the instinctive primitive small_plan()is triggered and used to plan an optimal-like navigation route to all the specified locations, such that actual planning emerges.  -applies to the CCA7 version of the architecture (can be retrofitted to the CCA6 version but not done at the time of writing) |

Table A29. Summary of Navigation Planning per Equations (115) – (129)

# Appendix B: Experimental Data

B.1 Methods

The equations (1) to (129) (Supplementary Material, Appendix A) are computer simulated via the Python language to represent the CCA7. The computer simulation does not interface with a real-time actual video camera or microphone inputs, or with real robotic actuators. Sensory inputs are simulated in all simulations, and actuator outputs are similarly simulated.

The navigation maps in the Python simulation have 6x6x0 dimensions (although internally a larger number of dimensions are actually used to represent segmentation of objects and binding with motion and action). As noted above navigation maps are essentially arrays. Thus, the more efficient Numpy library is called by the Python program for most operations on the navigation maps. For future larger simulations of the architecture, more classical deep learning software and hardware can be used. However, in the current simulation the FuzzyWuzzy string matching library (via pypi.org) is used for pattern matching.

The Python simulation of the architecture at this time contains a very limited set of instinctive primitives. It mainly contains the ones specified in equations (1) to (129) which relate to very basic operations and the ability for causal reasoning, analogical induction, compositionality and as discussed above in the section on new work, simple planning. At this time, instinctive primitives must be hand crafted. Automated methods for instinctive primitive creation are being explored.

Python version 3.11 is used. The parallel elements of equations (1) to (129) are simulated sequentially—a new cognitive cycle starts when all the operations of the previous cognitive cycle have completed. At present, due to the limited number of instinctive primitives and the limited storage of navigation maps representing experiences, this operation gives satisfactory run times on typical laptop or desktop personal computers (circa 2023).

The main purpose of this computer simulation is to show that the operation of the CCA7 version of the architecture is feasible, particularly its ability to perform planning operations. The simulation, i.e., based on the representation of the CCA7 version of the architecture via equations (1) to (129), can be tested below on a traveling salesperson dataset. The distances between a starting city and a dozen other cities are given in Table B1 (Google OR-Tools, 2023).

The reason for using the positional weighting in the equations (124–127) above is because the actual CCA7 version of the architecture only explicitly has access to very simple arithmetic. Thus, in weighting the probability distributions it was desired to use pre-stored distributions which could readily be accessed rather than involve complex calculations. Although (124–127) are used in the Python simulation of the architecture (albeit, necessitating the Python “Decimal” class due to the many digits created by the high-power exponents), the architecture can simply access a limited number of probability distributions based on the positions of the nearest city/location/object in a list, with no complex arithmetical calculations required.

It is possible, of course, to weight by the relative values: calculate the reciprocals of the difference of each number (nominal value of 1) in the list from the smallest value (thus, smaller difference will give a larger reciprocal) and normalize as probabilities. For example, if there are three possible locations to navigate to with distances (arbitrary units not specified) of [12, 13, 44] then the weight probabilities would be normalize([1/1, 1/1, 1/32]), or [49%, 49%, 2%]. Thus, there would be a 49% chance of navigating to the location 12 units away versus a 2% chance of navigating to the location 44 units away. The probability distribution for the example above of three possible locations [12, 42, 44] is normalize([1/1, 1/30, 1/32]), or [94%, 3.1%, 2.9%]. In contrast, the positional probability distribution (i.e., via (124–127)) of either example yields [83%, 16%, 1%], i.e., 83% chance of navigating to the first-closest location, 16% chance of navigating to the second-closest location and a 1% chance of navigating to the third-closest location, regardless of the actual distances.

Computer simulations of the CCA7 architecture can be tested on a traveling salesperson dataset. The distances between a starting city and a dozen other cities are given in Table B1 (Google OR-Tools, 2023). This table has the distances between the following cities: 0. New York - 1. Los Angeles - 2. Chicago - 3. Minneapolis - 4. Denver - 5. Dallas - 6. Seattle - 7. Boston - 8. San Francisco - 9. St. Louis - 10. Houston - 11. Phoenix - 12. Salt Lake City

The optimal (i.e., shortest) solution obtained via brute force computation is 7293 miles. (The data in Table B1 of distances between the cities was given in miles.) The route giving this shortest path is: City #0,7,2,3,4,12,6,8,1,11,10,5,9,0 .

The traveling salesperson problem city data from Table B1 was simulated as sensory input data to the CCA7 architectures. The same CCA7 version of the architecture shown in Figure 9 was used. However, equations (126–129) were modified in different runs of the architecture as described below. As described above, the CCA7 architecture via the instinctive primitives associated with equations (126–129) attempts to produce the shortest path in a planning problem. In the case of the city data from Table B1, the architecture attempts to produce the shortest path to navigate once to the dozen cities listed in Table B1 and return to the starting city (i.e., 13 cities in total).

The following questions were asked, and the accompanying comparative experiments were then performed:

**a.** The position-weighted algorithm used to inject random fluctuations into the nearest neighbor algorithm (126–129) uses a *weight* parameter to create a probability distribution used in selecting the next destination to navigate to. At present a *weight* value of 4 is used.

What is the effect of varying the *weight* parameter on the shortest path yielded in the traveling salesperson problem, i.e., is the value of *weight* used in equation a reasonable one, based on a typical planning problem represented by the data in Table B1?

**b.** Do the multiple Navigation Module B’s allow better planning in terms of this traveling salesperson problem represented by the data in Table B1?

Multiple runs to ensure statistical significance (or insignificance) of the following is to be examined: the shortest distance obtained by a CCA7 architecture modified to use only 1 Navigation Module B versus a CCA7 architecture using 1023 Navigation Module B’s.

Note: When multiple Navigation Module B’s are used, Navigation Module B n=0 is restricted for holding a copy of any instructions, hence, 1K-1 results in 1023 Navigation Module B’s available.

Note: As per equation (118) Navigation Module B n=1 uses the instinctive primitive small_plan(random=False)thus *weight* is set to 30 for this case, i.e., nearest-neighbor algorithm without any random fluctuations. However, the Navigation Module B’s n=2…1023 per equation (120) use the instinctive primitive small_plan(random=weight_distance)thus *weight* is set to 4 for these Navigation Modules and there will thus be the possibility of random fluctuations injected in choosing the next destination city at every decision point.

Note: Due to the generation of high exponents and large decimal arithmetic seen in equation (127), when the *weight* parameter exceeds 9, as per equation (129) the probability of choosing the shortest distance becomes 100%, i.e., nearest-neighbor algorithm without any random fluctuations is used.

**c.** Do higher quantities of Navigation Module B’s result in significantly better results?

Multiple runs to ensure statistical significance (or insignificance) of the following is to be examined: the shortest distance obtained by a CCA7 architecture using 1023 (i.e., “1K”) Navigation Module B’s versus versions of the architecture using 4,095 (i.e., “4K”) and 16,384 (i.e., “16K”) Navigation Module B’s.

Note: Navigation Module B n=0 is restricted for holding a copy of any instructions, and thus the availability of 1K-1, 4K-1 and 16K-1 Navigation Module B’s, which are rounded and simply referred to as “1K”, “4K” or “16K” respectively.

**d.** Does a “value weighted” algorithm to introduce random fluctuations (i.e., Section 4.2) give better results (i.e., a shorter distance) than the “position weighted” algorithm (i.e., equations (124) to (127))?

Multiple runs to ensure statistical significance (or insignificance) of the following is to be examined: the shortest distance obtained by the usual “position weighted” version of the CCA7 architecture using 1023 Navigation Module B’s versus the shortest distance obtained by “value weighted” version of the architecture.

Note: “Position weighted” refers to equations (124) to (127) which create a probability distribution of which location to choose next in deciding where to navigate to, simply in terms of the which location is the nearest, the next-nearest, the third-next-nearest, and so on, without considering the absolute or relative values of the distances. In contrast, “valued weighted” refers to the modifications of these equations given by Section 4.2 such that the reciprocals of the difference of each distance value from the smallest value (i.e., its actual value and the actual value of the smallest distance, rather than just its position of where it is relative to the other possible destinations) are calculated and normalized to create a probability distribution of which location to choose next in deciding where to navigate to.

.

|  | **0** | **1** | **2** | **3** | **4** | **5** | **6** | **7** | **8** | **9** | **10** | **11** | **12** |
| --- | --- | --- | --- | --- | --- | --- | --- | --- | --- | --- | --- | --- | --- |
| **0** | 0 | 2451 | 713 | 1018 | 1631 | 1374 | 2408 | 213 | 2571 | 875 | 1420 | 2145 | 1972 |
| **1** | 2451 | 0 | 1745 | 1524 | 831 | 1240 | 959 | 2596 | 403 | 1589 | 1374 | 357 | 579 |
| **2** | 713 | 1745 | 0 | 355 | 920 | 803 | 1737 | 851 | 1858 | 262 | 940 | 1453 | 1260 |
| **3** | 1018 | 1524 | 355 | 0 | 700 | 862 | 1395 | 1123 | 1584 | 466 | 1056 | 1280 | 987 |
| **4** | 1631 | 831 | 920 | 700 | 0 | 663 | 1021 | 1769 | 949 | 796 | 879 | 586 | 371 |
| **5** | 1374 | 1240 | 803 | 862 | 663 | 0 | 1681 | 1551 | 1765 | 547 | 225 | 887 | 999 |
| **6** | 2408 | 959 | 1737 | 1395 | 1021 | 1681 | 0 | 2493 | 678 | 1724 | 1891 | 1114 | 701 |
| **7** | 213 | 2596 | 851 | 1123 | 1769 | 1551 | 2493 | 0 | 2699 | 1038 | 1605 | 2300 | 2099 |
| **8** | 2571 | 403 | 1858 | 1584 | 949 | 1765 | 678 | 2699 | 0 | 1744 | 1645 | 653 | 600 |
| **9** | 875 | 1589 | 262 | 466 | 796 | 547 | 1724 | 1038 | 1744 | 0 | 679 | 1272 | 1162 |
| **10** | 1420 | 1374 | 940 | 1056 | 879 | 225 | 1891 | 1605 | 1645 | 679 | 0 | 1017 | 1200 |
| **11** | 2145 | 357 | 1453 | 1280 | 586 | 887 | 1114 | 2300 | 653 | 1272 | 1017 | 0 | 504 |
| **12** | 1972 | 579 | 1260 | 987 | 371 | 999 | 701 | 2099 | 600 | 1162 | 1200 | 504 | 0 |

Table B1. Traveling Salesperson Problem. Starting at city #0, the salesperson must travel to all the other cities and then return to city #0, taking the shortest total distance. The distance between the different cities #0 to #12 are given here in miles. (Google OR-Tools, 2023)

B.2 Results Data

| *weight* value | Shortest Distance (miles) Obtained from CCA7 with 1 Navigation Module B running position weighted algorithm (dataset from Table B1 used) | Shortest Distance (miles) Obtained from CCA7 with 1023 Navigation Module B’s running position weighted algorithm (dataset from Table B1 used) |
| --- | --- | --- |
| \| 1 \| \| --- \| \| 2 \| \| 3 \| \| 4 \| \| 5 \| \| 6 \| \| 7 \| \| 8 \| \| 9 \| \| 10 \| \| 11 \| \| 12 \| \| 13 \| \| 14 \| \| 15 \| \| 16 \| \| 17 \| \| 18 \| \| 19 \| \| 20 \| \| 21 \| \| 22 \| \| 23 \| \| 24 \| \| 25 \| \| 26 \| \| 27 \| \| 28 \| \| 29 \| \| 30 \| | \| 12197 \| \| --- \| \| 13846 \| \| 10152 \| \| 8871 \| \| 9964 \| \| 7653 \| \| 9475 \| \| 8251 \| \| 8893 \| \| 8131 \| \| 8131 \| \| 8131 \| \| 8131 \| \| 8131 \| \| 8131 \| \| 8131 \| \| 8131 \| \| 8131 \| \| 8131 \| \| 8131 \| \| 8131 \| \| 8131 \| \| 8131 \| \| 8131 \| \| 8131 \| \| 8131 \| \| 8131 \| \| 8131 \| \| 8131 \| \| 8131 \| | \| \| 8140 \| \| --- \| \| 7750 \| \| 7310 \| \| 7310 \| \| 7310 \| \| 7310 \| \| 7310 \| \| 7310 \| \| 7310 \| \| 8131 \| \| 8131 \| \| 8131 \| \| 8131 \| \| 8131 \| \| 8131 \| \| 8131 \| \| 8131 \| \| 8131 \| \| 8131 \| \| 8131 \| \| 8131 \| \| 8131 \| \| 8131 \| \| 8131 \| \| 8131 \| \| 8131 \| \| 8131 \| \| 8131 \| \| 8131 \| \| 8131 \| \| \| --- \| --- \| --- \| --- \| --- \| --- \| --- \| --- \| --- \| --- \| --- \| --- \| --- \| --- \| --- \| --- \| --- \| --- \| --- \| --- \| --- \| --- \| --- \| --- \| --- \| --- \| --- \| --- \| --- \| --- \| --- \| |

Table B2. Traveling Salesperson Problem. Comparison of CCA7 Architecture with 1 Navigation Module B running the position-weighted algorithm versus the architecture 1023 Navigation Module B’s running the position-weighted algorithm. Single runs. Traveling Salesperson Problem data is from Table B1.

| Shortest Distance (miles) Obtained from CCA7 with 1 Navigation Module B running position weighted algorithm (dataset from Table B1 used) x100 runs | Shortest Distance (miles) Obtained from CCA7 with 1 Navigation Module B running nearest-neighbor algorithm (dataset from Table B1 used) x100 runs | Shortest Distance (miles) Obtained from CCA7 with 1023 Navigation Module B’s running position weighted algorithm (dataset from Table B1 used) x100 runs |
| --- | --- | --- |
| number of runs: 100 mean = 9965.5 miles standard deviation = 1532.5 miles shortest path: 7647 miles % optimal (7293 miles) runs: 0% | number of runs: 100 mean = 8131.0 miles standard deviation = 0.0 miles shortest path: 8131 miles % optimal (7293 miles) runs: 0% | number of runs: 100 mean = 7432.2 miles standard deviation = 141.8 miles shortest path: 7293 miles % optimal (7293 miles) runs: 1% -versus 1 NavModB running position-weighted: p < 0.001 -versus 1 NavModB running nearest-neighbor: p < 0.001 (Welch’s 1-tail t-test) |
| \| 9150 \| \| --- \| \| 9492 \| \| 8120 \| \| 9343 \| \| 10101 \| \| 9576 \| \| 10547 \| \| 9007 \| \| 11641 \| \| 7728 \| \| 11050 \| \| 10728 \| \| 12040 \| \| 10200 \| \| 9213 \| \| 12538 \| \| 8185 \| \| 11131 \| \| 8614 \| \| 10445 \| \| 10870 \| \| 8003 \| \| 8279 \| \| 9114 \| \| 8700 \| \| 9044 \| \| 8812 \| \| 8716 \| \| 13008 \| \| 8540 \| \| 9520 \| \| 12605 \| \| 8692 \| \| 11210 \| \| 8131 \| \| 8837 \| \| 10391 \| \| 9452 \| \| 10934 \| \| 12222 \| \| 9982 \| \| 8258 \| \| 8665 \| \| 8490 \| \| 12013 \| \| 11297 \| \| 8785 \| \| 7647 \| \| 9960 \| \| 10423 \| \| 9615 \| \| 11368 \| \| 8049 \| \| 12759 \| \| 13601 \| \| 12685 \| \| 8354 \| \| 8992 \| \| 9243 \| \| 9459 \| \| 11150 \| \| 12884 \| \| 10963 \| \| 10419 \| \| 9034 \| \| 9120 \| \| 8895 \| \| 8834 \| \| 9184 \| \| 11597 \| \| 12538 \| \| 9509 \| \| 9132 \| \| 8354 \| \| 10902 \| \| 8781 \| \| 10218 \| \| 11784 \| \| 9003 \| \| 9999 \| \| 10480 \| \| 8544 \| \| 12939 \| \| 8536 \| \| 12911 \| \| 7917 \| \| 9053 \| \| 8965 \| \| 11713 \| \| 8898 \| \| 9773 \| \| 8816 \| \| 10454 \| \| 9169 \| \| 10435 \| \| 11944 \| \| 9834 \| \| 7678 \| \| 8884 \| \| 13734 \| | \| 8131 \| \| --- \| \| 8131 \| \| 8131 \| \| 8131 \| \| 8131 \| \| 8131 \| \| 8131 \| \| 8131 \| \| 8131 \| \| 8131 \| \| 8131 \| \| 8131 \| \| 8131 \| \| 8131 \| \| 8131 \| \| 8131 \| \| 8131 \| \| 8131 \| \| 8131 \| \| 8131 \| \| 8131 \| \| 8131 \| \| 8131 \| \| 8131 \| \| 8131 \| \| 8131 \| \| 8131 \| \| 8131 \| \| 8131 \| \| 8131 \| \| 8131 \| \| 8131 \| \| 8131 \| \| 8131 \| \| 8131 \| \| 8131 \| \| 8131 \| \| 8131 \| \| 8131 \| \| 8131 \| \| 8131 \| \| 8131 \| \| 8131 \| \| 8131 \| \| 8131 \| \| 8131 \| \| 8131 \| \| 8131 \| \| 8131 \| \| 8131 \| \| 8131 \| \| 8131 \| \| 8131 \| \| 8131 \| \| 8131 \| \| 8131 \| \| 8131 \| \| 8131 \| \| 8131 \| \| 8131 \| \| 8131 \| \| 8131 \| \| 8131 \| \| 8131 \| \| 8131 \| \| 8131 \| \| 8131 \| \| 8131 \| \| 8131 \| \| 8131 \| \| 8131 \| \| 8131 \| \| 8131 \| \| 8131 \| \| 8131 \| \| 8131 \| \| 8131 \| \| 8131 \| \| 8131 \| \| 8131 \| \| 8131 \| \| 8131 \| \| 8131 \| \| 8131 \| \| 8131 \| \| 8131 \| \| 8131 \| \| 8131 \| \| 8131 \| \| 8131 \| \| 8131 \| \| 8131 \| \| 8131 \| \| 8131 \| \| 8131 \| \| 8131 \| \| 8131 \| \| 8131 \| \| 8131 \| \| 8131 \| | \| 7594 \| \| --- \| \| 7310 \| \| 7310 \| \| 7569 \| \| 7581 \| \| 7586 \| \| 7653 \| \| 7647 \| \| 7653 \| \| 7310 \| \| 7310 \| \| 7310 \| \| 7310 \| \| 7343 \| \| 7293 \| \| 7310 \| \| 7310 \| \| 7310 \| \| 7310 \| \| 7586 \| \| 7586 \| \| 7590 \| \| 7310 \| \| 7647 \| \| 7310 \| \| 7310 \| \| 7310 \| \| 7586 \| \| 7310 \| \| 7639 \| \| 7343 \| \| 7647 \| \| 7310 \| \| 7590 \| \| 7310 \| \| 7343 \| \| 7310 \| \| 7586 \| \| 7586 \| \| 7310 \| \| 7310 \| \| 7586 \| \| 7318 \| \| 7343 \| \| 7586 \| \| 7310 \| \| 7586 \| \| 7586 \| \| 7310 \| \| 7343 \| \| 7343 \| \| 7586 \| \| 7310 \| \| 7312 \| \| 7586 \| \| 7622 \| \| 7586 \| \| 7310 \| \| 7310 \| \| 7619 \| \| 7320 \| \| 7310 \| \| 7619 \| \| 7586 \| \| 7343 \| \| 7310 \| \| 7581 \| \| 7594 \| \| 7310 \| \| 7310 \| \| 7310 \| \| 7586 \| \| 7310 \| \| 7343 \| \| 7573 \| \| 7310 \| \| 7310 \| \| 7343 \| \| 7343 \| \| 7310 \| \| 7619 \| \| 7622 \| \| 7310 \| \| 7343 \| \| 7320 \| \| 7343 \| \| 7318 \| \| 7343 \| \| 7310 \| \| 7619 \| \| 7674 \| \| 7310 \| \| 7310 \| \| 7619 \| \| 7598 \| \| 7343 \| \| 7586 \| \| 7312 \| \| 7586 \| \| 7312 \| |

Table B3. Traveling Salesperson Problem. Comparison of CCA7 Architecture with 1 Navigation Module B running the position-weighted algorithm, with 1 Navigation Module B running the nearest-neighbor algorithm, and with 1023 Navigation Module B’s running the position-weighted algorithm. Data from 100 runs of each variant of the architecture. Traveling Salesperson Problem data is from Table B1.


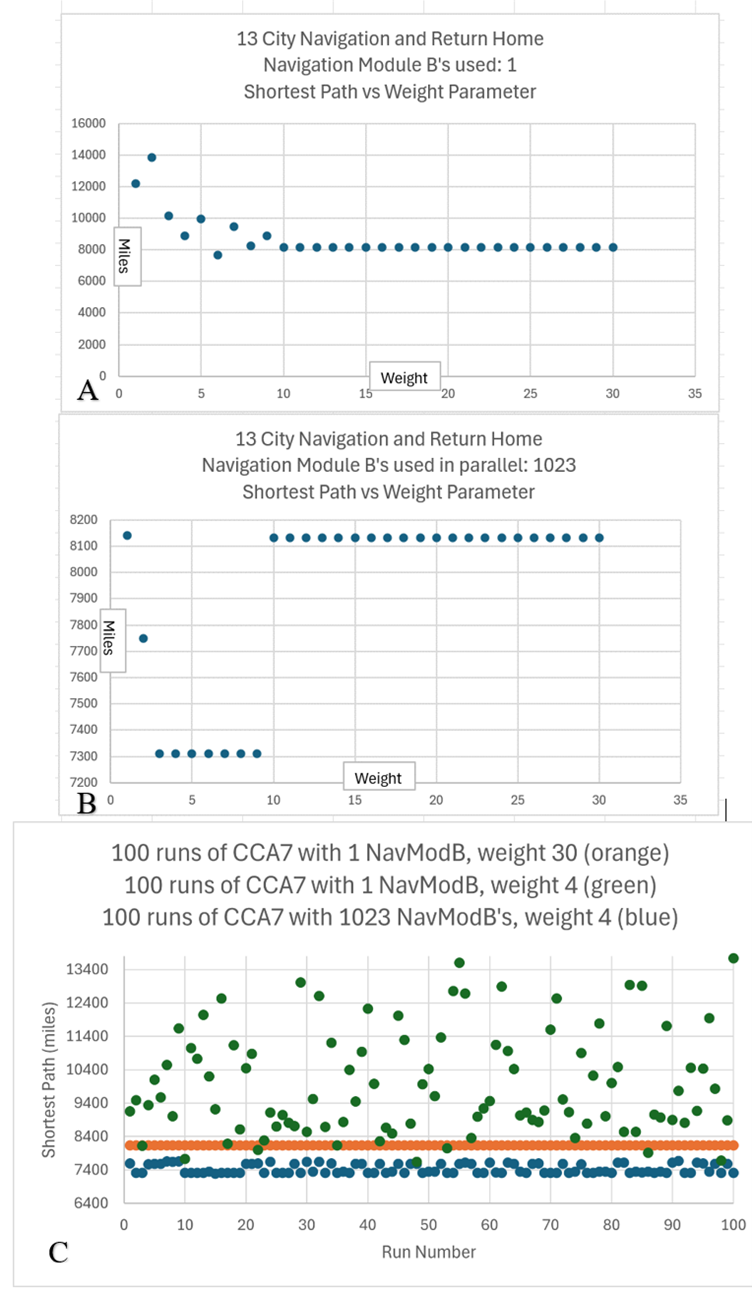


Figure B1 (Figures 13, 14 in main text). Simulation of the CCA7 robot navigating from a home city to twelve other cities (13 cities in total) and then returning home. The *weight* “wt” parameter of equations (126, 128) is adjusted from a value of *weight* = 1 to *weight*=30 (no units). Lower values of *weight* introduce more randomness in choosing the next destination city. The shortest length (miles in the dataset used) traveled between each city and then returning home is recorded.
**A.** Only a single Navigation Module B is used, so rather than restrict it to equations (118, 119) (which will yield each time a value of 8131 miles per the nearest-neighbor algorithm) it is running equations (120, 121), i.e., small_plan(random=weight_distance) where a random fluctuation is introduced to the nearest neighbor algorithm (124–127).
**B.** The results are shown when the full set of Navigation Module B’s n=1…1023 (i.e., 1023 NavModB’s in parallel) are used. Here too, all modules from n=1 to n=1023 were allowed to follow equations (120, 121), i.e., small_plan(random=weight_distance) where a random fluctuation is introduced to the nearest neighbor algorithm. At higher values of the *weight* parameter, the CCA7 follows the nearest neighbor algorithm in deciding which city to navigate to next—a value of 8131 miles is expected for the data set used. At lower values of the *weight* parameter, there are more random fluctuations introduced in choosing the next city to navigate to (equations (124–129)). Note that in **A** where only a single Navigation Module B is used, the extra randomness generally did not help to yield a shorter path than the nearest neighbor algorithm. Note that in **B** where 1023 Navigation Module B’s were used in parallel, the randomness provided within a range of the *weight* parameters, helped to yield shorter paths than obtained via the nearest neighbor algorithm, approaching the theoretical shortest path of 7293 miles.
**C.** 100 simulated runs with the same dataset as above when there is only one Navigation Module B used, with a weight of 30, i.e., small_plan(random=weight_distance), where there is only one Navigation Module B used, with a weight of 4, i.e., small_plan(random= weight_distance), and where there are 1023 Navigation Module B’s used, with a weight of 4, i.e., small_plan(random=weight_distance). Note that when there is only one Navigation Module B used, the nearest neighbor algorithm without any random fluctuations gives the best results. However, if the full 1023 Navigation Module B’s are used, the random fluctuations are advantageous in yielding shorter paths.

| Shortest Distance (miles) Obtained from CCA7 with 1023 (“1K”) Navigation Module B’s running position weighted algorithm (dataset from Table B1 used) x100 runs | Shortest Distance (miles) Obtained from CCA7 with 4K Navigation Module B’s running position weighted algorithm (dataset from Table B1 used) x100 runs | Shortest Distance (miles) Obtained from CCA7 with 16K Navigation Module B’s running position weighted algorithm (dataset from Table B1 used) x100 runs |
| --- | --- | --- |
| number of runs: 100 mean = 7432.2 miles standard deviation = 141.8 miles shortest path: 7293 miles % optimal (7293 miles) runs: 1% | number of runs: 100 mean = 7309.1 miles standard deviation = 29.8 miles shortest path: 7293 miles % optimal (7293 miles) runs: 27%  -versus 1K NavModB’s  p < 0.001 (Welch’s 1-tail t-test) | number of runs: 100 mean = 7296.8 miles standard deviation = 6.9 miles shortest path: 7293 miles % optimal (7293 miles) runs: 67%  -versus 4K NavModB’s  p < 0.001 (Welch’s 1-tail t-test) |
| \| 7594 \| \| --- \| \| 7310 \| \| 7310 \| \| 7569 \| \| 7581 \| \| 7586 \| \| 7653 \| \| 7647 \| \| 7653 \| \| 7310 \| \| 7310 \| \| 7310 \| \| 7310 \| \| 7343 \| \| 7293 \| \| 7310 \| \| 7310 \| \| 7310 \| \| 7310 \| \| 7586 \| \| 7586 \| \| 7590 \| \| 7310 \| \| 7647 \| \| 7310 \| \| 7310 \| \| 7310 \| \| 7586 \| \| 7310 \| \| 7639 \| \| 7343 \| \| 7647 \| \| 7310 \| \| 7590 \| \| 7310 \| \| 7343 \| \| 7310 \| \| 7586 \| \| 7586 \| \| 7310 \| \| 7310 \| \| 7586 \| \| 7318 \| \| 7343 \| \| 7586 \| \| 7310 \| \| 7586 \| \| 7586 \| \| 7310 \| \| 7343 \| \| 7343 \| \| 7586 \| \| 7310 \| \| 7312 \| \| 7586 \| \| 7622 \| \| 7586 \| \| 7310 \| \| 7310 \| \| 7619 \| \| 7320 \| \| 7310 \| \| 7619 \| \| 7586 \| \| 7343 \| \| 7310 \| \| 7581 \| \| 7594 \| \| 7310 \| \| 7310 \| \| 7310 \| \| 7586 \| \| 7310 \| \| 7343 \| \| 7573 \| \| 7310 \| \| 7310 \| \| 7343 \| \| 7343 \| \| 7310 \| \| 7619 \| \| 7622 \| \| 7310 \| \| 7343 \| \| 7320 \| \| 7343 \| \| 7318 \| \| 7343 \| \| 7310 \| \| 7619 \| \| 7674 \| \| 7310 \| \| 7310 \| \| 7619 \| \| 7598 \| \| 7343 \| \| 7586 \| \| 7312 \| \| 7586 \| \| 7312 \| | \| 7310 \| \| --- \| \| 7310 \| \| 7295 \| \| 7320 \| \| 7293 \| \| 7310 \| \| 7310 \| \| 7310 \| \| 7310 \| \| 7310 \| \| 7310 \| \| 7293 \| \| 7310 \| \| 7310 \| \| 7293 \| \| 7310 \| \| 7310 \| \| 7310 \| \| 7310 \| \| 7295 \| \| 7310 \| \| 7310 \| \| 7293 \| \| 7310 \| \| 7310 \| \| 7310 \| \| 7310 \| \| 7310 \| \| 7293 \| \| 7310 \| \| 7310 \| \| 7310 \| \| 7310 \| \| 7293 \| \| 7310 \| \| 7293 \| \| 7310 \| \| 7293 \| \| 7310 \| \| 7293 \| \| 7293 \| \| 7293 \| \| 7310 \| \| 7293 \| \| 7310 \| \| 7310 \| \| 7310 \| \| 7310 \| \| 7310 \| \| 7310 \| \| 7310 \| \| 7310 \| \| 7320 \| \| 7310 \| \| 7293 \| \| 7293 \| \| 7293 \| \| 7310 \| \| 7293 \| \| 7295 \| \| 7310 \| \| 7312 \| \| 7343 \| \| 7310 \| \| 7310 \| \| 7293 \| \| 7293 \| \| 7310 \| \| 7312 \| \| 7312 \| \| 7293 \| \| 7586 \| \| 7310 \| \| 7310 \| \| 7310 \| \| 7310 \| \| 7343 \| \| 7310 \| \| 7293 \| \| 7310 \| \| 7310 \| \| 7293 \| \| 7310 \| \| 7293 \| \| 7310 \| \| 7293 \| \| 7318 \| \| 7310 \| \| 7310 \| \| 7310 \| \| 7310 \| \| 7310 \| \| 7310 \| \| 7293 \| \| 7293 \| \| 7293 \| \| 7343 \| \| 7310 \| \| 7293 \| \| 7310 \| | \| 7310 \| \| --- \| \| 7293 \| \| 7293 \| \| 7293 \| \| 7293 \| \| 7293 \| \| 7310 \| \| 7293 \| \| 7310 \| \| 7293 \| \| 7310 \| \| 7293 \| \| 7293 \| \| 7293 \| \| 7295 \| \| 7293 \| \| 7293 \| \| 7293 \| \| 7293 \| \| 7295 \| \| 7293 \| \| 7293 \| \| 7293 \| \| 7293 \| \| 7295 \| \| 7295 \| \| 7310 \| \| 7293 \| \| 7293 \| \| 7293 \| \| 7310 \| \| 7310 \| \| 7293 \| \| 7293 \| \| 7310 \| \| 7293 \| \| 7293 \| \| 7293 \| \| 7310 \| \| 7293 \| \| 7293 \| \| 7310 \| \| 7293 \| \| 7293 \| \| 7293 \| \| 7293 \| \| 7295 \| \| 7295 \| \| 7293 \| \| 7293 \| \| 7293 \| \| 7310 \| \| 7310 \| \| 7293 \| \| 7310 \| \| 7293 \| \| 7310 \| \| 7293 \| \| 7293 \| \| 7295 \| \| 7293 \| \| 7293 \| \| 7310 \| \| 7293 \| \| 7293 \| \| 7293 \| \| 7310 \| \| 7295 \| \| 7293 \| \| 7310 \| \| 7293 \| \| 7293 \| \| 7295 \| \| 7293 \| \| 7293 \| \| 7293 \| \| 7293 \| \| 7293 \| \| 7310 \| \| 7293 \| \| 7293 \| \| 7293 \| \| 7310 \| \| 7295 \| \| 7293 \| \| 7310 \| \| 7293 \| \| 7295 \| \| 7310 \| \| 7293 \| \| 7293 \| \| 7293 \| \| 7293 \| \| 7293 \| \| 7293 \| \| 7295 \| \| 7293 \| \| 7293 \| \| 7293 \| \| 7293 \| |

Table B4. Traveling Salesperson Problem. Comparison of CCA7 Architectures all running the position-weighted algorithm but with 1K (actually 1K-1, or, 1023) Navigation Module B’s, with 4K Navigation Module B’s and with 16K Navigation Module B’s. Data from 100 runs of each variant of the architecture. Traveling Salesperson Problem data is from Table B1.


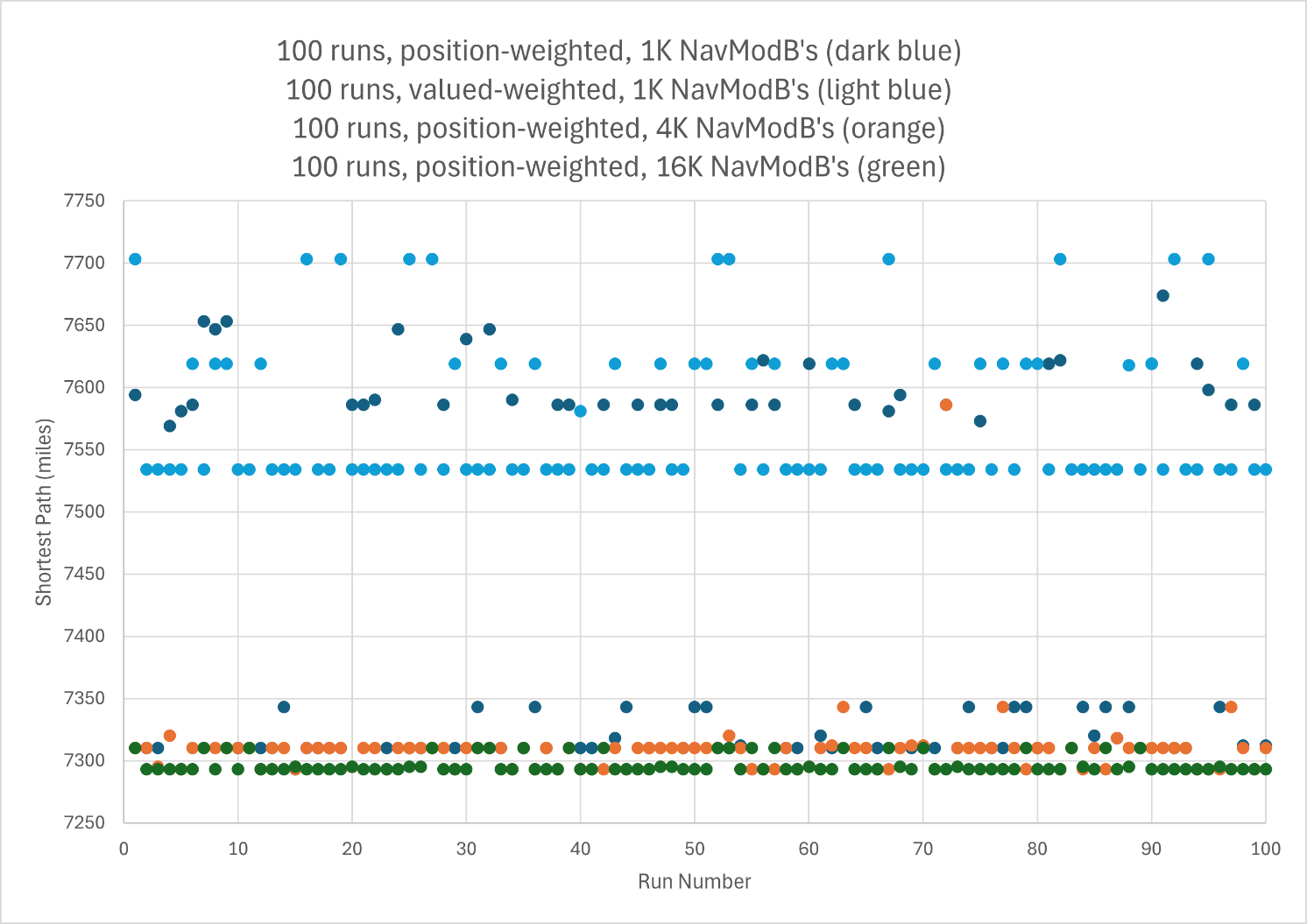


Figure B2 (Figure 15 in main text). Traveling Salesperson Problem. Comparison of three CCA7 Architectures running the position-weighted algorithm but with 1K Navigation Module B’s (dark blue dots), with 4K Navigation Module B’s (orange dots), and with 16K Navigation Module B’s (green dots). There is also comparison with the architecture with 1K Navigation Module B’s but running a value-weighted algorithm (light blue dots). Data from 100 runs of each variant of the architecture. Traveling Salesperson Problem data is from Table B1.

| Shortest Distance (miles) Obtained from CCA7 with 1023 Navigation Module B’s running position weighted algorithm (dataset from Table B1 used) x100 runs | Shortest Distance (miles) Obtained from CCA7 with 1023 Navigation Module B’s running value-weighted algorithm (dataset from Table B1 used) x100 runs |
| --- | --- |
| number of runs: 100 mean = 7432.2 miles standard deviation = 141.8 miles shortest path: 7293 miles % optimal (7293 miles) runs: 1%  -versus 1 NavModB running value-weighted: p < 0.001 (Welch’s 1-tail t-test) | number of runs: 100 mean = 7572.6 miles standard deviation = 58.0 miles shortest path: 7534 miles % optimal (7293 miles) runs: 0% |
| \| 7594 \| \| --- \| \| 7310 \| \| 7310 \| \| 7569 \| \| 7581 \| \| 7586 \| \| 7653 \| \| 7647 \| \| 7653 \| \| 7310 \| \| 7310 \| \| 7310 \| \| 7310 \| \| 7343 \| \| 7293 \| \| 7310 \| \| 7310 \| \| 7310 \| \| 7310 \| \| 7586 \| \| 7586 \| \| 7590 \| \| 7310 \| \| 7647 \| \| 7310 \| \| 7310 \| \| 7310 \| \| 7586 \| \| 7310 \| \| 7639 \| \| 7343 \| \| 7647 \| \| 7310 \| \| 7590 \| \| 7310 \| \| 7343 \| \| 7310 \| \| 7586 \| \| 7586 \| \| 7310 \| \| 7310 \| \| 7586 \| \| 7318 \| \| 7343 \| \| 7586 \| \| 7310 \| \| 7586 \| \| 7586 \| \| 7310 \| \| 7343 \| \| 7343 \| \| 7586 \| \| 7310 \| \| 7312 \| \| 7586 \| \| 7622 \| \| 7586 \| \| 7310 \| \| 7310 \| \| 7619 \| \| 7320 \| \| 7310 \| \| 7619 \| \| 7586 \| \| 7343 \| \| 7310 \| \| 7581 \| \| 7594 \| \| 7310 \| \| 7310 \| \| 7310 \| \| 7586 \| \| 7310 \| \| 7343 \| \| 7573 \| \| 7310 \| \| 7310 \| \| 7343 \| \| 7343 \| \| 7310 \| \| 7619 \| \| 7622 \| \| 7310 \| \| 7343 \| \| 7320 \| \| 7343 \| \| 7318 \| \| 7343 \| \| 7310 \| \| 7619 \| \| 7674 \| \| 7310 \| \| 7310 \| \| 7619 \| \| 7598 \| \| 7343 \| \| 7586 \| \| 7312 \| \| 7586 \| \| 7312 \| | \| 7703 \| \| --- \| \| 7534 \| \| 7534 \| \| 7534 \| \| 7534 \| \| 7619 \| \| 7534 \| \| 7619 \| \| 7619 \| \| 7534 \| \| 7534 \| \| 7619 \| \| 7534 \| \| 7534 \| \| 7534 \| \| 7703 \| \| 7534 \| \| 7534 \| \| 7703 \| \| 7534 \| \| 7534 \| \| 7534 \| \| 7534 \| \| 7534 \| \| 7703 \| \| 7534 \| \| 7703 \| \| 7534 \| \| 7619 \| \| 7534 \| \| 7534 \| \| 7534 \| \| 7619 \| \| 7534 \| \| 7534 \| \| 7619 \| \| 7534 \| \| 7534 \| \| 7534 \| \| 7581 \| \| 7534 \| \| 7534 \| \| 7619 \| \| 7534 \| \| 7534 \| \| 7534 \| \| 7619 \| \| 7534 \| \| 7534 \| \| 7619 \| \| 7619 \| \| 7703 \| \| 7703 \| \| 7534 \| \| 7619 \| \| 7534 \| \| 7619 \| \| 7534 \| \| 7534 \| \| 7534 \| \| 7534 \| \| 7619 \| \| 7619 \| \| 7534 \| \| 7534 \| \| 7534 \| \| 7703 \| \| 7534 \| \| 7534 \| \| 7534 \| \| 7619 \| \| 7534 \| \| 7534 \| \| 7534 \| \| 7619 \| \| 7534 \| \| 7619 \| \| 7534 \| \| 7619 \| \| 7619 \| \| 7534 \| \| 7703 \| \| 7534 \| \| 7534 \| \| 7534 \| \| 7534 \| \| 7534 \| \| 7618 \| \| 7534 \| \| 7619 \| \| 7534 \| \| 7703 \| \| 7534 \| \| 7534 \| \| 7703 \| \| 7534 \| \| 7534 \| \| 7619 \| \| 7534 \| \| 7534 \| |

Table B5. Traveling Salesperson Problem. Comparison of two CCA7 Architectures each with 1023 (i.e., “1K”) Navigation Module B’s, with one variant running the position-weighted algorithm and the other variant running the value-weighted algorithm. Data from 100 runs of each variant of the architecture. Traveling Salesperson Problem data is from Table B1.
